# Wnt 3a Protects Myocardial Injury in Elderly Acute Myocardial Infarction by Inhibiting Serum Cystatin C/ROS-Induced Mitochondrial Damage

**DOI:** 10.3389/fphys.2022.950960

**Published:** 2022-07-22

**Authors:** Jian Shen, Ying Li, Yang Jiao, Jihang Wang, Xiaoling Hou, Yongkang Su, Bing Liu, Henan Liu, Zhijun Sun, Qing Xi, Zhenhong Fu

**Affiliations:** ^1^ Senior Department of Cardiology, The Sixth Medical Center, Chinese PLA General Hospital and Chinese PLA Medical School, Beijing, China; ^2^ Outpatient Department of Tongzhou Retired Cadres Rest Center, Beijing, China; ^3^ The First Medical Center, Chinese PLA General Hospital, Beijing, China

**Keywords:** Wnt/β-catenin, Cystatin C/ROS signaling pathway, elderly acute myocardial infarction, myocardial injury, mitochondrial damage

## Abstract

Aging represents an independent risk factor affecting the poor prognosis of patients with acute myocardial infarction (AMI). This present research aimed to explore the molecular mechanism of myocardial injury in elderly AMI by animals and cells experiment. Our previous clinical study found the serum Cystatin C (Cys-C) increased in the elderly AMI population, while the mechanism underlying high Cys-C induced myocardial injury of AMI remains unclear. In the *in-vitro* study, we confirmed that Wnt/β-catenin could significantly reduce the expression of cytoplasmic Cys-C through transnuclear action, and highly attenuate the occurrence of mitochondrial oxidative stress injury induced *via* Cys-C/reactive oxygen species (ROS). Furthermore, the addition of exogenous Wnt3a and inhibition of Cys-C expression could effectively inhibit mitochondrial oxidative stress injury and relieve the acute myocardial hypoxia injury. These results indicate that Cys-C exerted damaging effects on the hypoxic aging cardiomyocyte through the ROS/mitochondrial signaling pathway. Inhibition of this pathway effectively reduced the apoptosis of aging cardiomyocytes. In the *in-vivo* study, we also explored the function of the Wnt/Cys-C pathway on the ischemic infarction heart. We confirmed that Wnt/β-catenin served as the upstream protective protein of this pathway, and the promotion of this pathway improved the cardiac structure and function of the elderly AMI mice effectively.

## Introduction

AMI refers to a clinical or pathological event with evidence of myocardial injury due to acute myocardial ischemia. Although the early applications of both coronary intervention and bypass surgery reduce the immediate mortality of patients, the incidence of long-term heart failure and recurrent myocardial infarction has still been increasing year by year, creating a major disease burden on the Chinese population. Moreover, senescence represents an independent risk factor affecting the prognosis of AMI patients (Ishii et al., 2020; Cammalleri et al., 2021). There are also high rates of delayed diagnosis and treatment, high risks of intervention and surgery, and high mortality and poor prognosis in aging AMI patients. Our previous studies showed that the GRACE (The Global Registry of Acute Coronary Events) score was extremely low value in the evaluation of the prognosis of elderly AMI patients (Fu et al., 2018). Therefore, it is urgent to seek an effective indicator to evaluate the severity and prognosis of this disease and to explore its specific molecular mechanisms to conduct effective preventions and treatments for elderly AMI patients.

Cys-C is a high-purity cysteine protease inhibitor isolated and purified in an egg. Recent studies have shown that Cys-C plays an important role in the development of both atherosclerosis (Dong and Nao, 2019; Fatemi et al., 2019) and cardiovascular events (Fu et al., 2013; Correa et al., 2018; Budano et al., 2020; Ma et al., 2020; Lu et al., 2021). However, the fundamental specific mechanisms of regulation *via* Cys-C underlying severe myocardial injury and poor prognosis in aging patients with AMI remains unclear.

Wnt is an essential protein for the genesis and development of embryos, regulating the proliferation, differentiation, polarity, and migration of cells. The classical Wnt signaling pathway is as follows: Wnt proteins and the Frizzled–LRP5/6 receptor activate Dishevelled (Dvl) protein, and then induce the dissociation of β-catenin from the complex. The dephosphorylated β-catenin accumulates in the cytoplasm and is transferred into the nucleus, promoting a series of factors expression like Cyclin D1 or other cell survival factors (Działo et al., 2019; Zhu and Lu, 2019; Zeng and Bao, 2021).

Wnt plays important role in the pathological and physiological process of myocardial infarction (Yang et al., 2017; Fan et al., 2018; Srivastava et al., 2021). Previous studies have shown that decreased Wnt/β-catenin and increased ROS production were involved in cardiomyocytes’ apoptosis and deteriorated cardiac contractile function under stress conditions (Heo and Lee, 2011; Zhu and Lu, 2019). Blocking the production of reactive oxygen species**(**ROS) can reduce cardiomyocytes’ apoptosis and improve cardiac systolic function (Wang et al., 2016). Furthermore, supplementation of Wnt3a can reduce the size of infarction areas after myocardial infarction (Tang et al., 2019). Other studies found the mechanism to reduce ischemia damage may be related to the promotion of nuclear translocation of β-catenin after AMI.

Cellular senescence is a major hallmark of aging and the Wnt/β-catenin pathway was shown to be an early warning to the activation of cellular senescence (Lehmann et al., 2020). Recent studies indicate that Wnt3a signaling might be a predominant factor that could be used to overcome senescence, which contributes to its proliferative, repair, and antioxidant capacity (Jeoung et al., 2015). Based on the previous research and the pathophysiology underlying ischemic cardiac disease in older patients, we postulate that the dysfunctional regulation of the Wnt/β-catenin pathway induced overexpression of Cys-C proteins which finally activated the cardiomyocyte oxidative damage pathway.

Therefore, our study aims to detect the molecular mechanisms between the Wnt/β-catenin and Cys-C pathway and their cross-talk functions for cell survival, which can help elucidate the causes for myocardial injury in cellular senescence, and their guiding significance for the treatment of elderly AMI patients in the clinical setting.

## Material and Method

### Animal Model of Cardiac Ischemic Injury *in vivo*

All animal procedures described in this study were approved by the Ethical Committee of Chinese PLA General Hospital Animal Care Center and Use Committee Center and were performed by the Guide for the Care and Use of Laboratory Animals (published by the US National Institutes of Health, NIH Publication No. 85-23, revised 1996). Male senescence-accelerated aging mice (SAMP8, SAMp8/TaHsd) and the wild-C57BL/6 mice (control group) were purchased from Research Science Company (R&S, Beijing) of 8 weeks of age. 24 weeks was decided to be set up as the senescence model (Gevaert et al., 2017). All mice were exposed to a 12-h light/dark cycle, with food and water provided. All mice were bred in the C57BL/6 background for at least three generations. Subsequently, 8-week-old wild-type (WT) mice (control) and 24-week-old senescence-accelerated aging mice (SAMP8) were used to induce an ischemia injury model according to previous studies. For the heart infarction procedure (the *in-vivo* experiment) the mice were anesthetized with isoflurane, and the hearts were exposed *via* a left thoracotomy. A 7-0 silk suture was used to ligate the left anterior descending coronary artery (LAD) for 4 h to mimic the ischemic procedure, and the blood samples were collected to evaluate myocardial enzyme markers, such as lactate dehydrogenase (LDH), troponin T, and CK-MB. Pretreatment of Wnt3a 20 ng/ml *via* caudal vein 2 h before the heart ischemic model. Infarcted size was measured using 4% Evans blue and 2,3,5-triphenyltetrazolium chloride (TTC) methods in different groups. Hematoxylin-eosin (HE) staining is used to evaluate cardiomyocyte structure and function in different groups.

### Hypoxia of Cardiomyocyte Injury *in vitro*

Cardiomyocytes were isolated from SAMP8 mice and WT mice. Subsequently, the hypoxia model was built in different groups. Specifically, to establish hypoxia injury, primary cardiomyocytes were culture in FBS (-) normal L-DMEM solution. Culture dishes were put into the hypoxia chamber with 5% CO_2_ and 95% N_2_ for 6 h. Cardiomyocytes were placed into a new solution containing 20% FBS and normal L-DMEM. For the pretreatment, Wnt3a 20 ng/ml was used 1 h before the model set up.

### Echocardiography Measurements

Echocardiography was performed in all mice at 10 h after the ischemia model finished to ascertain the hemodynamic stability of all mice. Left ventricular ejection fraction (LVEF) and left ventricular dilated dimension (LVDd) were measured in M-mode images using computer algorithms.

### Immunohistochemistry and TUNEL Staining

After different treatments, tissues were immersed in 4% paraformaldehyde for 4 h and transferred to 70% ethanol. Then, tissue was placed in cassettes, dehydrated through a serial alcohol gradient, and embedded in paraffin wax blocks. Finally, the tissue was stained with hematoxylin and eosin. Histopathological examination was performed in infarcted tissue on formalin-fixed, paraffin-embedded 5 to 6 μm sections stained with hematoxylin and eosin using the standard methods and examined *via* light microscopy. Antibodies used in this study were as follows: Cys-C (1:400, Abcam, #ab109508), a TUNEL assay was applied to observe the necroptosis according to the manufacturer’s protocol (R&D systems).

### Reactive Oxygen Species Measurement

Cytoplasmic cell ROS level were detected by DCFHDA (1:1000, Beyotime, Beijing, China) and MitoSOX (1:1000, Molecular Probes) assays which were conducted by the instructions. The cells were washed by PBS three times and incubated in dark with diluted DCFHDA at 37°C for 30 min. After that, the dishes were washed with PBS three times and incubated with DAPI (Sigma-Aldrich, United States) for 5 min and then captured the images under the microscope and analyzed with the postprocessing software ImageJ. As for mitochondrial ROS measurement, cardiomyocytes were resuspended in cold PBS at room temperature. Then, 10 μmol/L MitoSOX Red mitochondrial superoxide indicator (Molecular Probes, United States) was used for incubation with the cells for 30 min at 37°C. Washing with PBS three times, the mitochondrial ROS production was detected by flow cytometry as previously stated.

### Western Blot

After being treated, different groups of samples were washed with cold PBS and incubated with RIPA buffer containing protease inhibitor cocktail. Lysates were centrifuged at 12,000 g for 10 min. Protein concentration was quantified by BCA Protein Assay. The protein samples were separated by SDS-PAGE and transferred to PVDF membranes. Membranes were blocked with 5% fat-free milk in TBST buffer for 90 min and incubated overnight at 4°C with primary antibodies. The primary antibodies were showed as follows: Cys-C (1:1000, Abcam, #ab109508), β-catenin (1:1000, Abcam, #ab16051),α-actin (1:1000, Abcam, #ab179467). After washing with TBST, the membranes were incubated with horseradish peroxidase-coupled secondary antibodies for 2 h at room temperature. The secondary antibody was α-actin (1:1000, Abcam, #ab179467). After all procedures, the images were taken with enhanced chemiluminescence (ECL) reagent and exposure in the dark areas. All data were reproduced three times and analyzed by ImageJ software.

### Transmission Electron Microscopy

Transmission electron microscopy (TEM) was used to observe the structure of mitochondria in response to ischemic injury. For the electron microscopy, samples were dehydrated using acetonitrile and graded methanol, embedded in epoxy resin (EMbed-812; Electron Microscopy Sciences, United States), and polymerized at 70°C overnight. Hitachi H600 Electron Microscope (Hitachi, Japan) was used to capture the images.

### Mitochondrial Membrane Potential Measurement

The mitochondrial membrane potential was measured according to the manufacturer’s instructions using a mitochondrial membrane potential assay kit with JC-1 (cat. no. C2006, Beyotime). Images were captured with a fluorescence microscope (OLYMPUS DX51; Olympus, Tokyo, Japan) and quantified with Image-Pro Plus 6.0 (Media Cybernetics, Rockville, MD, United States) to obtain the mean densities of the regions of interest, which were normalized to those in the control group.

### Statistical Analysis

The data were described as the mean ± standard deviation (SD) of at least three independent experiments and was analyzed by one-way analysis of variance (ANOVA) among different groups. The statistical significance between the treated and control groups was *p* < 0.05.

## Result

### Wnt3a Enhanced Cardiac Structure and Function in Mouse Myocardial Infarction Model

**FIGURE 1:**
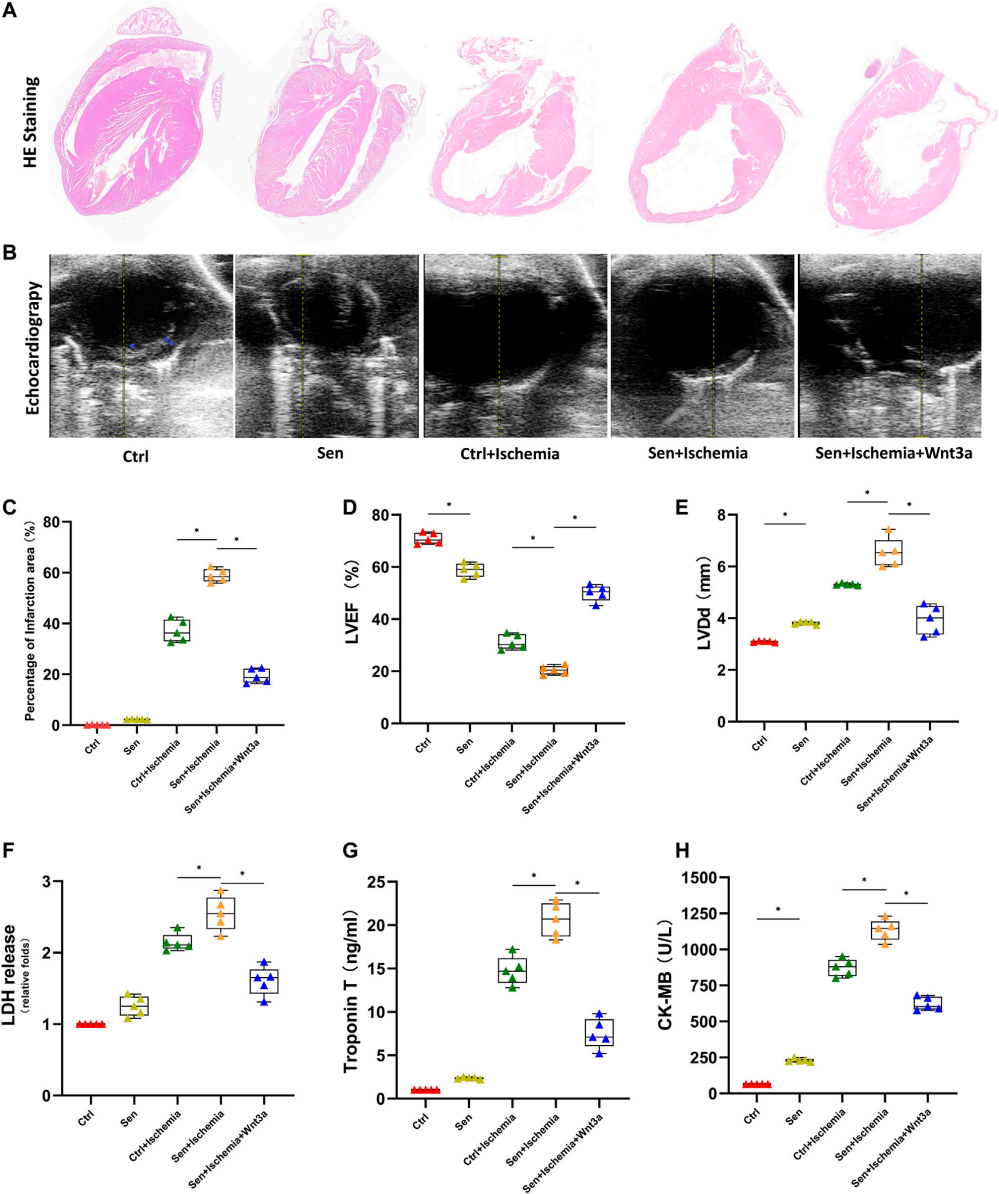
Effects of Wnt 3a on structural, functional and enzymatic changes of senile AMI hearts. Representative HE stained images of the hearts of mice in different groups. The Sen + Ischemia + Wnt3a group demonstrated that the cardiomyocyte structure and left ventricular free wall were improved to some extent compared with the Sen + Ischemia group **(A)**. Representative M-mode images by echocardiography in different groups, the Sen + Ischemia + Wnt3a group showed significantly improved cardiac function compared with Sen + Ischemia group **(B)**. Statistical analysis of the hearts of mice in different groups, the Sen + Ischemia + Wnt3a group demonstrated approximately 20% infarct size decrease due to ischemic injury than that in the Sen + Ischemia group (*p* < 0.05) **(C)**. Statistics of LVEF **(D)**, and LVDd **(E)** measured by echocardiography in different groups, the Sen + Ischemia + Wnt3a group showed significantly improved LVEF and decreased LVDd compared with Sen + Ischemia group (*p* < 0.05). Quantification of LDH **(F)**, Troponin T **(G)** and CK-MB **(H)** in different groups, the Sen + Ischemia + Wnt3a group showed a significant decrease in LDH, cTnT, and CK-MB levels compared with the Sen + Ischemia group (*p* < 0.05). HE, Hematoxylin-eosin; Ctrl: wild type mouse group; Sen: senile wild mouse group; Ctrl + Ischemia: wild type mouse AMI group; Sen + Ischemia: senile wild type mouse AMI group; Sen + Ischemia + Wnt3a: senile wild type mouse AMI group pretreated with Wnt3a 20 ng/ml. LVEF, left ventricular ejection fraction LVDd, left ventricular end diastolic dimension. ∗*p* < 0.05.

AMI models were established for wild-type mice and senile wild-type mice. Hematoxylin-eosin (HE) staining is used to evaluate cardiomyocyte structure and function in different groups (Figure 1A). The cardiomyocyte structure and left ventricular free wall thickness were significantly reduced in both the ischemia groups compared with the wild-type mice (Ctrl group) and senile wild-type mice (Sen group). The senile wild-type mice pretreated with Wnt3a (Sen + Ischemia + Wnt3a) demonstrated that the cardiomyocyte structure and left ventricular free wall were improved to some extent. The TTC and Evans blue staining were used to indicate Ctrl group and Sen group, the TTC staining showed no significant difference in the myocardial ischemic area with infarct size smaller than 5%; the wild type mouse ischemia group (Ctrl + Ischemia) demonstrated approximately 35% infarct size which was significantly larger compared with the Ctrl group; the experiment found approximately 60% infarct size in the senile wild type mouse ischemia group (Sen + Ischemia) showing a statistically significant increase compared to the Ctrl + Ischemia group. The Sen + Ischemia + Wnt3a group demonstrated approximately 20% infarct size due to ischemic injury with significantly decreased lesion area than that in the Sen + Ischemia group (*p* < 0.05) (Figure 1C).

LVEF and end-diastolic diameter were measured using echocardiography (Figures 1B,D,E) across groups to reflect the differences in systolic and diastolic function since the establishment of AMI models. The results suggested that there were statistical differences in systolic and diastolic function between the Ctrl and Sen groups. The Ctrl + Ischemia group demonstrated statistically significant decrease in cardiac function compared with both Ctrl and Sen groups. Subsequent experiments revealed further decreased cardiac function in the Sen + Ischemia group compared with the Ctrl + Ischemia group. However, the Sen + Ischemia + Wnt3a group showed significantly improved cardiac function compared with Sen + Ischemia group (*p* < 0.05).

Blood samples were collected from the caudal vein to analyze the biomarkers associated with myocardial infarction (Figures 1F–H). The results suggested that there were no significant differences in both LDH and cTnT levels in the blood between the Ctrl group and the Sen group, while the latter showed a significant increase in CK-MB level (*p* < 0.05). The Ctrl + Ischemia group demonstrated a statistically significant increase in LDH, cTnT, and CK-MB levels compared with either Ctrl group. Subsequent experiments revealed a further increase in myocardial infarction biomarkers for the Sen + Ischemia group compared with the Ctrl + Ischemia group. However, the Sen + Ischemia + Wnt3a group showed a significant decrease in LDH, cTnT, and CK-MB levels compared with the Sen + Ischemia group (*p* < 0.05), but it still presented with higher levels of these biomarkers relative to the Ctrl group. (*p* < 0.05).

### Wnt3a Decreased Cardiac Microcirculation Dysfunction in Mouse Myocardial Infarction Model

**FIGURE 2:**
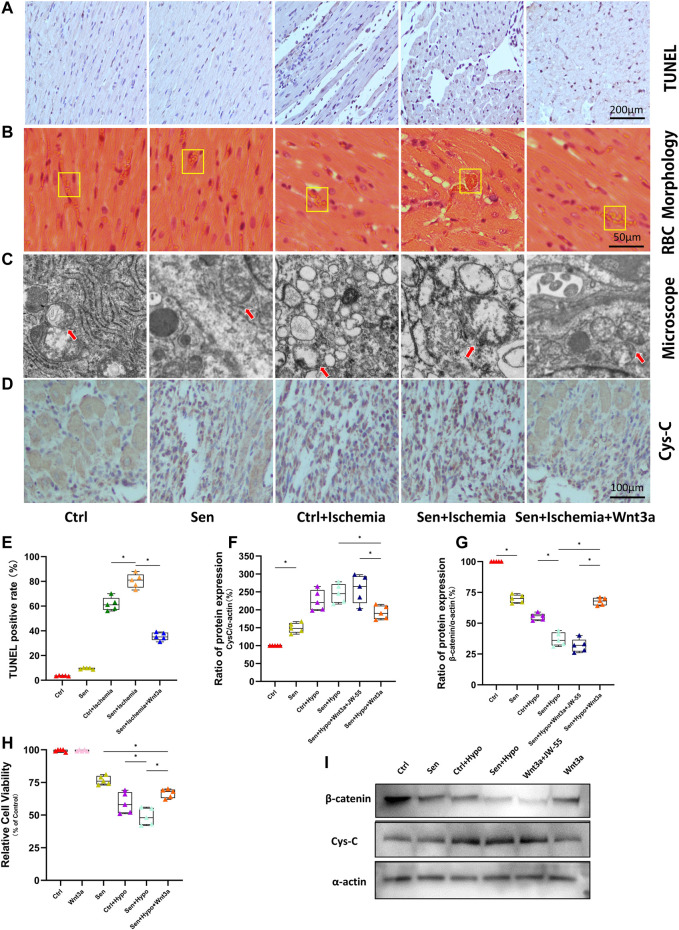
Effects of Wnt-3a on myocardial apoptosis, microcirculation, mitochondrial morphology and expression of Cys-C and β-catenin. Myocardial apoptosis in the infarcted area of different groups was evaluated by immunohistochemistry and TUNEL staining with images, the brown stained nuclei in the Sen + Ischemia + Wnt3a group showed a significant decrease compared with the Sen + Ischemia group **(A)**. The arrangements of myocardial tissues and RBC in the microcirculation of AMI lesions in different groups were shown with HE staining, the Sen + Ischemia + Wnt3a group showed significantly improved myocardial morphology compared with the Sen + Ischemia group **(B)**. Mitochondrial morphology in different groups was viewed by electron microscopy, the mitochondria in the Sen + Ischemia + Wnt3a group recovered to an oval shape **(C)**. Representative immunohistochemistry staining images of the expression of Cys-C in infarcted regions in different groups, the Sen + Ischemia + Wnt3a group showed a significant decrease in Cys-C expression compared with either of the other two ischemia groups **(D)**. Statistical analysis of myocardial apoptosis in the infarcted area of different groups, the Sen + Ischemia + Wnt3a group showed a significant decrease in the apoptotic rate of cardiomyocytes approximately 40%, compared with either of the other two ischemia groups (*p* < 0.05) **(E)**. Quantitative analysis of the expression of Cys-C and β-catenin evaluated by Western blot, the Sen + Ischemia + Wnt3a group increased the expression of β-catenin protein and decreased the expression of Cys-C compared with the Sen + Ischemia group (*p* < 0.05) **(F,G)**. Cardiomyocyte viability in different groups was assessed by CCK-8 assay, the Sen + Hypo + Wnt3a group also demonstrated growth in cell activity compared with the Sen + Hypo group (*p* < 0.05) **(H)**. The expression of Cys-C and β-catenin were evaluated by Western blot with representative images **(I)**. RBC: red blood cells; TUNEL: terminal deoxynucleotidyl transferase-mediated dUTP nick-end labeling; Ctrl: wild type mouse group; Sen: senile wild mouse group; Ctrl + Ischemia: wild type mouse AMI group; Sen + Ischemia: senile wild type mouse AMI group; Sen + Ischemia + Wnt3a: senile wild type mouse AMI group pretreated with Wnt3a 20 ng/ml. Sen + Ischemia + Wnt3a + JW-55: senile wild type mouse AMI group pretreated with Wnt3a and JW-55 ∗*p* < 0.05.

The TUNEL assay was applied to observe cardiomyocyte apoptosis (Figures 2A,E). As shown in the figures, brown stained nuclei indicate TUNEL-positive cardiomyocytes and normal nuclei were shown as blue or dark blue. The results suggested that there was no significant difference in TUNEL positive rate between the Ctrl group and the Sen group; the positive rates of both groups were lower than 10%. The Ctrl + Ischemia group demonstrated approximately 60% of TUNEL positive rate and showed a significant increase compared with either Ctrl or Sen group. Subsequent experiments revealed approximately 80% of TUNEL positive rate in the Sen + Ischemia group, indicating further increase compared with the Ctrl + Ischemia group. However, the Sen + Ischemia + Wnt3a group showed a significant decrease in the apoptotic rate of cardiomyocytes approximately 40%, compared with either of the other two ischemia groups (*p* < 0.05), but it still presented with a higher level relative to both Ctrl and Sen groups. The arrangements of myocardial tissues and red blood cells (RBCs) found in the microcirculation were observed according to HE stained around AMI lesions (Figure 2B) to identify microthrombi. It was shown that the myocardial tissues were arranged neatly in both Ctrl and Sen groups for which parachute-shaped RBCs were aligned with infrequently seen accumulation. In the Ctrl + Ischemia group, cardiomyocytes were disordered with many interstitial vacuoles and microthrombi. Compared with the Ctrl + Ischemia group, the Sen + Ischemia group had more prominent disarray of cardiomyocytes, obvious interstitial edema, and more thrombi. The Sen + Ischemia + Wnt3a group showed significantly improved myocardial morphology with a high level of microthrombi, indicating that the tissue microcirculation was severely damaged after myocardial infarction.

### Wnt3a Decreased Mitochondrial Structure and Function Disorders in Myocardial Infarction Mouse Model

Firstly, the myocardial tissues from different groups were analyzed with electron microscopy (Figure 2C). As shown in the figures, in both Ctrl and Sen groups, the mitochondria were rod-shaped, preserving visible double membrane structure and relatively complete cristae of inner membranes. In the Ctrl + Ischemia and Sen + Ischemia groups, swollen mitochondria appeared spherical, along with inner mitochondrial edema and the absence of cristae of inner membranes. Further experiments found that in the Sen + Ischemia + Wnt3a group the mitochondria recovered to an oval shape in which edema and damaged cristae were still seen.

Furthermore, myocardial tissues from different groups were immunohistochemically stained. Previous studies have shown that the excessive production of Cys-C from hypoxic ischemia models was an upstream injury signal that induced oxidative stress in cells. Therefore, our study demonstrated the difference of expression of Cys-C protein by Cys-C staining (Figure 2D). The results revealed significantly increased expression of Cys-C in the Sen, Ctrl + Ischemia, and Sen + Ischemia groups compared with the Ctrl group and increased gradually among these three groups. Further experiments revealed a significant decrease in Cys-C expression in the Sen + Ischemia + Wnt3a group compared with either of the other two ischemia groups, but it still presented with a higher level compared with the untreated Ctrl group.

### Wnt3a Increased Cardiomyocyte Viability in Senile Hypoxic Ischemia Model

We demonstrated cell viability under different model settings by CCK8 cell viability assay. There was no significant difference in cell activity between the Ctrl group and the exogenous Wnt3a intervention group, indicating no effect of Wnt3a on cell viability in normal cells. Compared with the Ctrl group, the cell activity of the Sen group was significantly reduced. Further experiments found that the Ctrl + Hypo group demonstrated a statistically significant reduction in cell activity compared with the Sen group; the Sen + Hypo group also demonstrated a statistically significant reduction in cell activity compared with the Sen group, with a statistical difference relative to the Ctrl + Hypo group (Figure 2H). The Sen + Hypo + Wnt3a group also demonstrated growth in cell activity compared with the Sen + Hypo group (*p* < 0.05). The results indicated that cell viability was not affected by pretreatment of cardiomyocytes with Wnt3a. For the Sen model and the models induced by hypoxia for 6 h, the cell viability decreased, when given the exogenous Wnt3a in the Sen + Hypo group, the cell viability increased.

### Wnt3a Decreased Cardiomyocyte Apoptosis in Senile Hypoxic Ischemia Model

**FIGURE 3:**
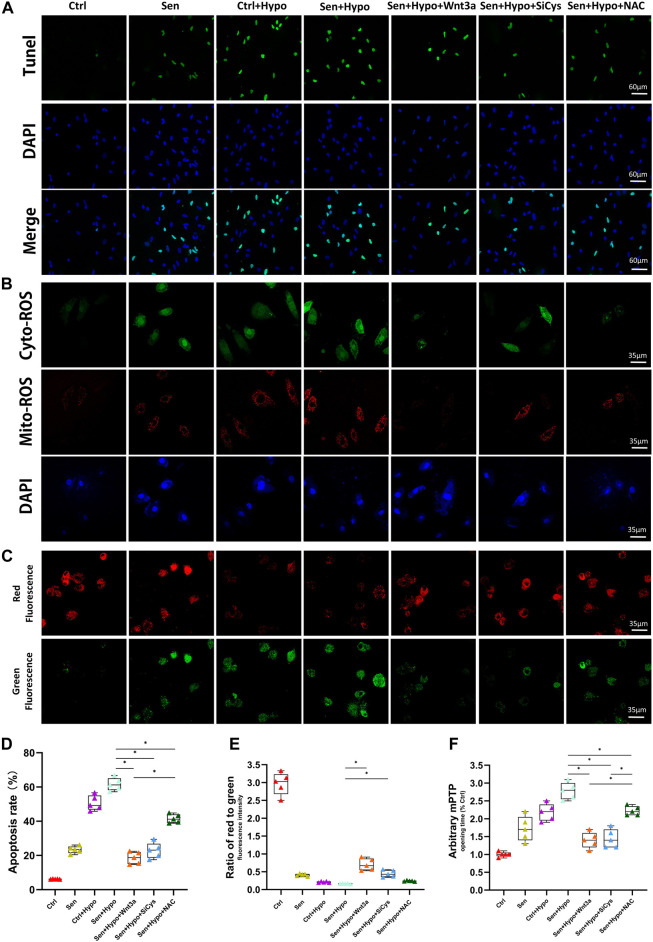
Effects of Wnt-3a, Cys-C and NAC on senile infarcted myocardium. Representative TUNEL staining (green) and DAPI staining (blue) images **(A)**. The production of ROS in cytosolic (green) and mitochondrial (red) in different groups by DCFHDA and MitoSOX assays and flow cytometry **(B)**. Representative mitochondrial membrane potential (ΔΨm) levels images **(C)**. Quantitative analysis of apoptotic cells in the peripheral infarcted areas in different groups, the apoptotic rate of exogenous treatment of Wnt 3a or NAC and knocked down Cys-C all showed a significant decrease compared with the Sen + Hypo group (*p* < 0.05) **(D)**. Quantitative analysis of mitochondrial membrane potential (ΔΨm) levels quantitative analysis **(E)** and mitochondrial permeability transition pore (mPTP) opening time quantitative analysis **(F)** in different groups, the Sen + Hypo group significantly accounted for prolonged mPTP opening time and decreased ΔΨm (ratio of red to green), while Wnt3a, siCys-C, and additional NAC reversed mitochondria pathological state to some extent. Ctrl: wild type cardiomyocyte group; Sen: senile wild type cardiomyocyte group; Ctrl + Hypo:wild hypoxia type cardiomyocyte group; Sen + Hypo: senile wild hypoxia type cardiomyocyte group; Sen + Hypo + Wnt3a: senile wild hypoxia type cardiomyocyte group pretreated with Wnt3a 20 ng/ml; Sen + Hypo + SiCys: senile wild hypoxia type cardiomyocyte group knocked down Cys protein; Sen + Hypo + NAC: senile wild hypoxia type cardiomyocyte group pretreated with cytosolic ROS scavenger.*p < 0.05.

The number and distribution of apoptotic cells peripheral to myocardial infarction were measured using TUNEL assay. The results showed that the Ctrl group had the lowest apoptotic rate (about 6%), and the senile cardiomyocyte group had an apoptotic rate of 23%. Under hypoxia conditions, both normal cardiomyocytes (approximately 51%) and senile cardiomyocytes (approximately 62%) demonstrated significantly increased apoptotic rates. Pretreatment with Wnt3a 20 ng/ml 2 h before hypoxia led to a significant reduction of apoptotic rate for senile cardiomyocytes, which was 19%, in further experiments, we knocked down Cys-C using siRNA technology to observe changes in apoptotic rates in different groups. It suggested that Cys-C knock-down performed 48 h before hypoxia led to a significant reduction of apoptotic rates for senile cardiomyocytes, which was 23%. Senile cardiomyocytes showed a significant decrease in apoptotic rate after the addition of the antioxidant NAC 6 h before hypoxia, which was 41%. (Figures 3A,D). The results indicated that both senile factor and hypoxia induction accelerated the apoptosis of cardiomyocytes, and the occurrence of apoptosis could be controlled by exogenous treatment with Wnt3a or Cys-C knock-down since the hypoxic-ischemic injury may be mediated by the Wnt/Cys-C signaling pathway. Exogenous treatment of NAC can similarly reduce the rate of apoptosis, but the effect is weaker than that of the Wnt3a group, possibly because NAC can only block the cytoplasmic ROS pathway and does not play a blocking role in the mitochondrial ROS.

### Wnt/β-Catenin/Cystatin C Mediated ROS Burst Resulting in Mitochondrial Oxidative Stress Injury

Based on the results from our experiments, we hypothesized that there existed a Wnt/β-catenin/Cys-C pathway-mediated ROS burst, which induced apoptosis increase. Therefore, we extracted total cell protein to examine the differences in expression of pathway proteins across groups using WB assay.

Indicating significantly higher Cys-C expression in the Sen group and the Sen + Hypo group compared with the normal group. Pretreatment with Wnt3a increased the expression of β-catenin and decreased the expression of Cys-C protein compared with the Sen + Hypo group. Pretreatment with β-catenin blocker JW-55 increased the expression of Cys-C and decreased the expression of β-catenin protein compared with the Sen + Hypo + Wnt3a group (*p*＜0.05). (Figures 2F,G,I).

Previous studies have shown that increased Cys-C can aggravate cellular oxidative stress and mediate increased cellular reactive oxygen species. According to our preliminary studies, a high ROS level in the cytoplasm may cause mitochondrial oxidative stress damage. Therefore, we observed immunofluorescence by confocal microscopy to co-localize cytoplasmic ROS (green) and mitochondrial ROS (red). The results suggested that the green and red ROS showed enhanced fluorescence intensity for cardiomyocytes after hypoxia induction.

Wnt3a was given to activate this pathway, resulting in reduced fluorescence intensity of cytosolic green ROS and mitochondrial red ROS. Using siRNA technology to knock down the expression of Cys-C microscopic results showed that the fluorescence intensity of both cytosolic green ROS and mitochondrial red ROS was reduced. With the addition of cytosolic ROS scavenger NAC, the red ROS fluorescence intensity did not weaken. (Figure 3B).

In addition, to confirm whether excessive ROS leads to dysfunction of mitochondria, we measured the mPTP opening time and mitochondrial membrane potential (ΔΨm) among different groups. (Figures 3C,E,F). Notably, the Sen + Hypo group significantly accounted for prolonged mPTP opening time and decreased ΔΨm (ratio of red to green), while Wnt3a, siCys-C, and additional NAC reversed mitochondria pathological state to some extent.

## Discussion

**FIGURE 4:**
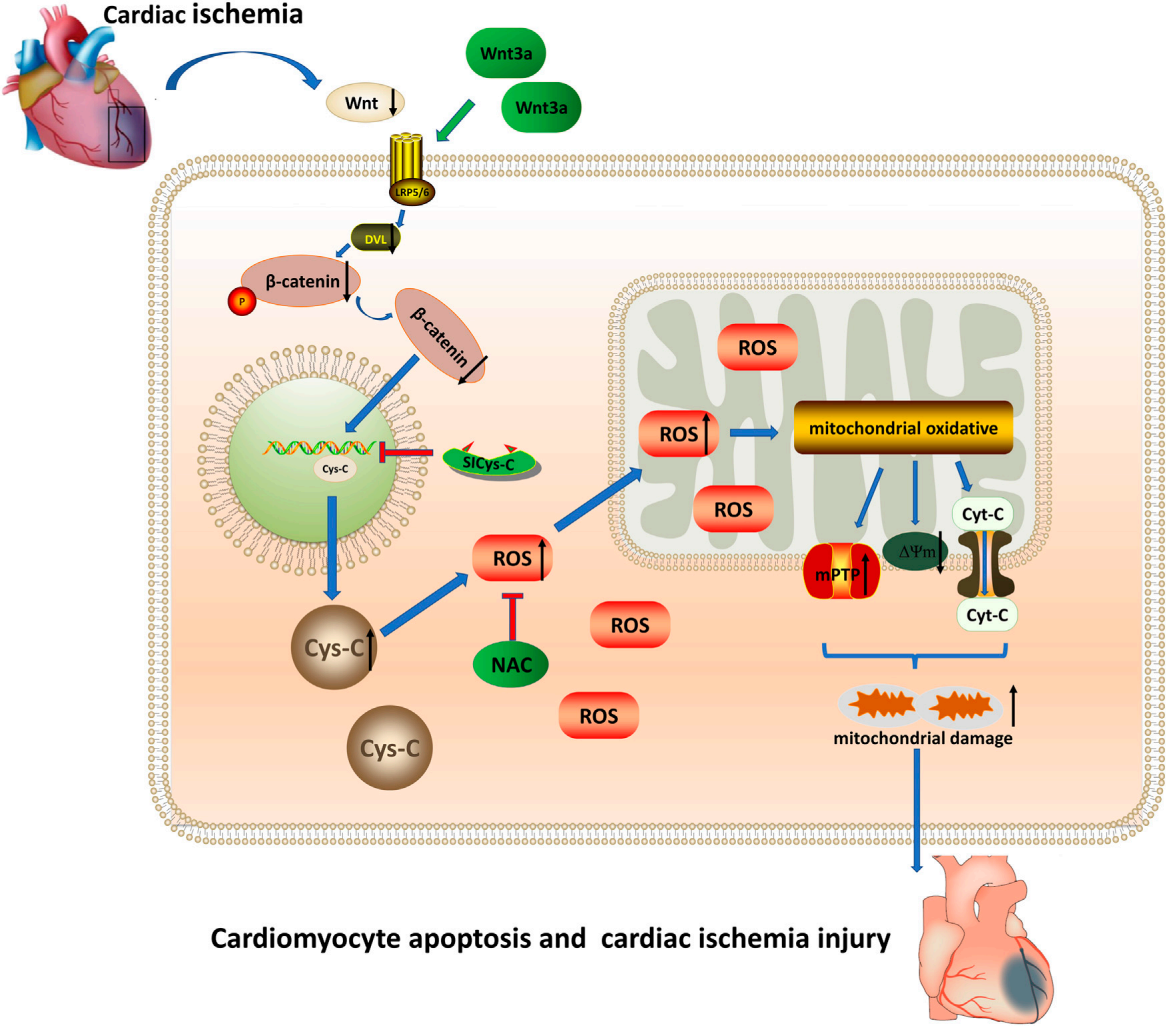
Wnt 3a/β-catenin reduce the expression of cytoplasmic Cys-C through transnuclear action, and reduce the occurrence of mitochondrial oxidative stress injury induced *via* Cys-C/ROS, inhibited Cys-C/ROS-induced cardiomyocyte apoptosis and cardiac injury. Cys-C, Cystatin C; ROS, reactive oxygen species; mPTP, mitochondrial permeability transition pore; Cyt-C, mitochondrial apoptotic protein; ΔΨm, mitochondrial membrane potential; LRP5/6 Frizzled–LRP5/6 receptor; Dvl, dishevelled protein; NAC, cytosolic ROS scavenger; Si-CysC, siRNA technology to knock down the expression of Cys-C.

In this study, through the elderly ischemic model *in-vivo* and vitro experiments, our results confirmed that the addition of exogenous Wnt3a to elderly mice could effectively reduce the myocardial infarct area, improve heart microcirculation, and reduce the apoptotic cardiomyocytes in the infarcted myocardium, and improved the structure and function of infarcted mice. The results showed that Wnt/β-catenin could reduce the expression of cytoplasmic Cys-C through transnuclear action, and subsequently reduce the occurrence of mitochondrial oxidative stress damage induced by Cys-C-ROS. The addition of exogenous Wnt3a and blocking Cys-C expression could inhibit mitochondrial oxidative stress damage and relieve the acute myocardial ischemic damage, especially in the elderly ischemic model (Figure 4).

Our previous clinical studies have found that the expression level of Cys-C in elderly patients (≥80 years) with the acute coronary syndrome (ACS) is significantly higher than that in elderly non-ACS patients and ACS patients aged 60–70 years. The clinical research theory confirmed that Cys-C is an independent risk factor for death and long-term MACE events in elderly ACS patients (Fu et al., 2013; Fu et al., 2018). However, the specific molecular mechanism of Cys-C leading to complex disease, severe myocardial damage, and poor prognosis in elderly ACS patients is still unclear. Studies have shown that Cys-C can increase the production of reactive oxygen species (ROS) and cause oxidative stress in the cytoplasm, thus aggravating myocardial damage, Wnt/β-catenin/ROS play a big role in oxidative stress injury, leading to increased apoptosis and dysfunction of myocardial cells (Shen et al., 2014; Shen et al., 2018) and Wnt/β-catenin activation has also been proven to reduce myocardial ischemia injury in other studies (Wo et al., 2016; Yang et al., 2019).

Some studies indicated that elderly people showed dysfunction of Wnt/β-catenin expression, and this phenomenon was especially exhibited in Wnt3a-mediated survival which changed with age (Brown et al., 2019). Recently, there is a study showing that activating the Wnt/β-catenin could also effectively reduce ischemia-reperfusion injury in the elderly rat brain (Li et al., 2019). Similarly, indicated that melanin could alleviate hypoxic-ischemic damage through the Wnt/β -catenin pathway in senescence HUVECs cells Wang et al. (2019). Cui et al. further confirmed that ischemia-reperfusion damage of the heart could increase the apoptosis of senescence myocardial cells by inhibiting the expression of the Wnt3a/β-catenin pathway, but the specific mechanism needs further exploration. The mechanism of dysfunction of Wnt3a/β-catenin might be closely related to mitochondrial division and apoptosis (Zhao et al., 2018). Based on the studies before, we hypothesized that the transnuclear action of β-catenin might promote the expression of protective proteins to decrease mito-apoptosis. Therefore, in our study, we focused on whether Wnt/β-catenin and subsequently Cys-C/ROS increase experienced interactions which participate in the occurrence and development of myocardial ischemic damage.

Our animal and cellular experiments showed that exogenous Wnt3a effectively reduced the expression of Cys-C and consequently the cytoplasmic ROS level, indicating that the activation of Wnt/β-catenin may affect the expression of Cys-C. We speculated that this could be explained by the nuclear translocation of β-catenin, which reduced the transcription level of Cys-C to protect cells against oxidative stress damage. Furthermore, our data confirmed that the senescence group showed significantly increased Cys-C and ROS levels, which indicated that the Cys-C/ROS pathway is not only involved in ischemic heart disease but also further aggressively in aging cells. Previous studies have found that the nuclear translocation of β-catenin is closely related to cell growth under physiological conditions (Fernández-Sánchez et al., 2015; Griffin et al., 2018). Recent research revealed that Wnt/β-catenin may affect the transcription and expression of c-Myc proteins *via* the nuclear translocation of β-catenin when myocardial injuries occur, including dilated cardiomyopathy and ischemic cardiomyopathy, and participate in myocardial remodeling under abnormal conditions (Hou et al., 2016; Piven and Winata, 2017). Our present study is consistent with studies before that demonstrated that Wnt/β-catenin, as an upstream signaling molecule, is involved in the molecular mechanism of cardiac remodeling under physiological and pathological conditions *via* its nuclear translocation and direct protective effects.

Molecular approaches including gene silencing and pathway inhibition were applied in this study to further understand and verify the upstream and downstream position of each protein in the Wnt pathway. In order to exclude the effect of Wnt3a pretreatment on the viability of cardiomyocytes, CCK8 assay was used to determine cell viability and no significant difference in cell viability was observed between the Ctrl group and the Wnt3a group.

It was demonstrated in this experiment that excessive Cys-C-induced ROS production elicited oxidative stress in cells, which in turn induced excess ROS in the cytoplasm. Subcellular localization of ROS was further determined *via* co-localization of fluorescence staining of mitochondria and cytoplasm. We found that cytosolic ROS freely passed through the mitochondrial bilayer membranes and accumulated in the inner mitochondria. The ROS accumulated in the mitochondria further oxidized important proteins related to the mitochondrial respiratory chain, which led to the breakage of the chain, the opening of mPTP, the decrease of mitochondrial membrane potential, the leakage of mitochondrial apoptotic protein Cyt-C to the cytoplasm, and ultimately activation of the mitochondrial apoptotic pathway (Zhang et al., 2016; Zhou et al., 2018). After treatment with exogenous Wnt3a, we found that the Wnt/β-catenin pathway was activated and subsequently both cytoplasmic and mitochondrial ROS were reduced. Tunel assay also suggested that the apoptosis rate in the injury group was minimized. Therefore, those results confirmed that Wnt/β-catenin is the upstream signal pathway protecting cells against oxidative stress injury.

In conclusion, the present study confirmed that Cys-C induced acute myocardial infarction and myocardial injury *via* the ROS/mitochondrial signaling pathway, especially in cardiomyocytes of aging mice. Wnt/β-catenin could inhibit the subsequent expression of Cys-C and effectively reduces myocardial injury of acute myocardial infarction. This explored mechanism provided new evidence and targets for the clinical treatment for this disease.

## Data Availability

The original contributions presented in the study are included in the article/Supplementary Materials, further inquiries can be directed to the corresponding authors.
