# Supplementary figures and images for: Wnt 3a Protects Myocardial Injury in Elderly Acute Myocardial Infarction by Inhibiting Serum Cystatin C/ROS-Induced Mitochondrial Damage (part 1 of 2)
Source: Front Physiol. 2022 Jul 22;13:950960. doi: 10.3389/fphys.2022.950960 (PMC9355253; doi:10.3389/fphys.2022.950960)

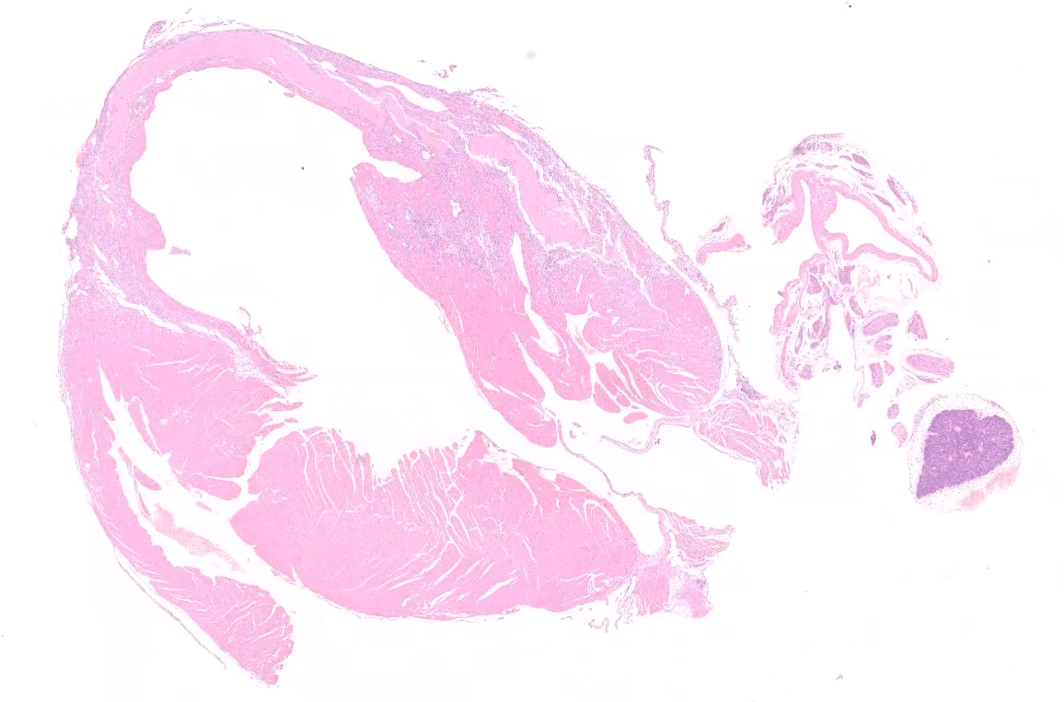

Supplement: Supplementary file 1 [file DataSheet3.ZIP › Original source data 3/FIGURE1.A HE staining/HE.Ctrl+Ischemia.jpg]

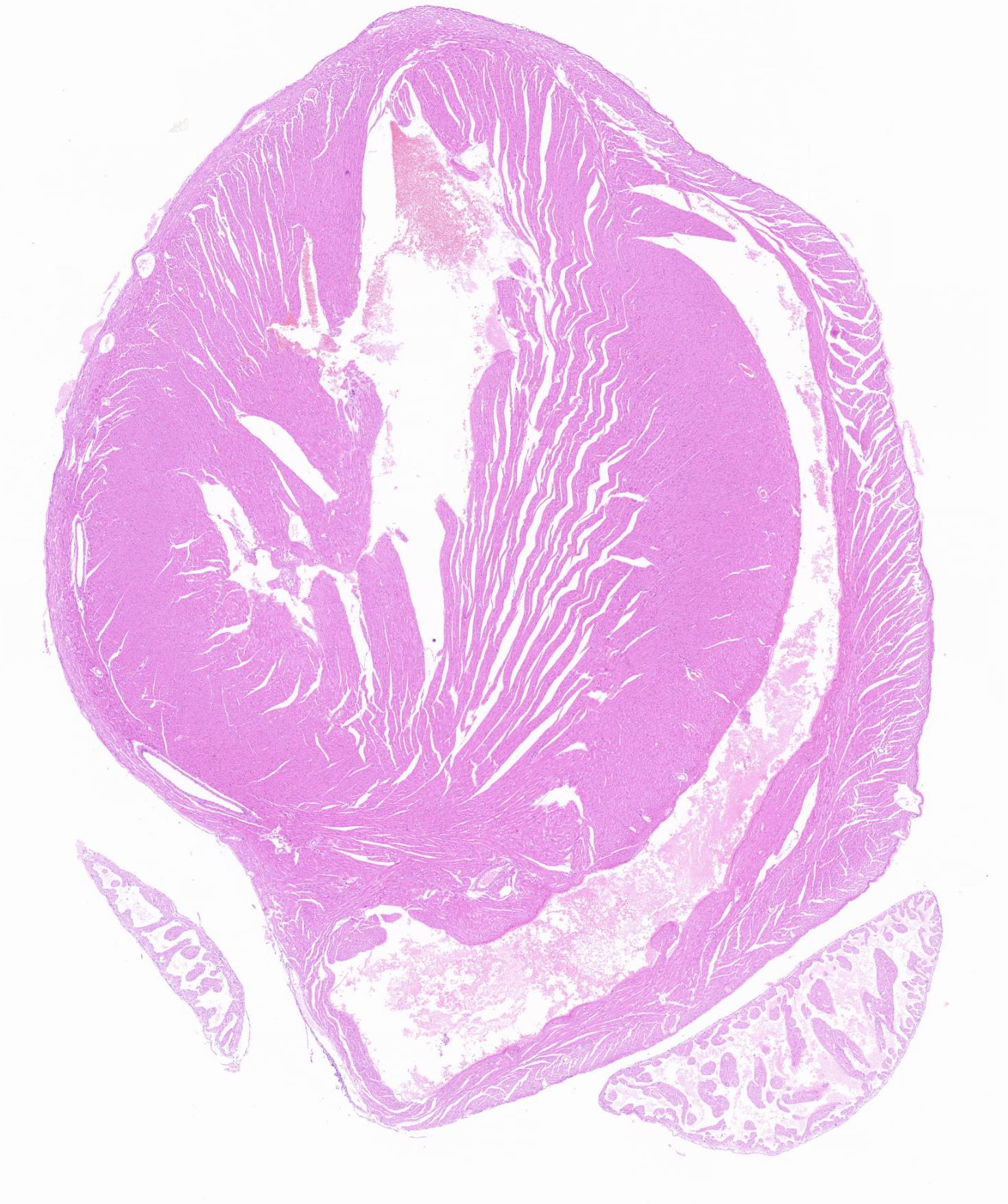

Supplement: Supplementary file 1 [file DataSheet3.ZIP › Original source data 3/FIGURE1.A HE staining/HE.Ctrl.jpg]

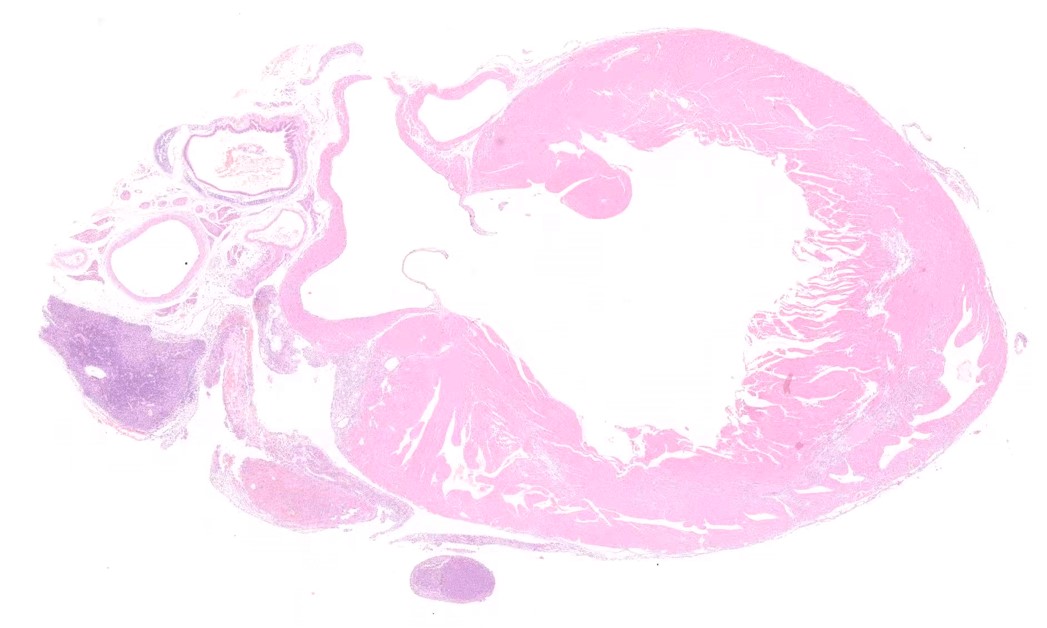

Supplement: Supplementary file 1 [file DataSheet3.ZIP › Original source data 3/FIGURE1.A HE staining/HE.Sen+Ischemia+Wnt3a.jpg]

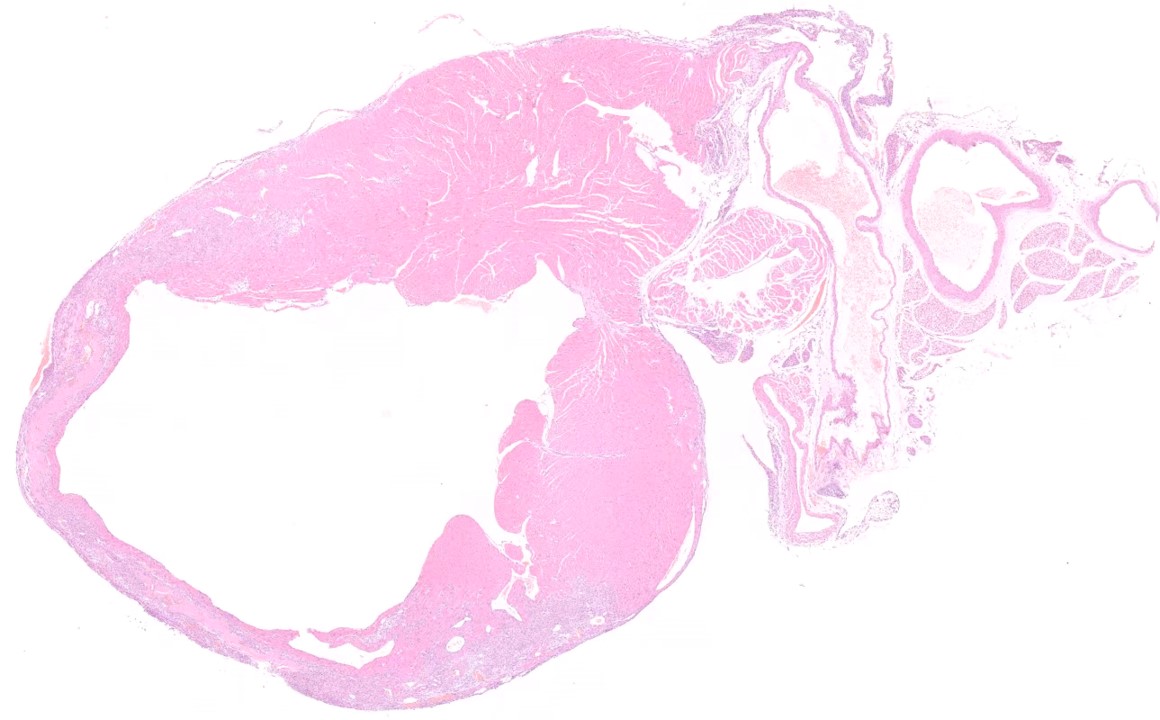

Supplement: Supplementary file 1 [file DataSheet3.ZIP › Original source data 3/FIGURE1.A HE staining/HE.Sen+Ischemia.jpg]

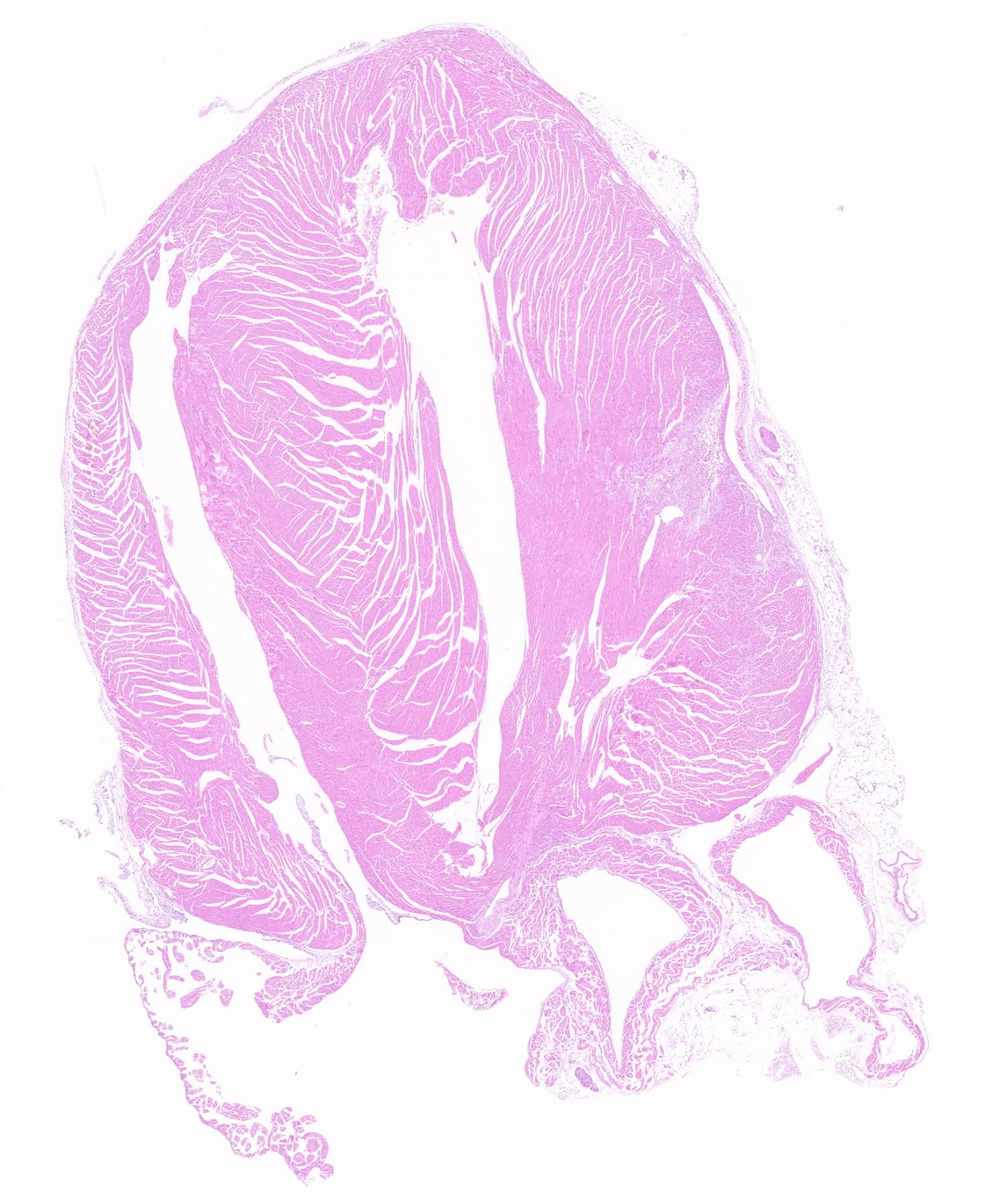

Supplement: Supplementary file 1 [file DataSheet3.ZIP › Original source data 3/FIGURE1.A HE staining/HE.Sen.jpg]

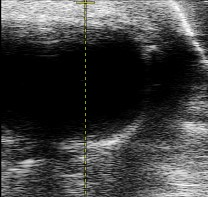

Supplement: Supplementary file 1 [file DataSheet3.ZIP › Original source data 3/FIGURE1.B Echocardiograpy/Ctrl+Ischemia.jpg]

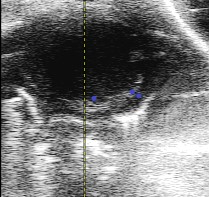

Supplement: Supplementary file 1 [file DataSheet3.ZIP › Original source data 3/FIGURE1.B Echocardiograpy/Ctrl.jpg]

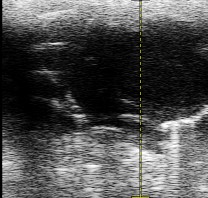

Supplement: Supplementary file 1 [file DataSheet3.ZIP › Original source data 3/FIGURE1.B Echocardiograpy/Sen+Ischemia+Wnt3a.jpg]

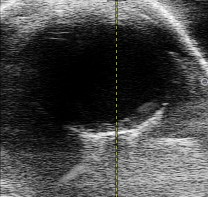

Supplement: Supplementary file 1 [file DataSheet3.ZIP › Original source data 3/FIGURE1.B Echocardiograpy/Sen+Ischemia.jpg]

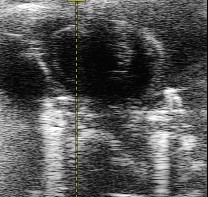

Supplement: Supplementary file 1 [file DataSheet3.ZIP › Original source data 3/FIGURE1.B Echocardiograpy/Sen.jpg]

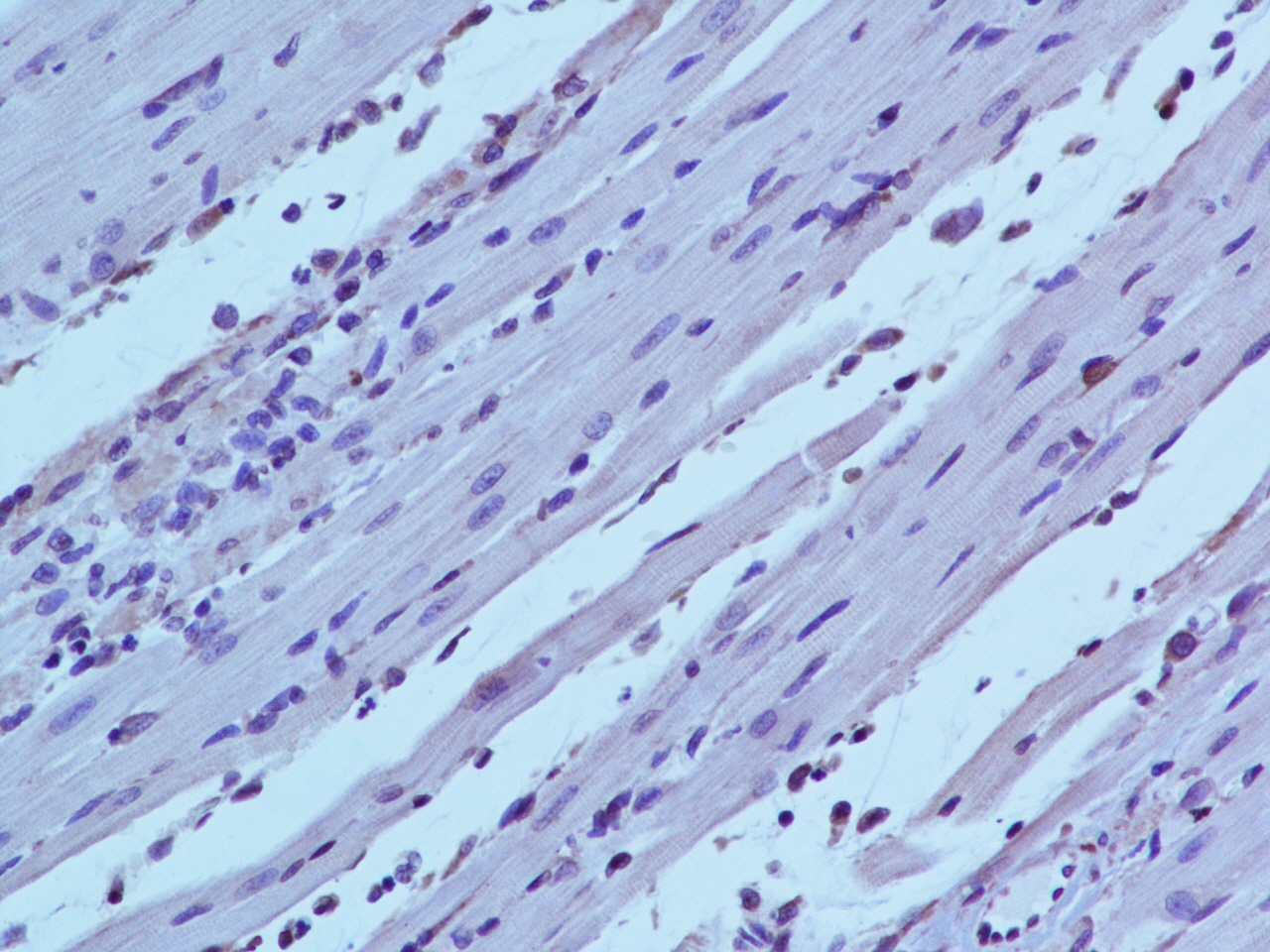

Supplement: Supplementary file 1 [file DataSheet3.ZIP › Original source data 3/FIGURE2.A TUNEL staining/Ctrl+Ischemia.jpg]

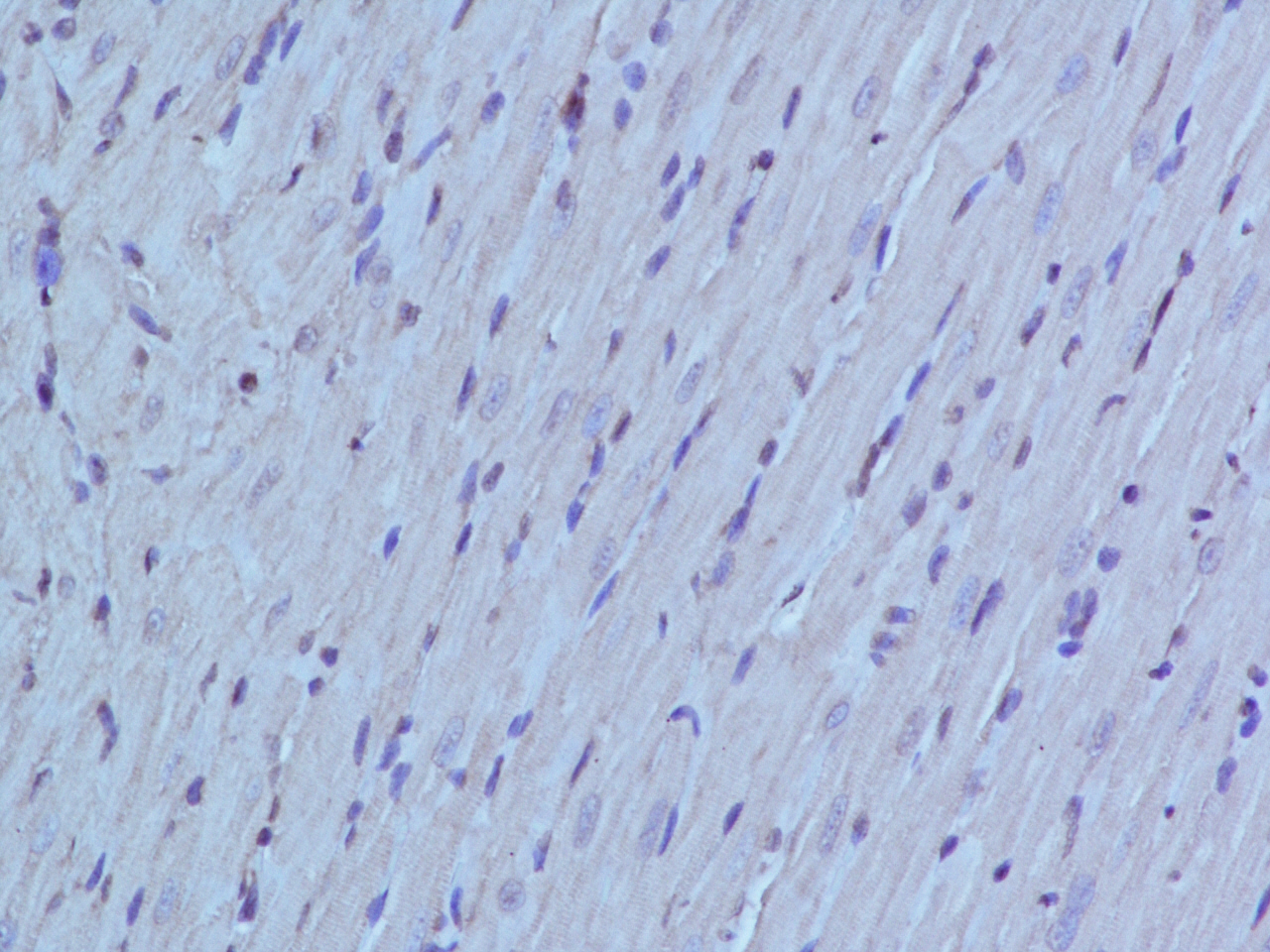

Supplement: Supplementary file 1 [file DataSheet3.ZIP › Original source data 3/FIGURE2.A TUNEL staining/Ctrl.jpg]

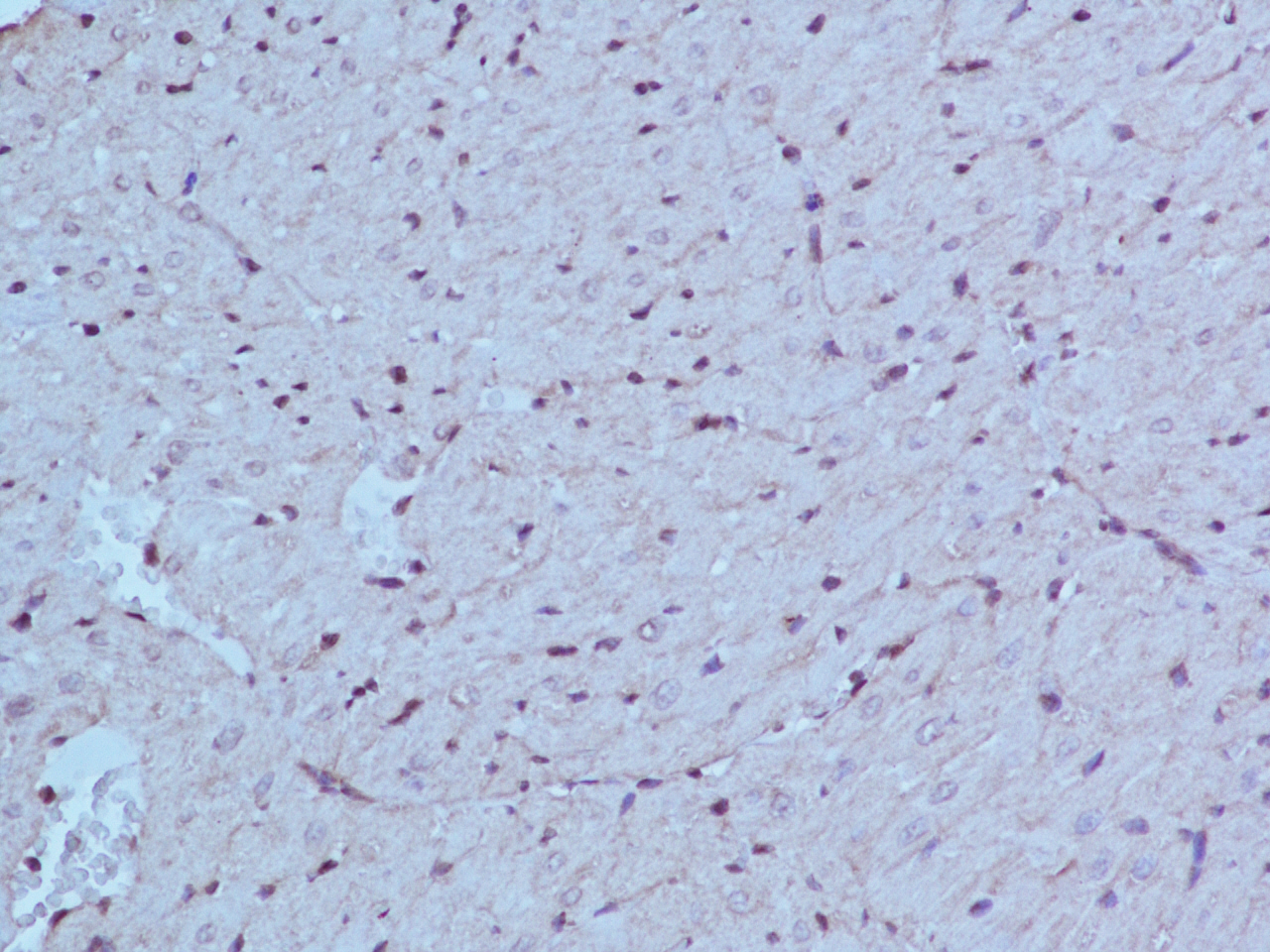

Supplement: Supplementary file 1 [file DataSheet3.ZIP › Original source data 3/FIGURE2.A TUNEL staining/Sen+Ischemia+Wnt3a.jpg]

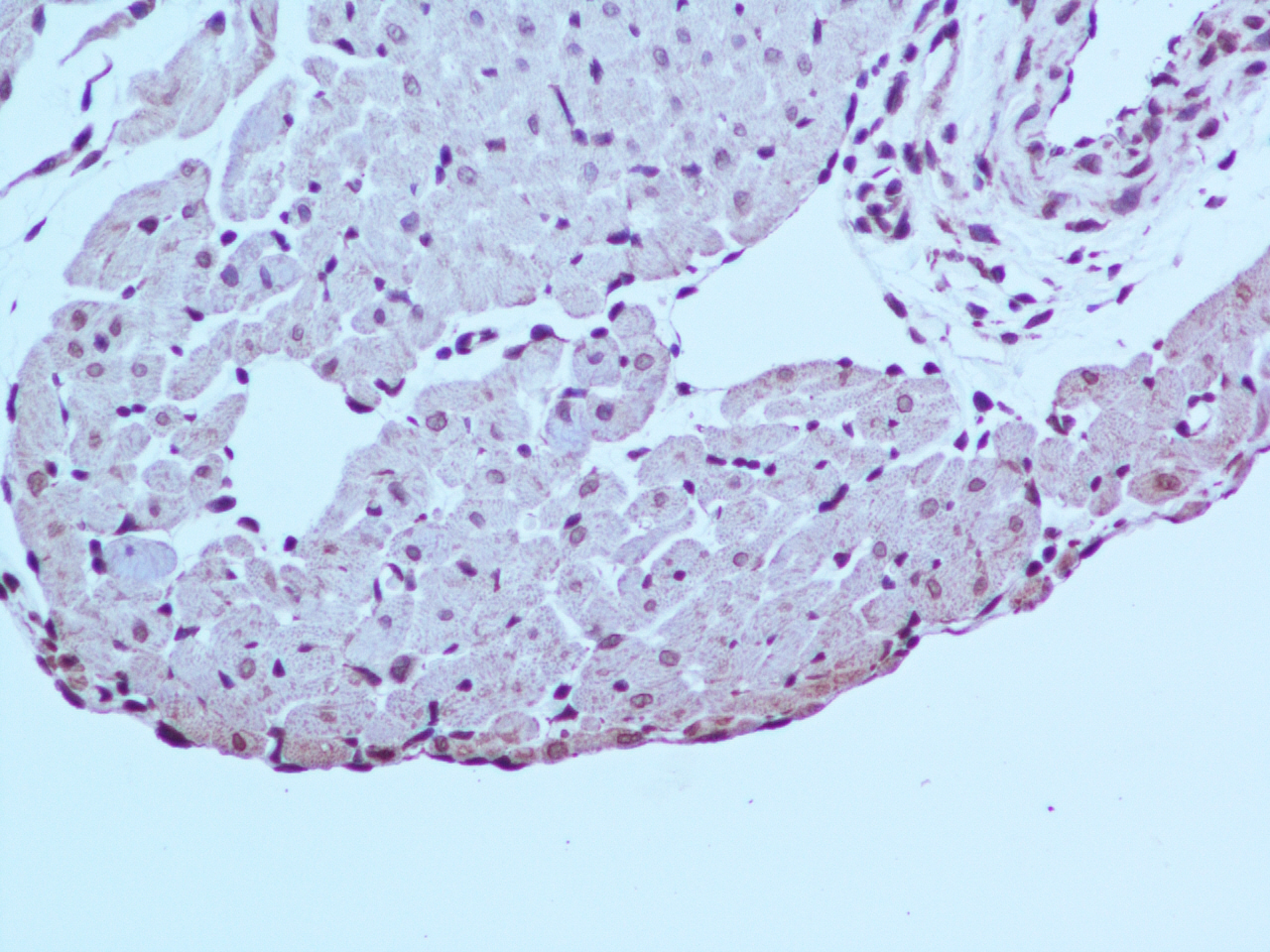

Supplement: Supplementary file 1 [file DataSheet3.ZIP › Original source data 3/FIGURE2.A TUNEL staining/Sen+Ischemia.jpg]

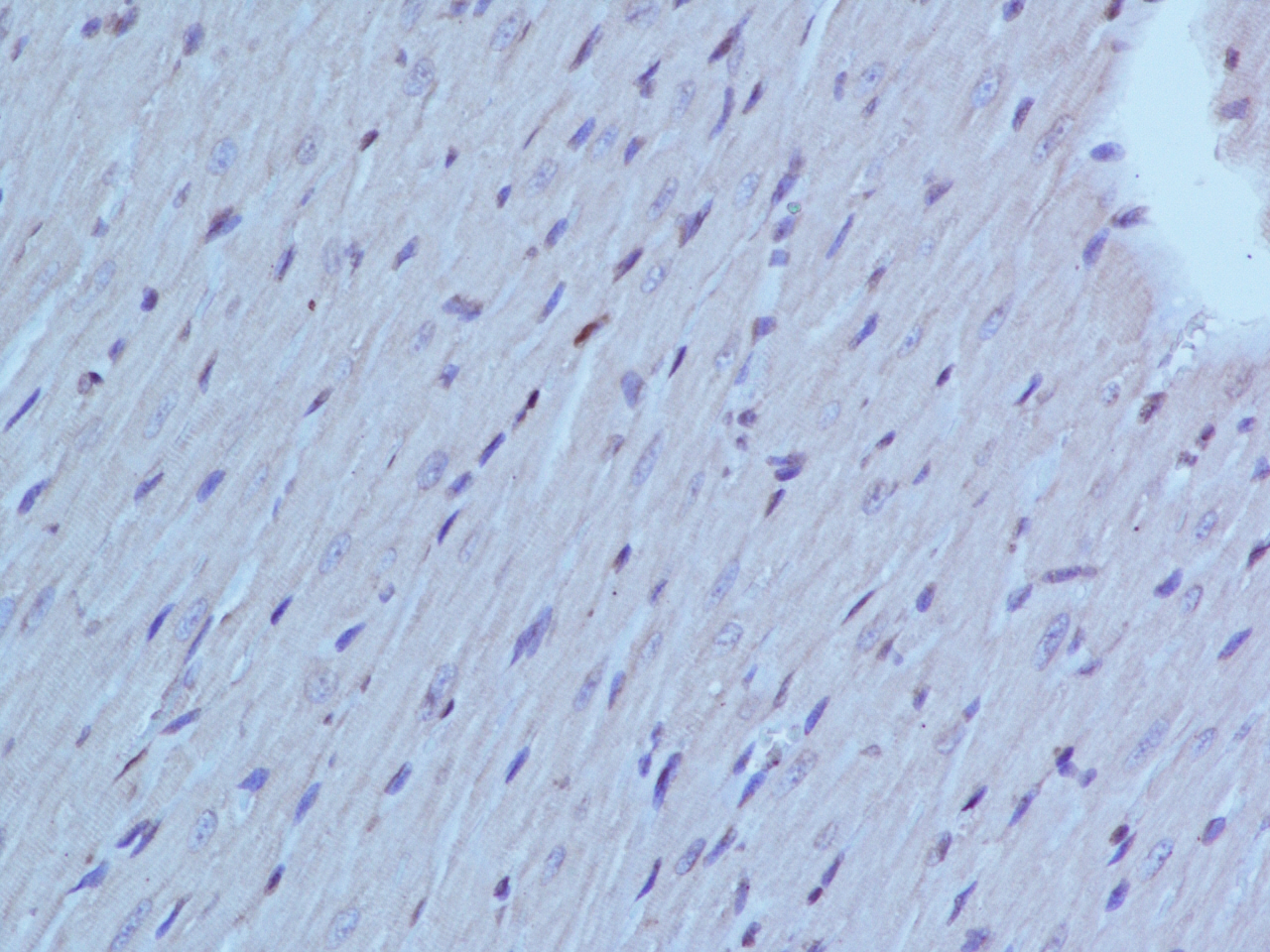

Supplement: Supplementary file 1 [file DataSheet3.ZIP › Original source data 3/FIGURE2.A TUNEL staining/Sen.jpg]

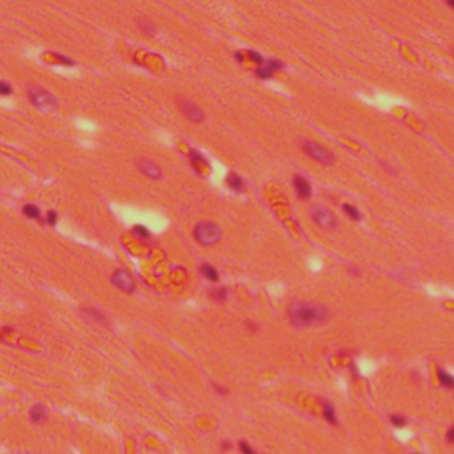

Supplement: Supplementary file 1 [file DataSheet3.ZIP › Original source data 3/FIGURE2.B RBC/Ctrl+Ischemia.tif]

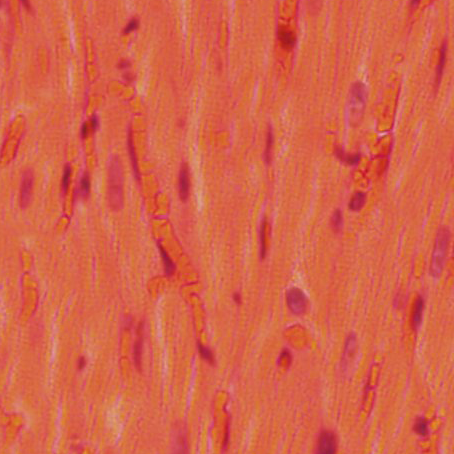

Supplement: Supplementary file 1 [file DataSheet3.ZIP › Original source data 3/FIGURE2.B RBC/Ctrl.tif]

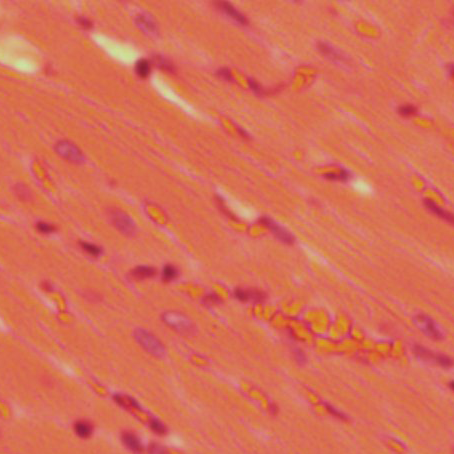

Supplement: Supplementary file 1 [file DataSheet3.ZIP › Original source data 3/FIGURE2.B RBC/Sen+Ischemia+Wnt3a.tif]

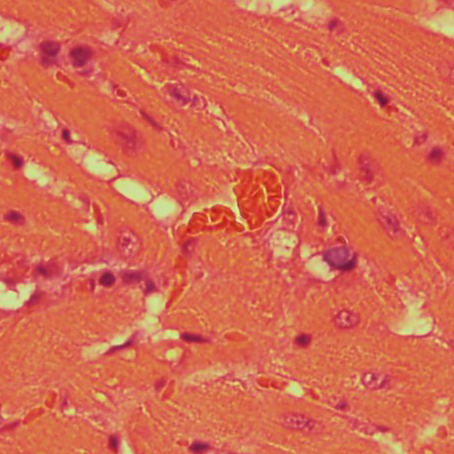

Supplement: Supplementary file 1 [file DataSheet3.ZIP › Original source data 3/FIGURE2.B RBC/Sen+Ischemia.tif]

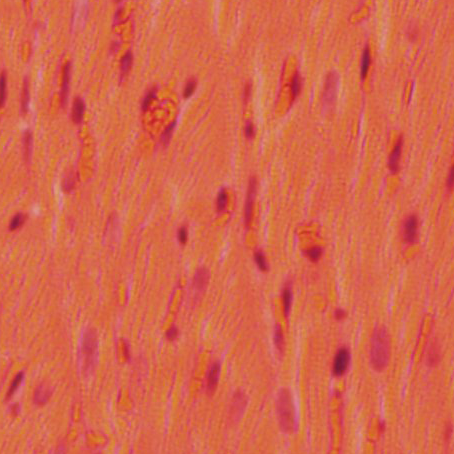

Supplement: Supplementary file 1 [file DataSheet3.ZIP › Original source data 3/FIGURE2.B RBC/Sen.tif]

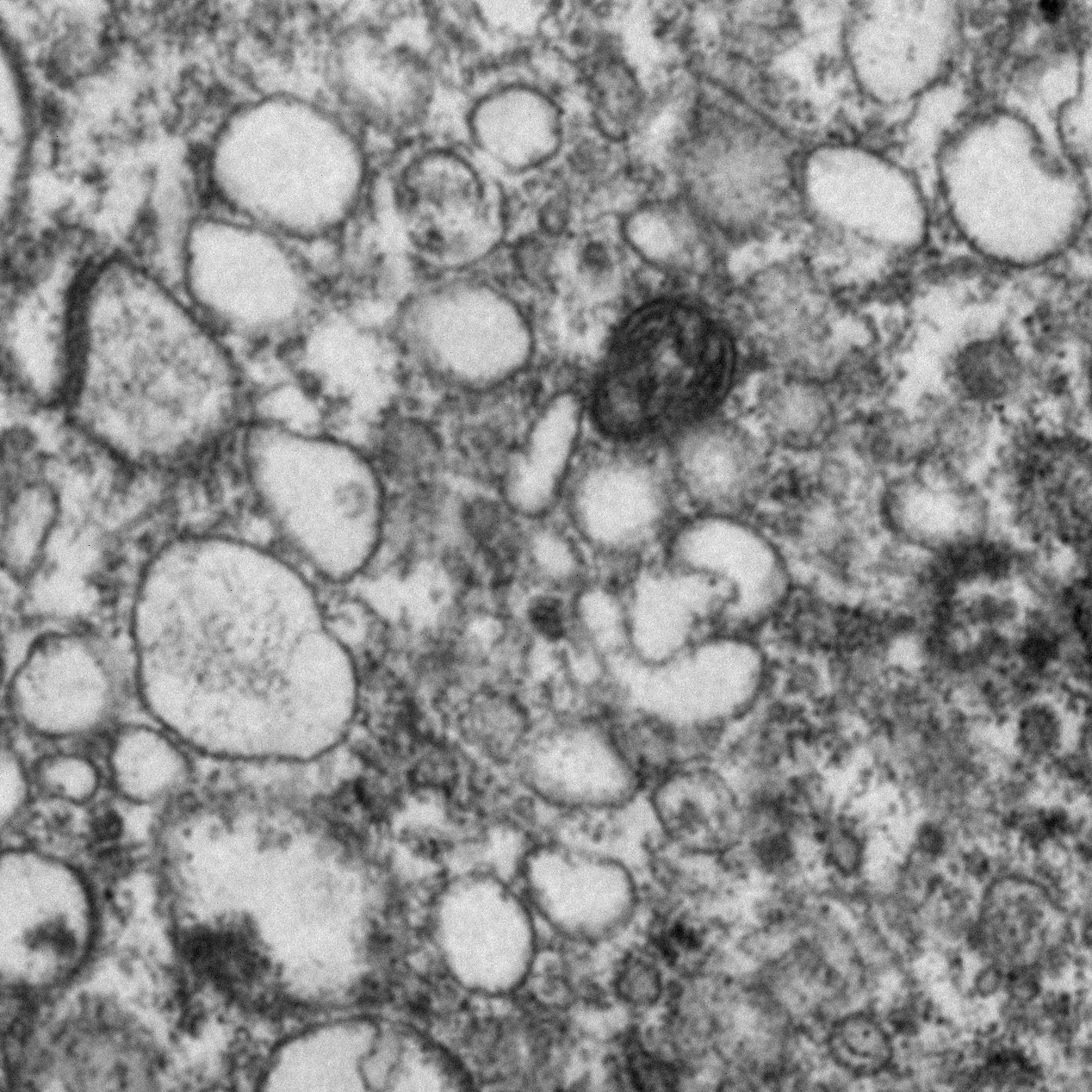

Supplement: Supplementary file 1 [file DataSheet3.ZIP › Original source data 3/FIGURE2.C Microscope/Ctrl+Ischemia.tif]

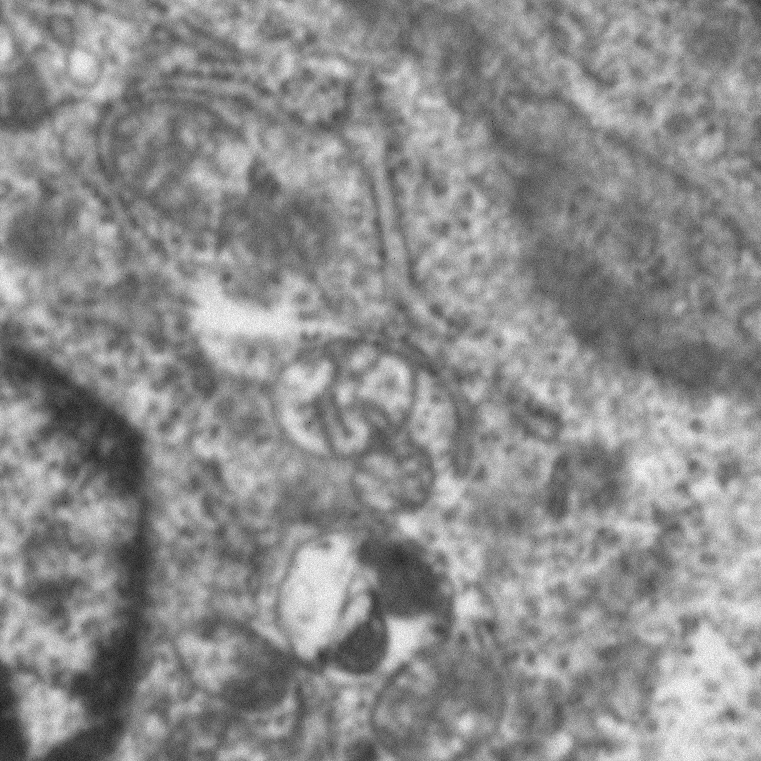

Supplement: Supplementary file 1 [file DataSheet3.ZIP › Original source data 3/FIGURE2.C Microscope/Ctrl.tif]

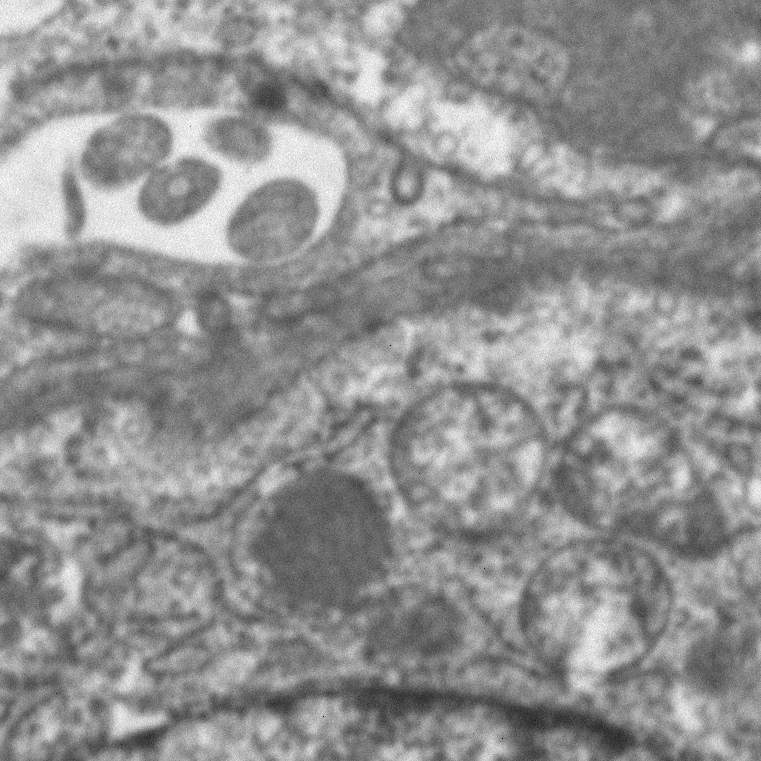

Supplement: Supplementary file 1 [file DataSheet3.ZIP › Original source data 3/FIGURE2.C Microscope/Sen+Ischemia+Wnt3a.tif]

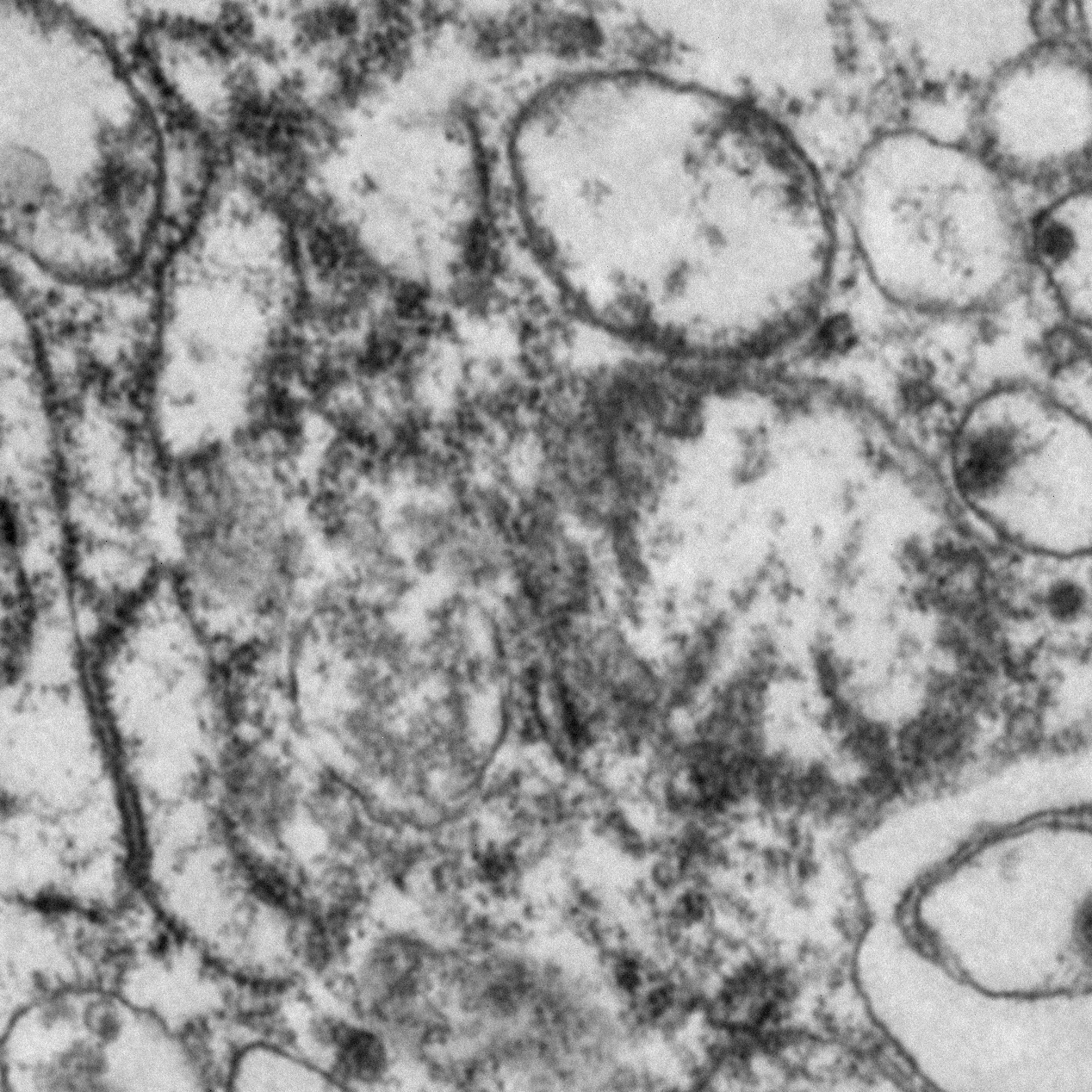

Supplement: Supplementary file 1 [file DataSheet3.ZIP › Original source data 3/FIGURE2.C Microscope/Sen+Ischemia.tif]

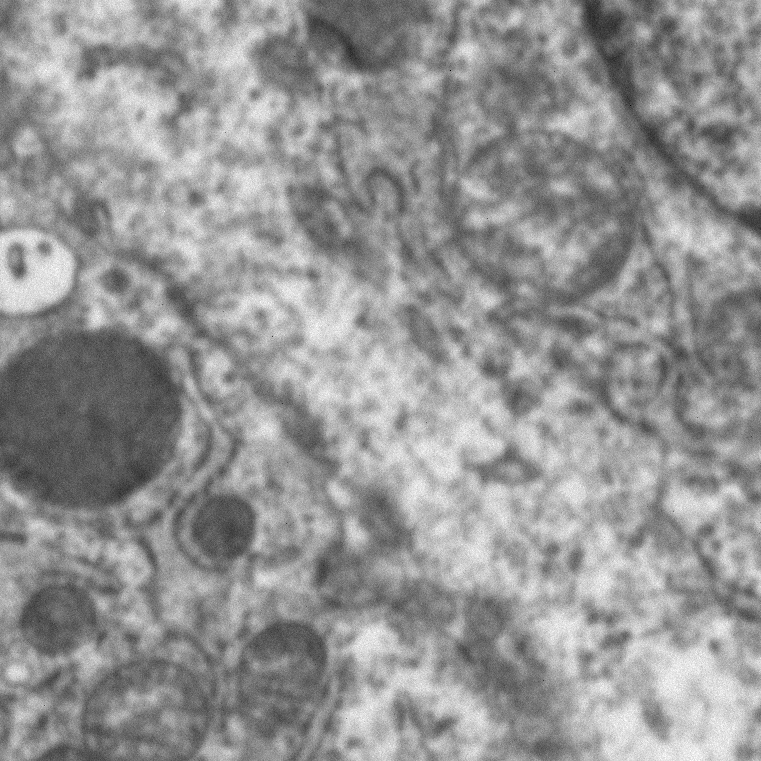

Supplement: Supplementary file 1 [file DataSheet3.ZIP › Original source data 3/FIGURE2.C Microscope/Sen.tif]

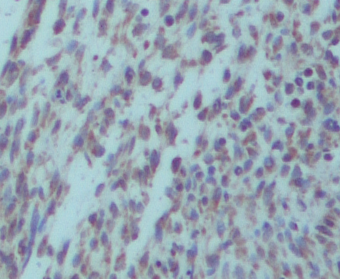

Supplement: Supplementary file 1 [file DataSheet3.ZIP › Original source data 3/FIGURE2.D Cys-c/Ctrl+Ischemia.tif]

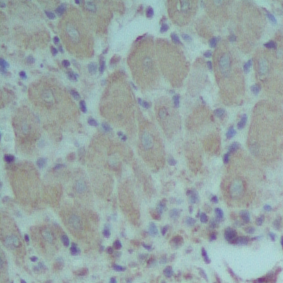

Supplement: Supplementary file 1 [file DataSheet3.ZIP › Original source data 3/FIGURE2.D Cys-c/Ctrl.tif]

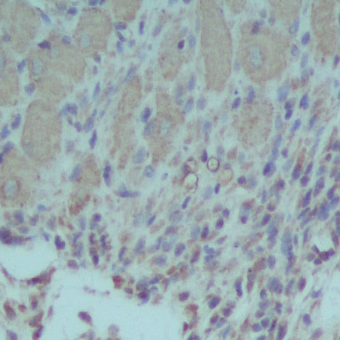

Supplement: Supplementary file 1 [file DataSheet3.ZIP › Original source data 3/FIGURE2.D Cys-c/Sen+Ischemia+Wnt3a.tif]

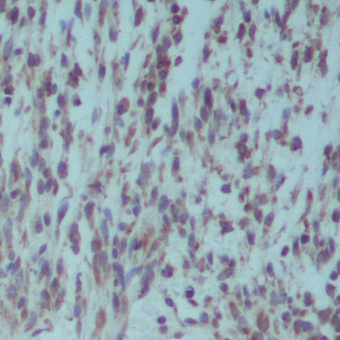

Supplement: Supplementary file 1 [file DataSheet3.ZIP › Original source data 3/FIGURE2.D Cys-c/Sen+Ischemia.tif]

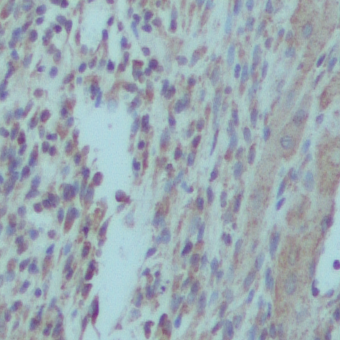

Supplement: Supplementary file 1 [file DataSheet3.ZIP › Original source data 3/FIGURE2.D Cys-c/Sen.tif]

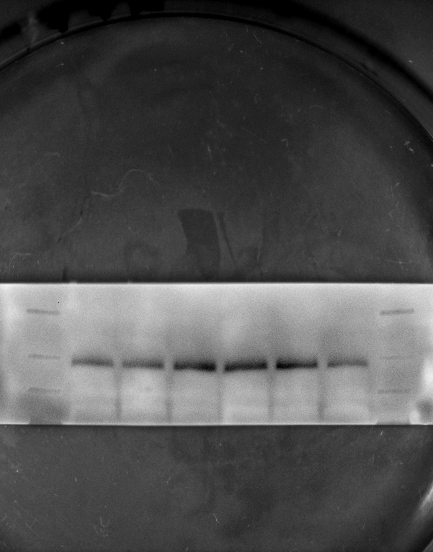

Supplement: Supplementary file 1 [file DataSheet3.ZIP › Original source data 3/FIGURE2.I Western bolt/cys-c.tif]

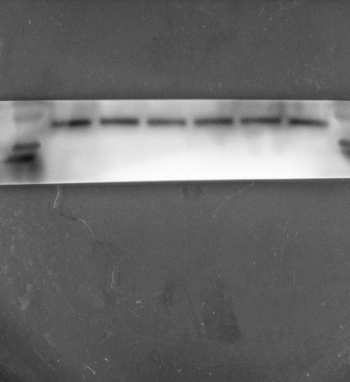

Supplement: Supplementary file 1 [file DataSheet3.ZIP › Original source data 3/FIGURE2.I Western bolt/α-actin.tif]

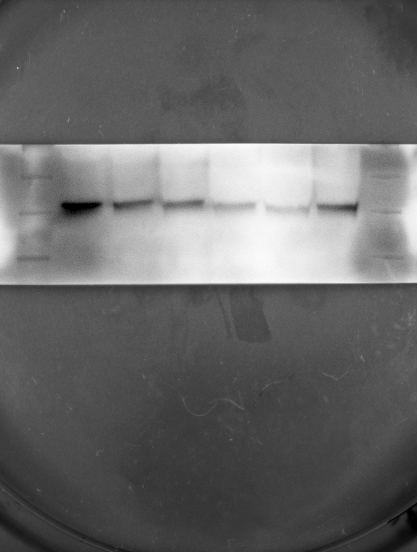

Supplement: Supplementary file 1 [file DataSheet3.ZIP › Original source data 3/FIGURE2.I Western bolt/β-catenin.tif]

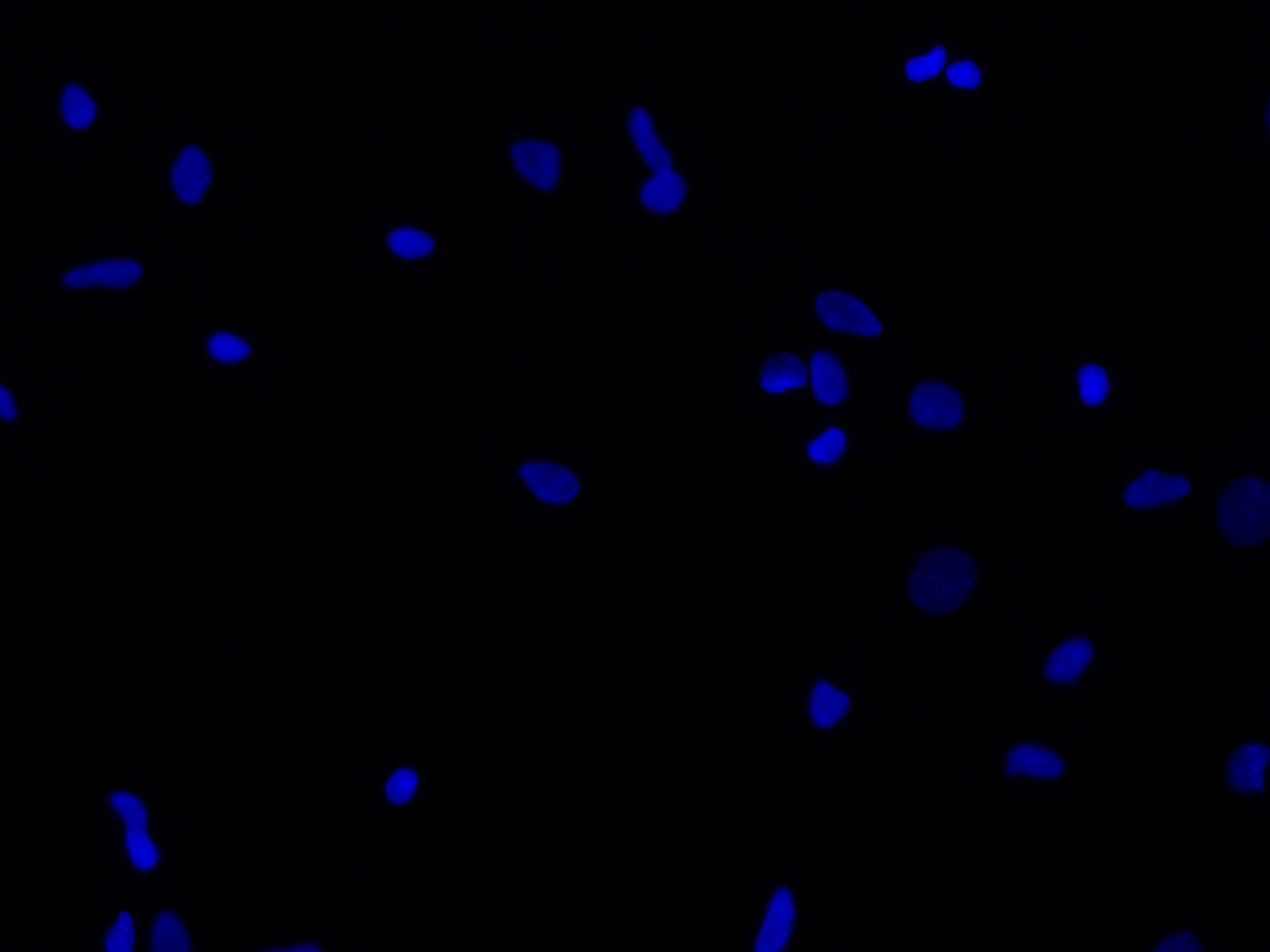

Supplement: Supplementary file 2 [file DataSheet4.ZIP › Original source data 4/FIGURE3.A TUNEL-DAPI-MERGE/Ctrl/dapi.jpg]

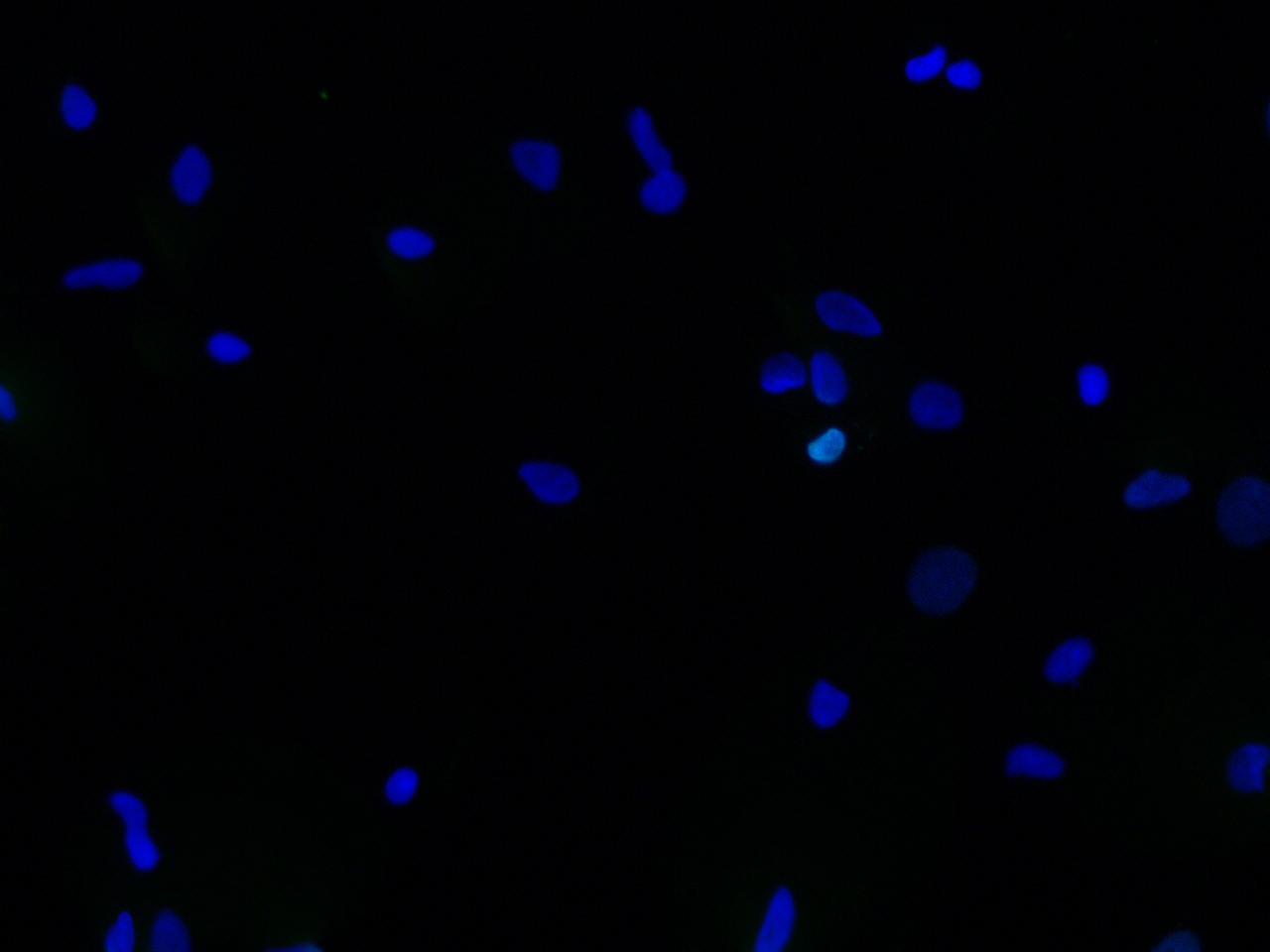

Supplement: Supplementary file 2 [file DataSheet4.ZIP › Original source data 4/FIGURE3.A TUNEL-DAPI-MERGE/Ctrl/merge.tif]

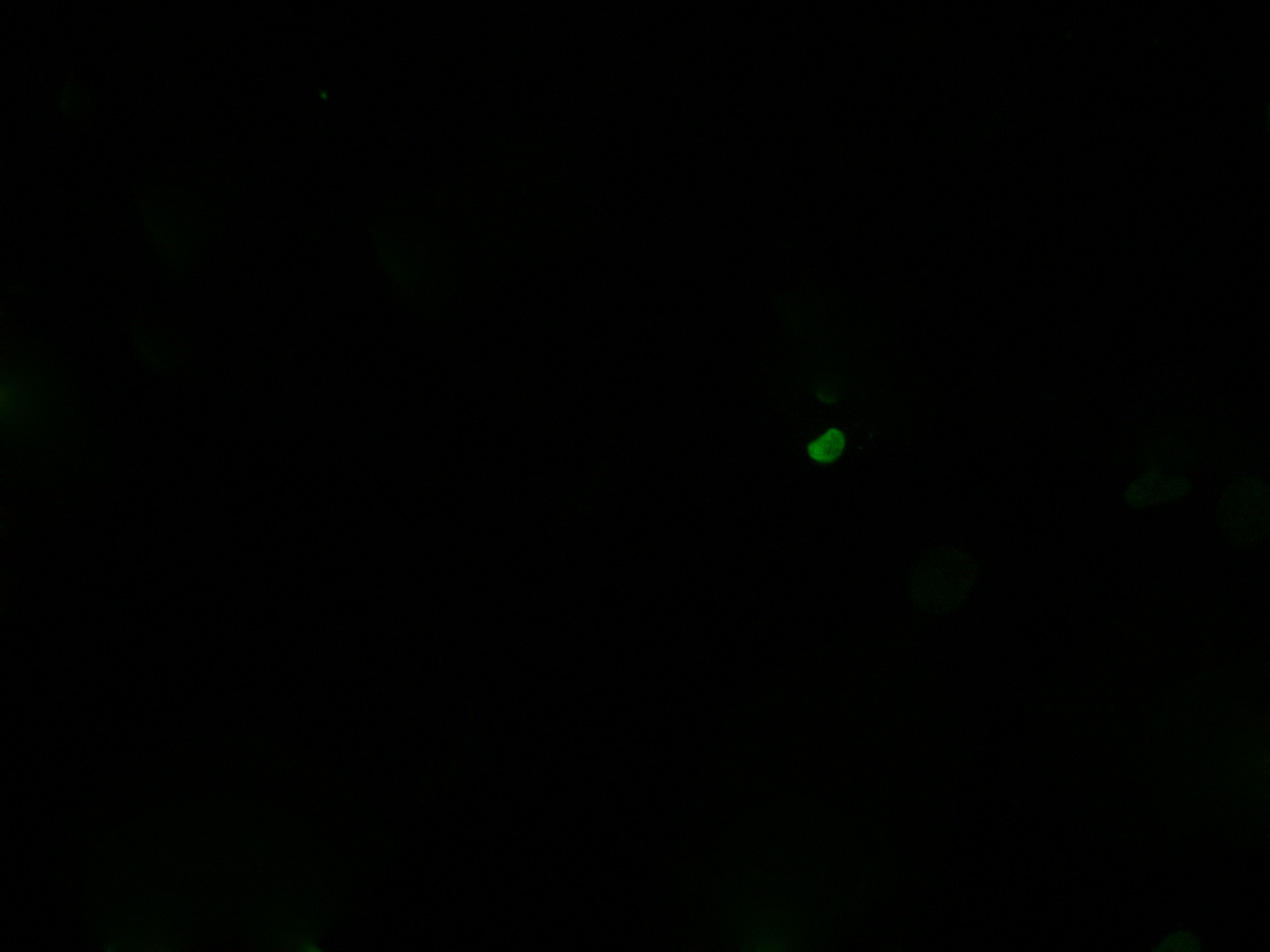

Supplement: Supplementary file 2 [file DataSheet4.ZIP › Original source data 4/FIGURE3.A TUNEL-DAPI-MERGE/Ctrl/tunel.tif]

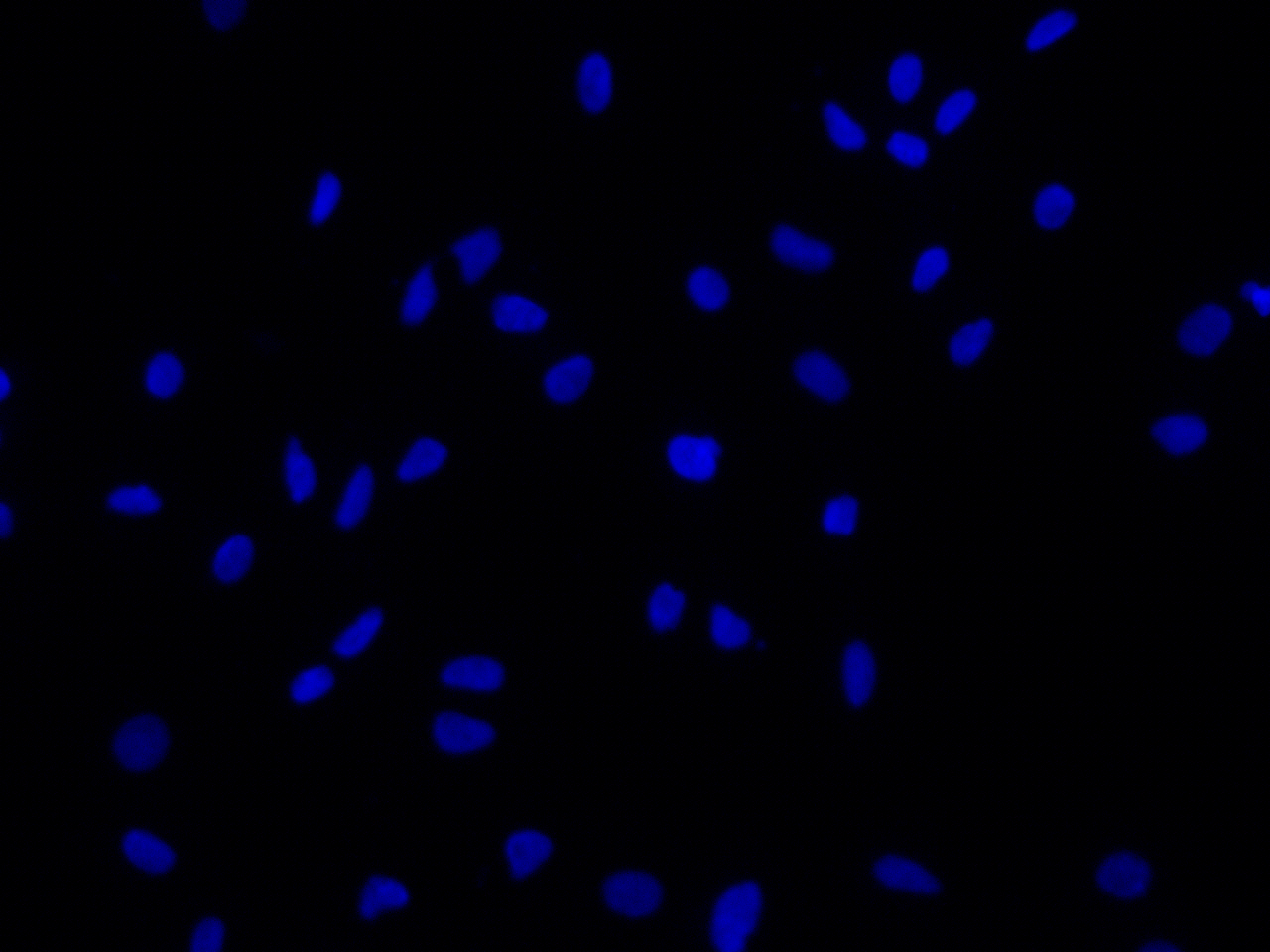

Supplement: Supplementary file 2 [file DataSheet4.ZIP › Original source data 4/FIGURE3.A TUNEL-DAPI-MERGE/Ctrl+Ischemia/dapi.tif]

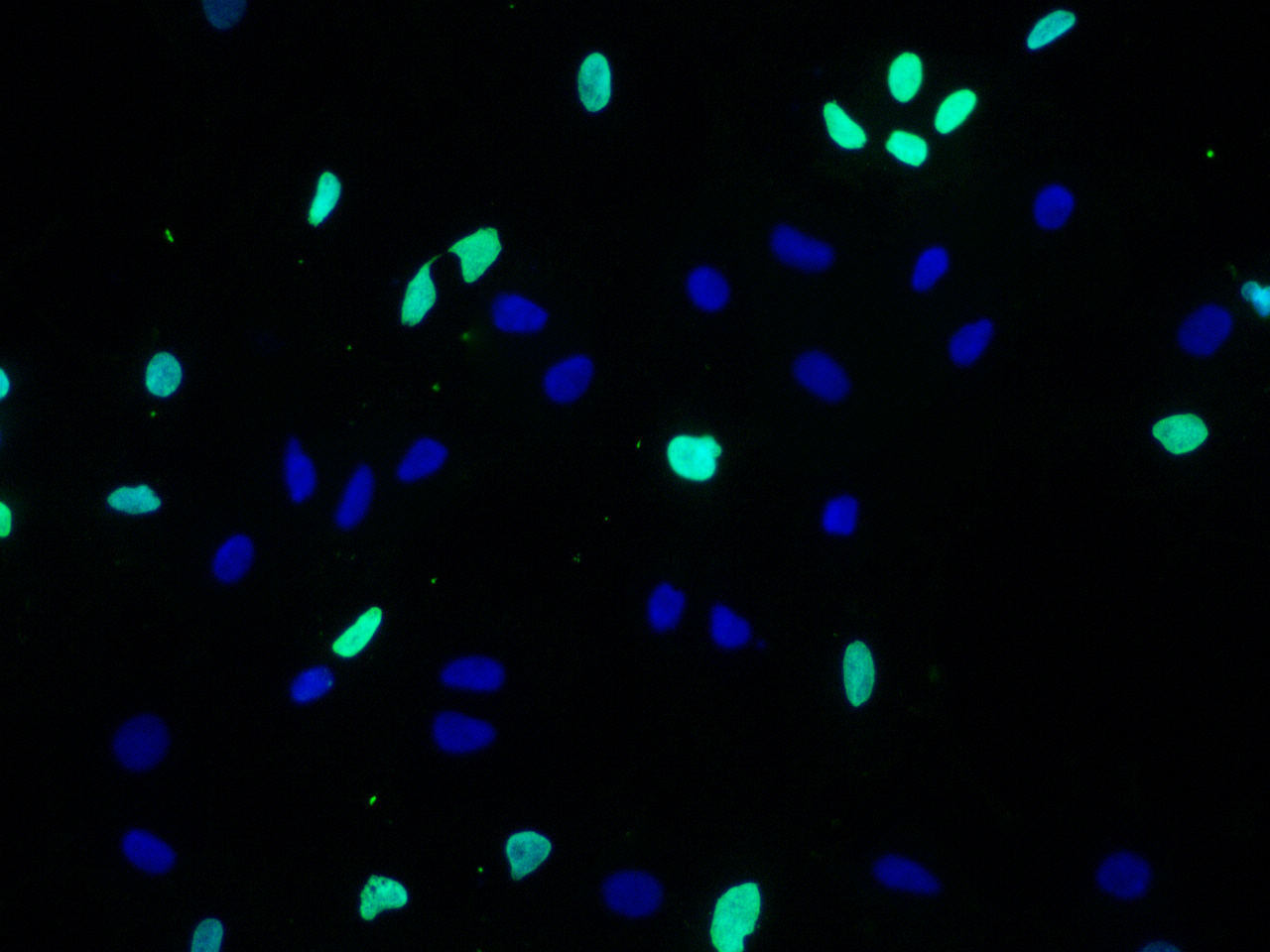

Supplement: Supplementary file 2 [file DataSheet4.ZIP › Original source data 4/FIGURE3.A TUNEL-DAPI-MERGE/Ctrl+Ischemia/merge.tif]

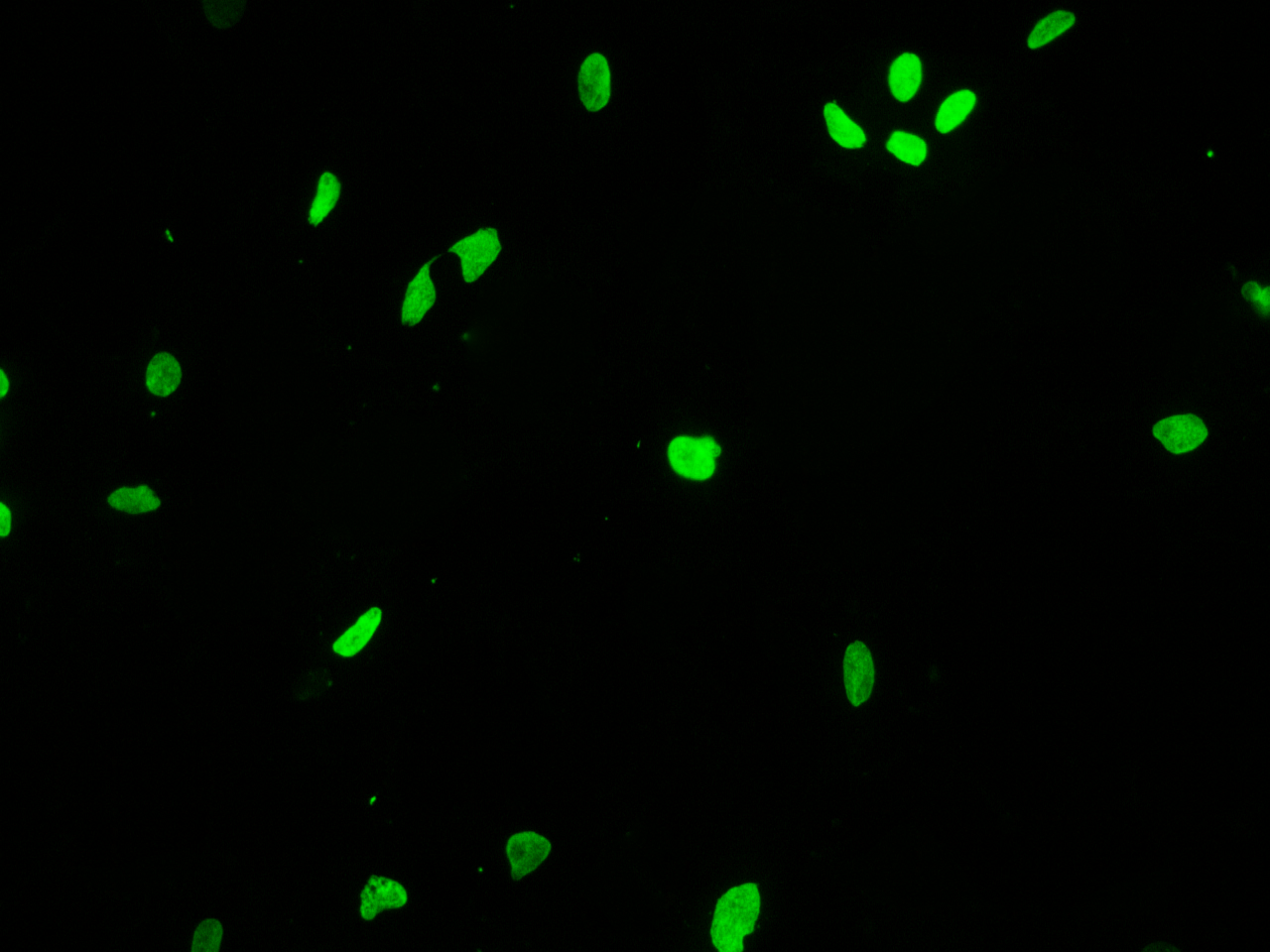

Supplement: Supplementary file 2 [file DataSheet4.ZIP › Original source data 4/FIGURE3.A TUNEL-DAPI-MERGE/Ctrl+Ischemia/tunel.tif]

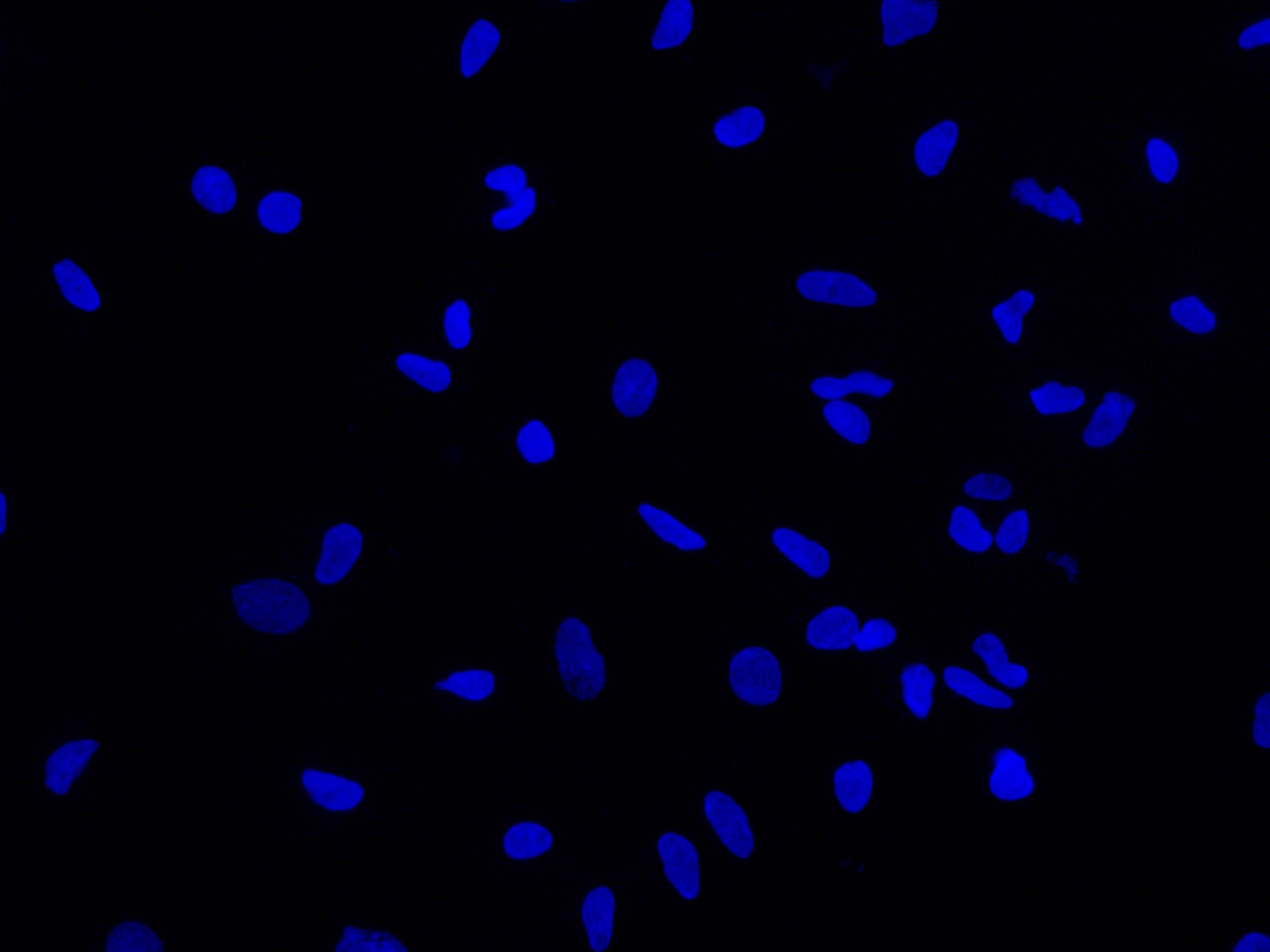

Supplement: Supplementary file 2 [file DataSheet4.ZIP › Original source data 4/FIGURE3.A TUNEL-DAPI-MERGE/Sen/dapi.tif]

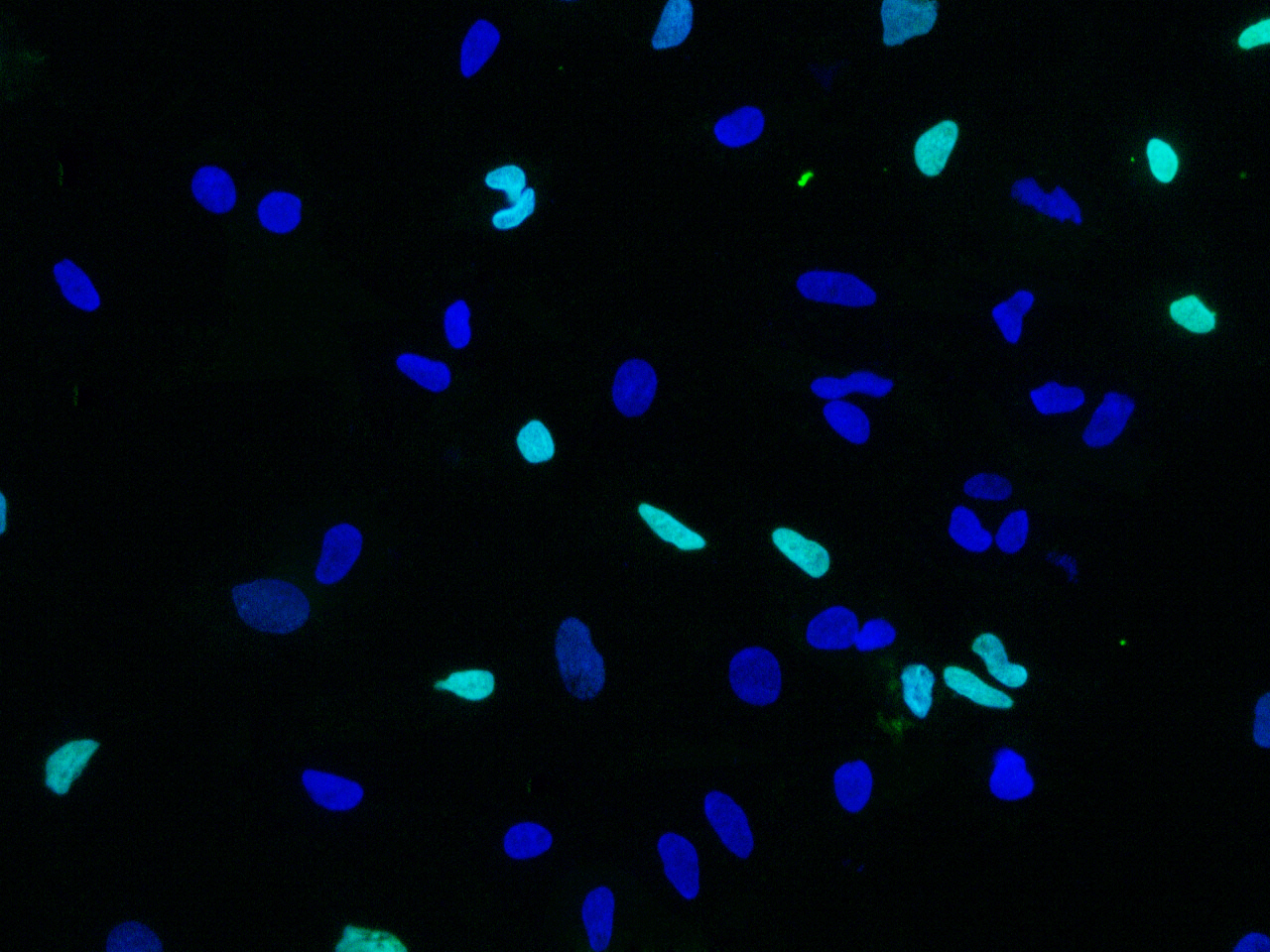

Supplement: Supplementary file 2 [file DataSheet4.ZIP › Original source data 4/FIGURE3.A TUNEL-DAPI-MERGE/Sen/merge.tif]

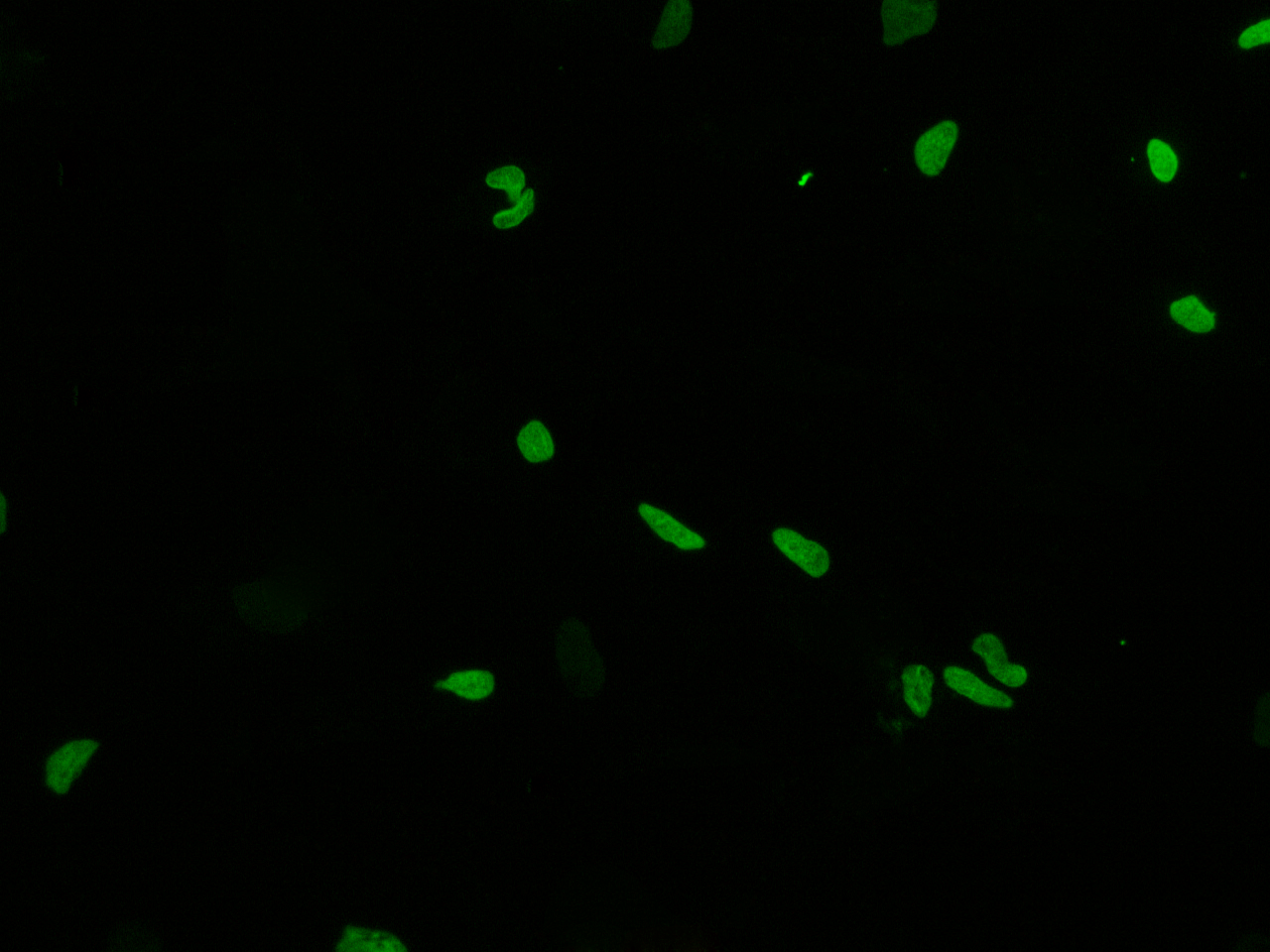

Supplement: Supplementary file 2 [file DataSheet4.ZIP › Original source data 4/FIGURE3.A TUNEL-DAPI-MERGE/Sen/tunel.tif]

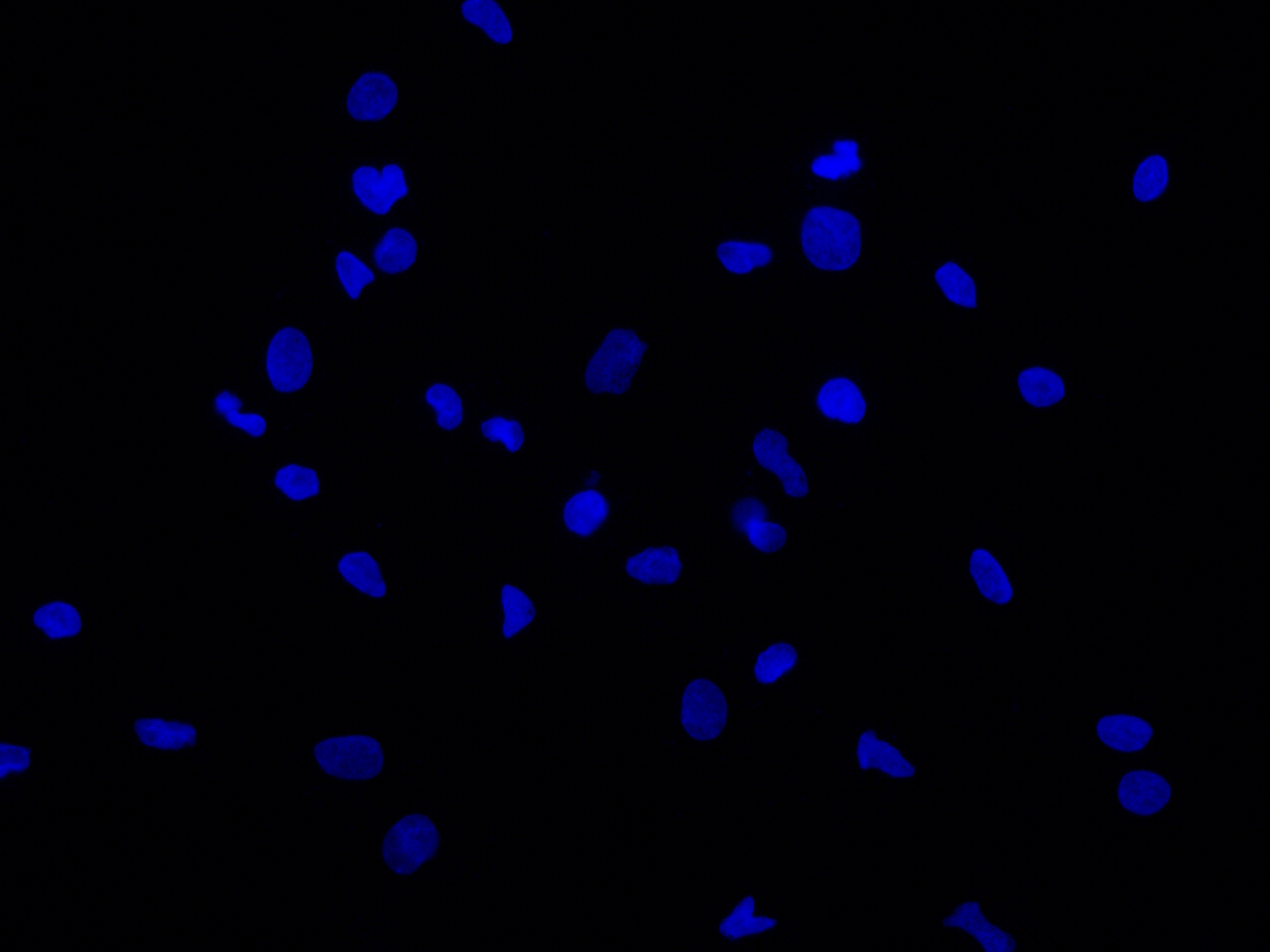

Supplement: Supplementary file 2 [file DataSheet4.ZIP › Original source data 4/FIGURE3.A TUNEL-DAPI-MERGE/Sen+Ischemia/dapi.tif]

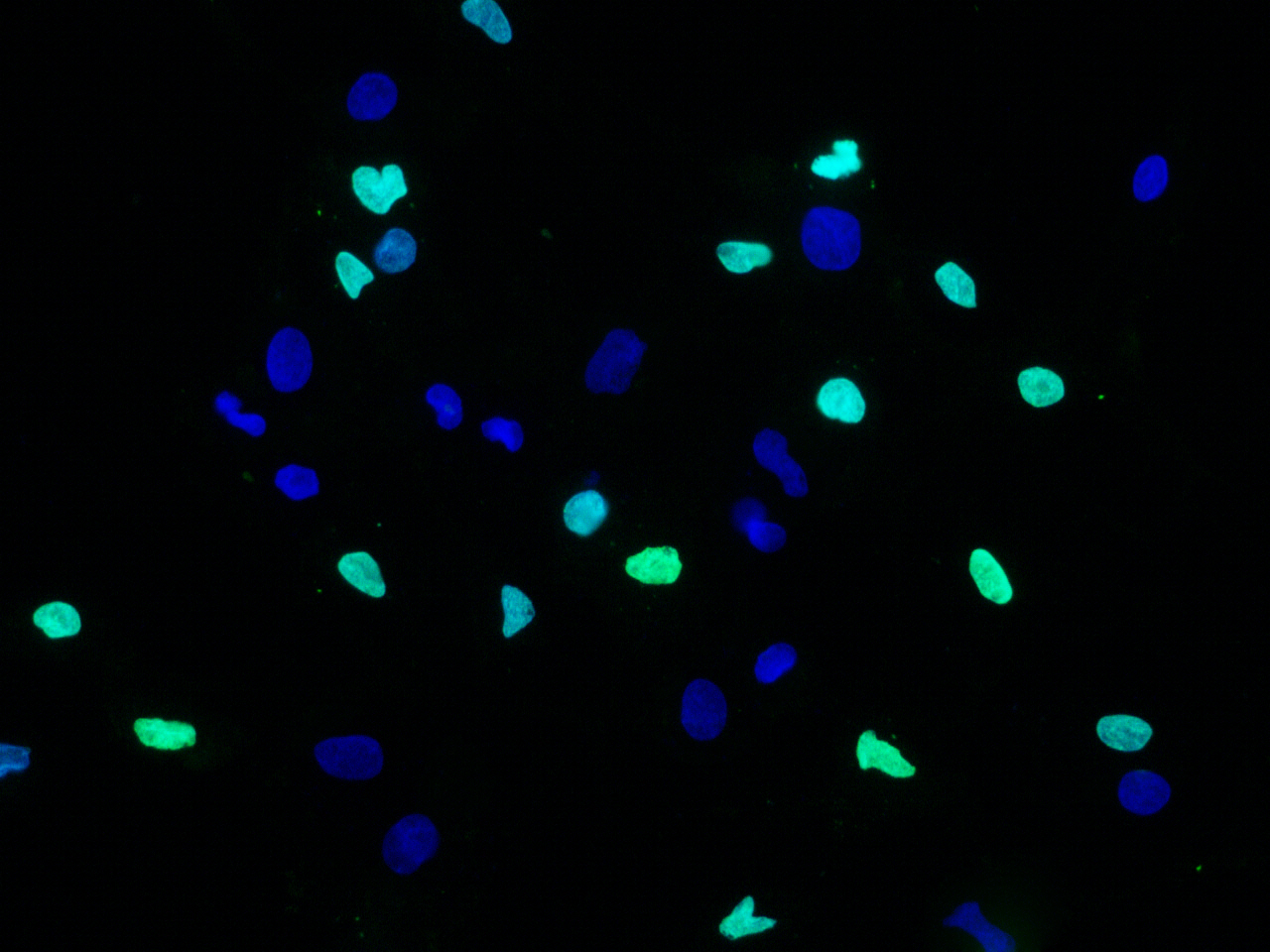

Supplement: Supplementary file 2 [file DataSheet4.ZIP › Original source data 4/FIGURE3.A TUNEL-DAPI-MERGE/Sen+Ischemia/merge.tif]

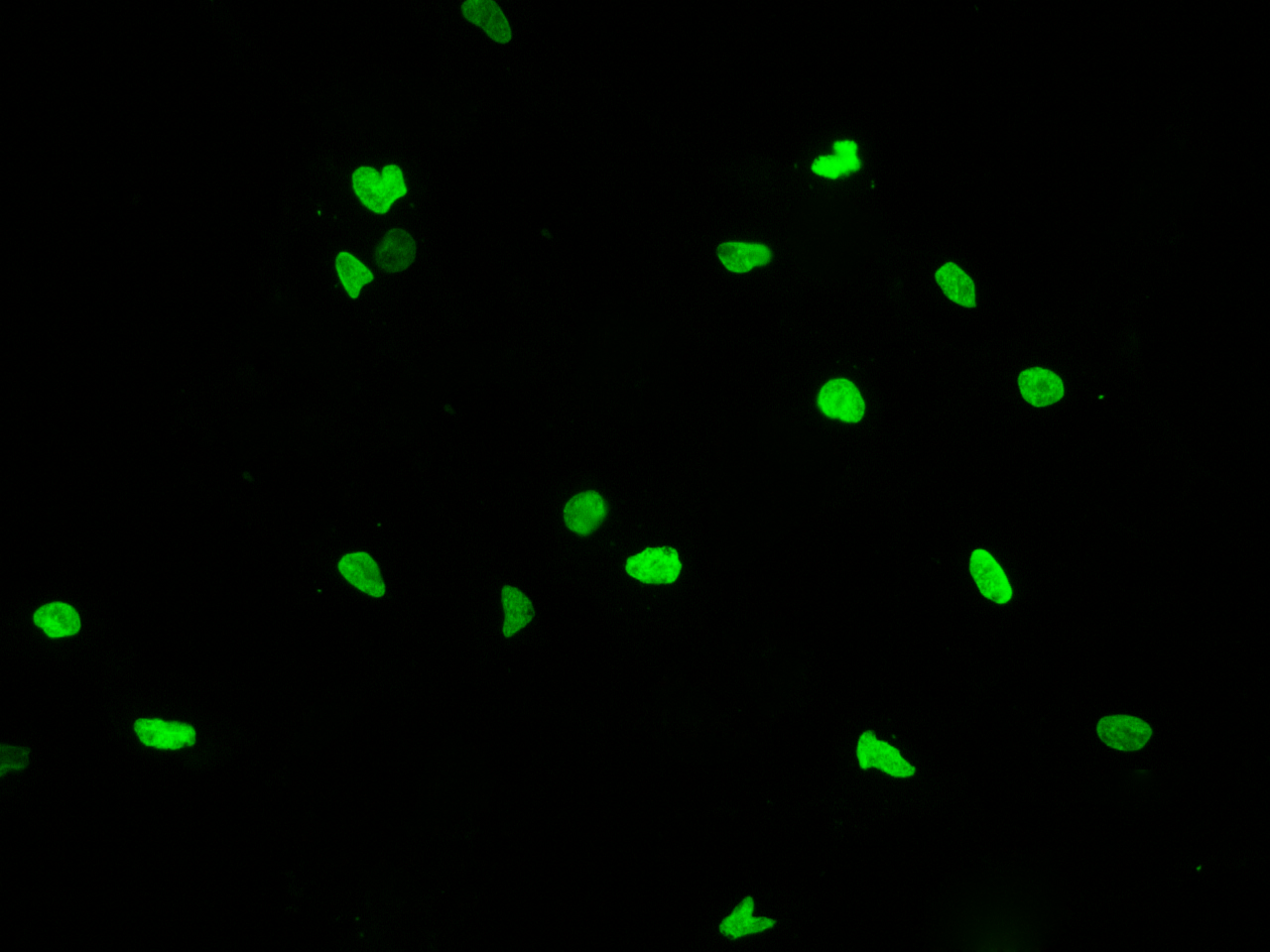

Supplement: Supplementary file 2 [file DataSheet4.ZIP › Original source data 4/FIGURE3.A TUNEL-DAPI-MERGE/Sen+Ischemia/tunel.tif]

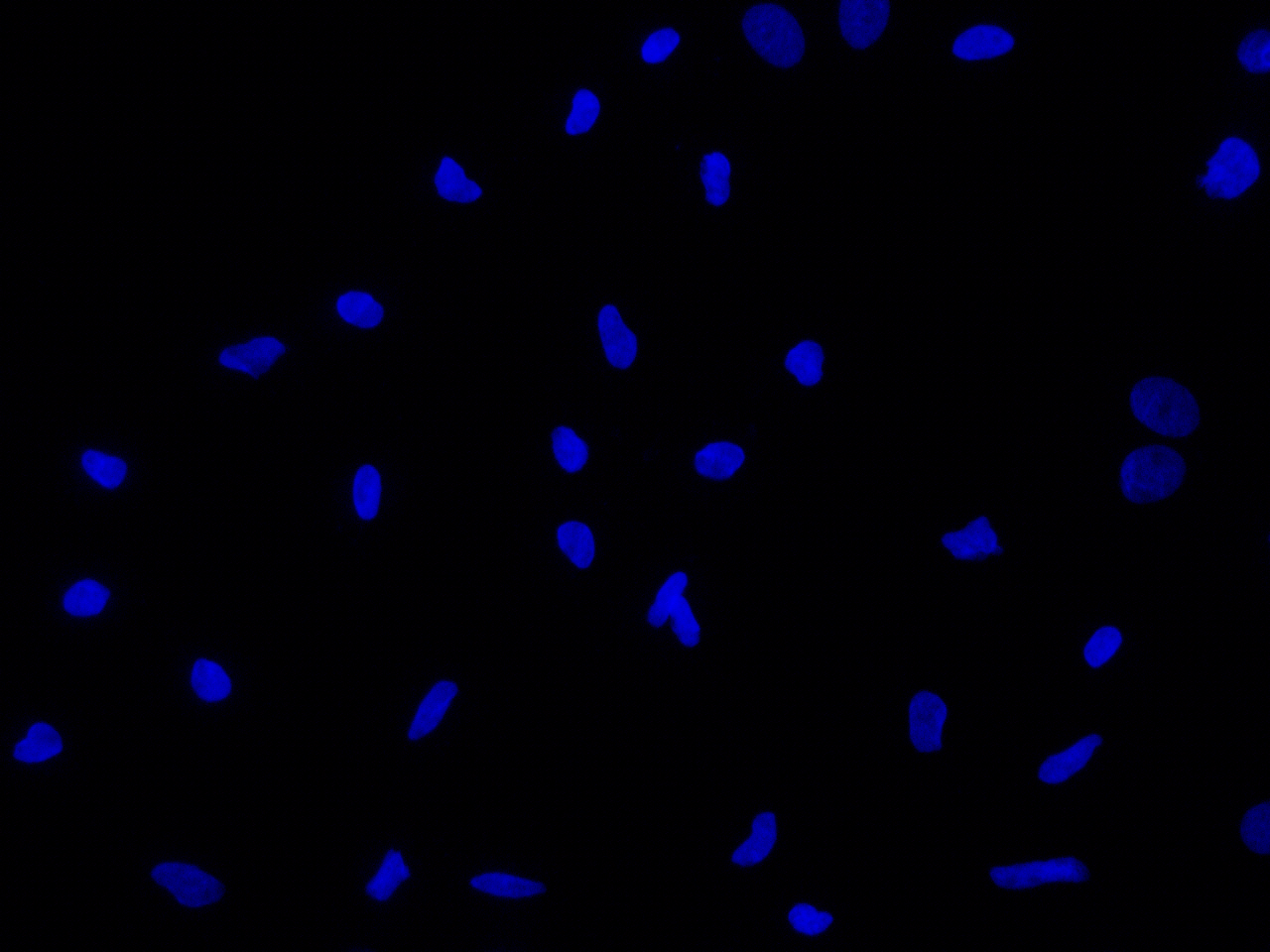

Supplement: Supplementary file 2 [file DataSheet4.ZIP › Original source data 4/FIGURE3.A TUNEL-DAPI-MERGE/Sen+Ischemia+NAC/dapi.tif]

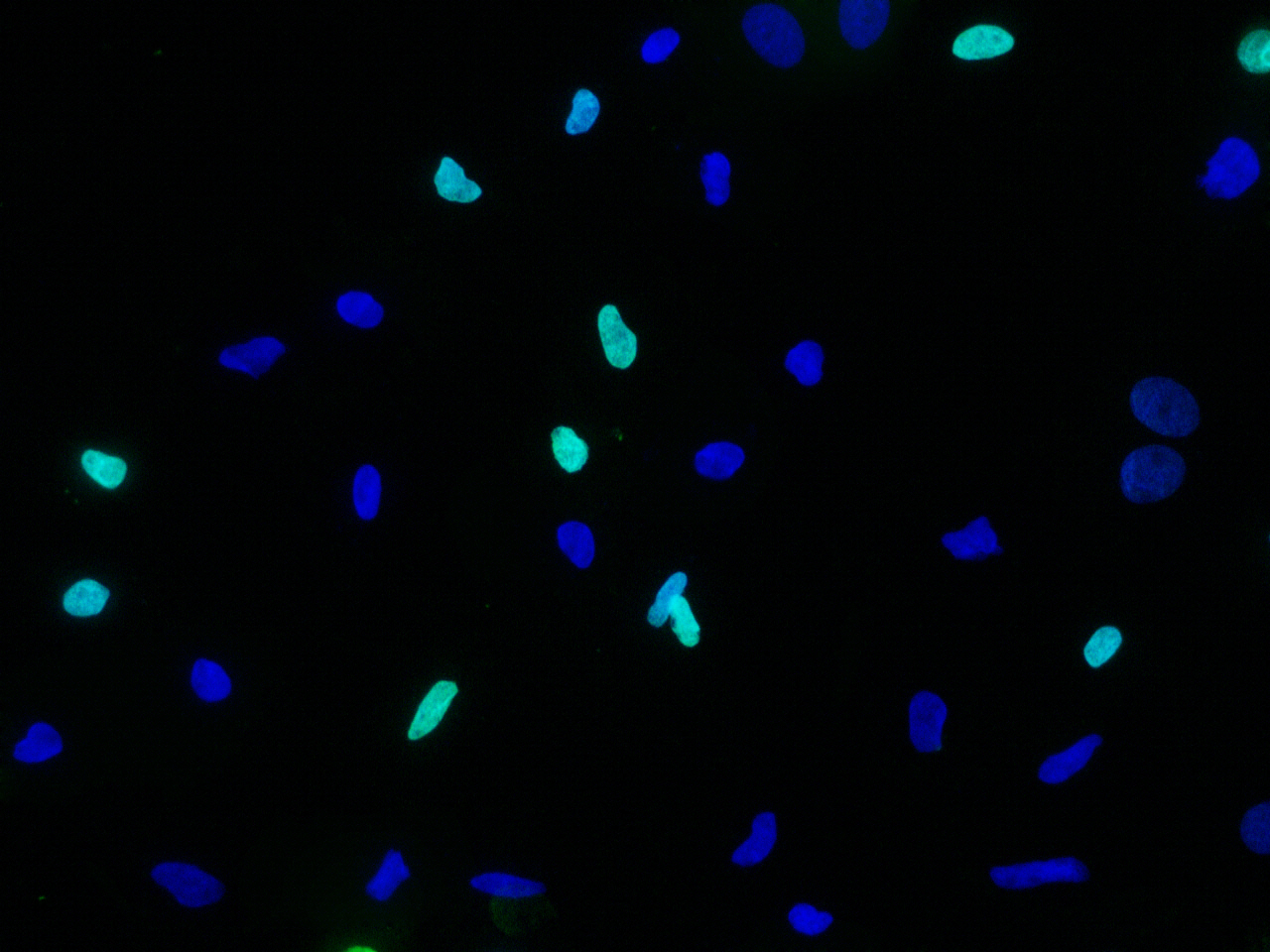

Supplement: Supplementary file 2 [file DataSheet4.ZIP › Original source data 4/FIGURE3.A TUNEL-DAPI-MERGE/Sen+Ischemia+NAC/merge.tif]

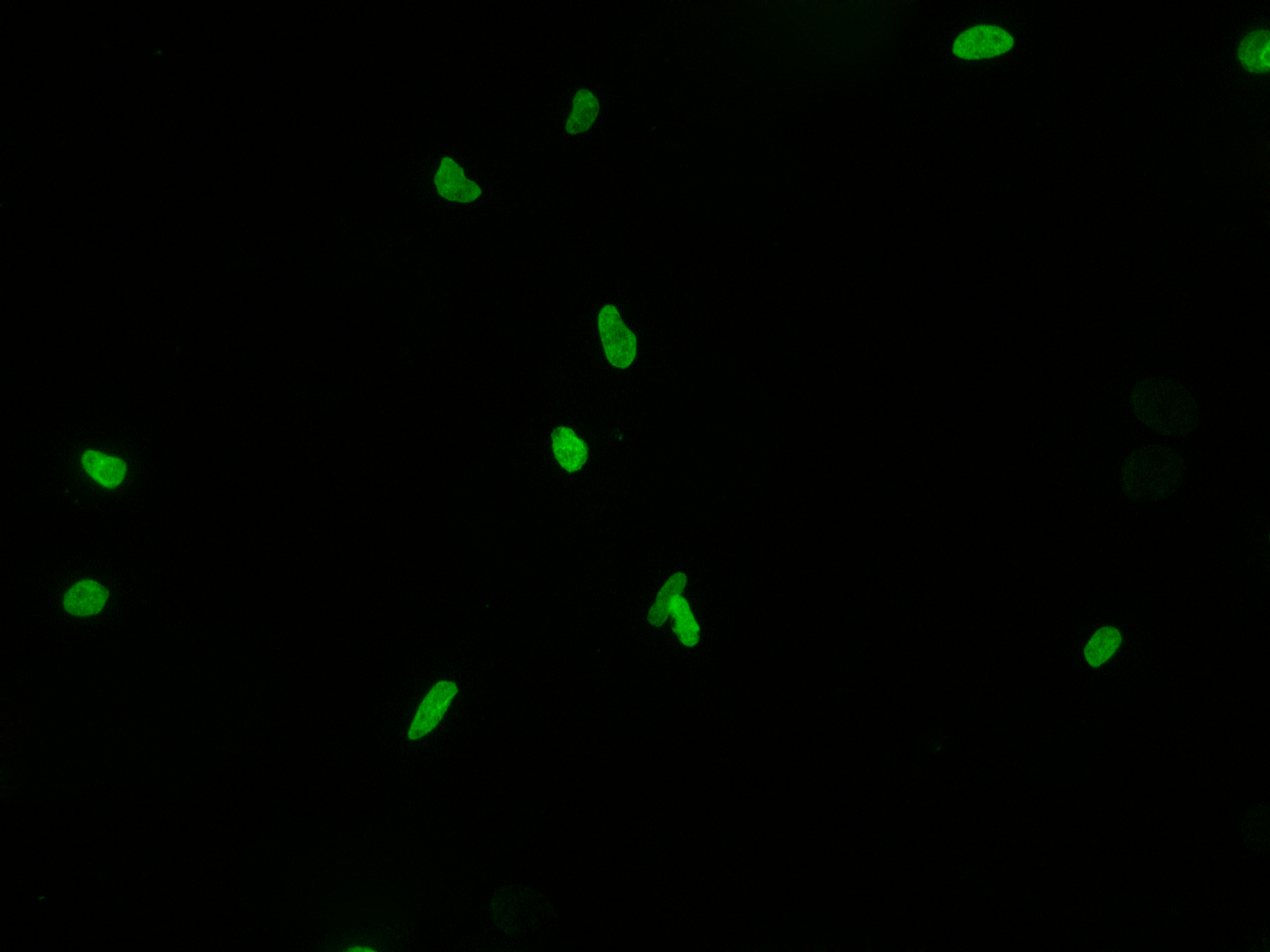

Supplement: Supplementary file 2 [file DataSheet4.ZIP › Original source data 4/FIGURE3.A TUNEL-DAPI-MERGE/Sen+Ischemia+NAC/tunel.tif]

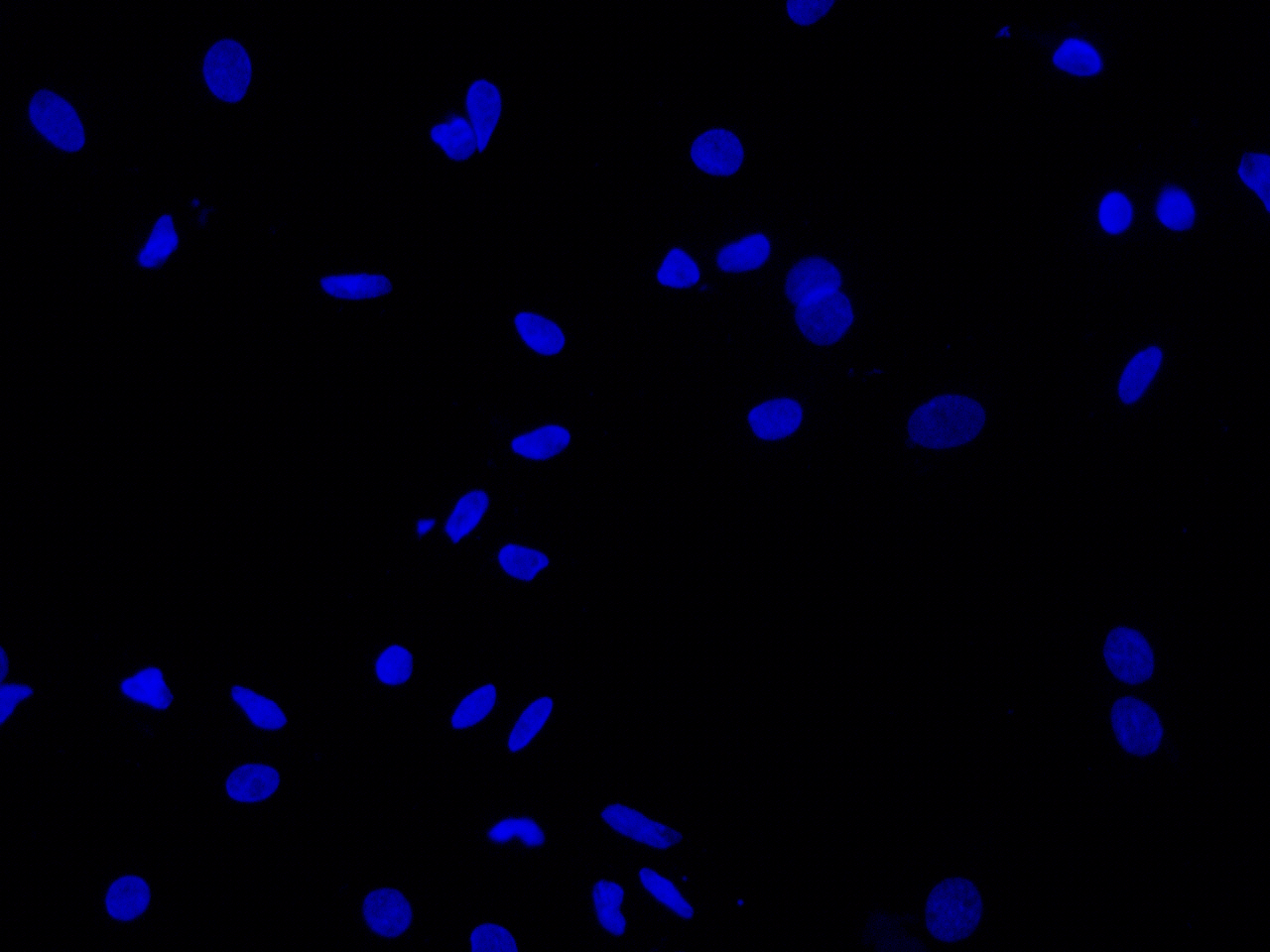

Supplement: Supplementary file 2 [file DataSheet4.ZIP › Original source data 4/FIGURE3.A TUNEL-DAPI-MERGE/Sen+Ischemia+SiCys/dapi.tif]

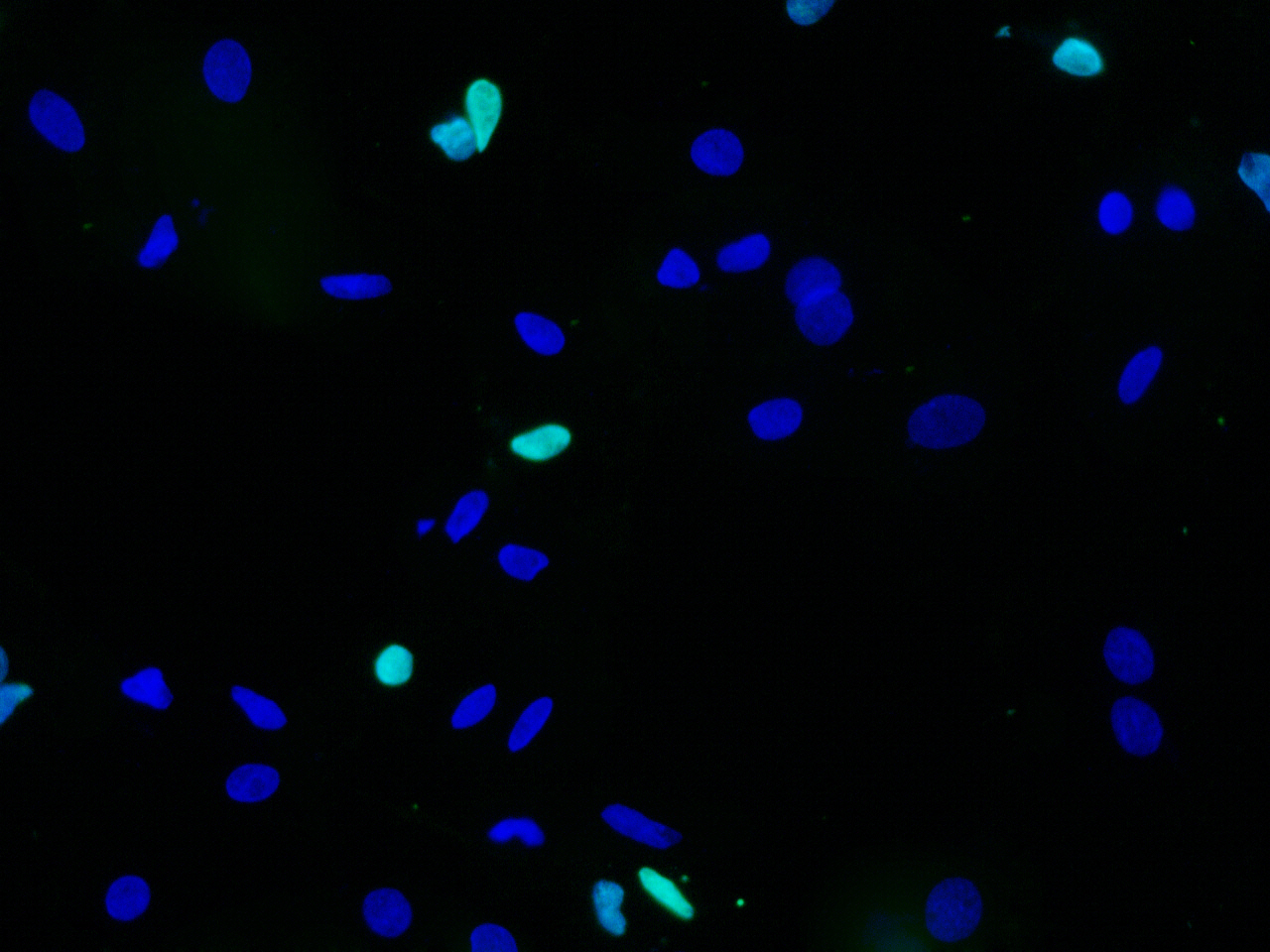

Supplement: Supplementary file 2 [file DataSheet4.ZIP › Original source data 4/FIGURE3.A TUNEL-DAPI-MERGE/Sen+Ischemia+SiCys/merge.tif]

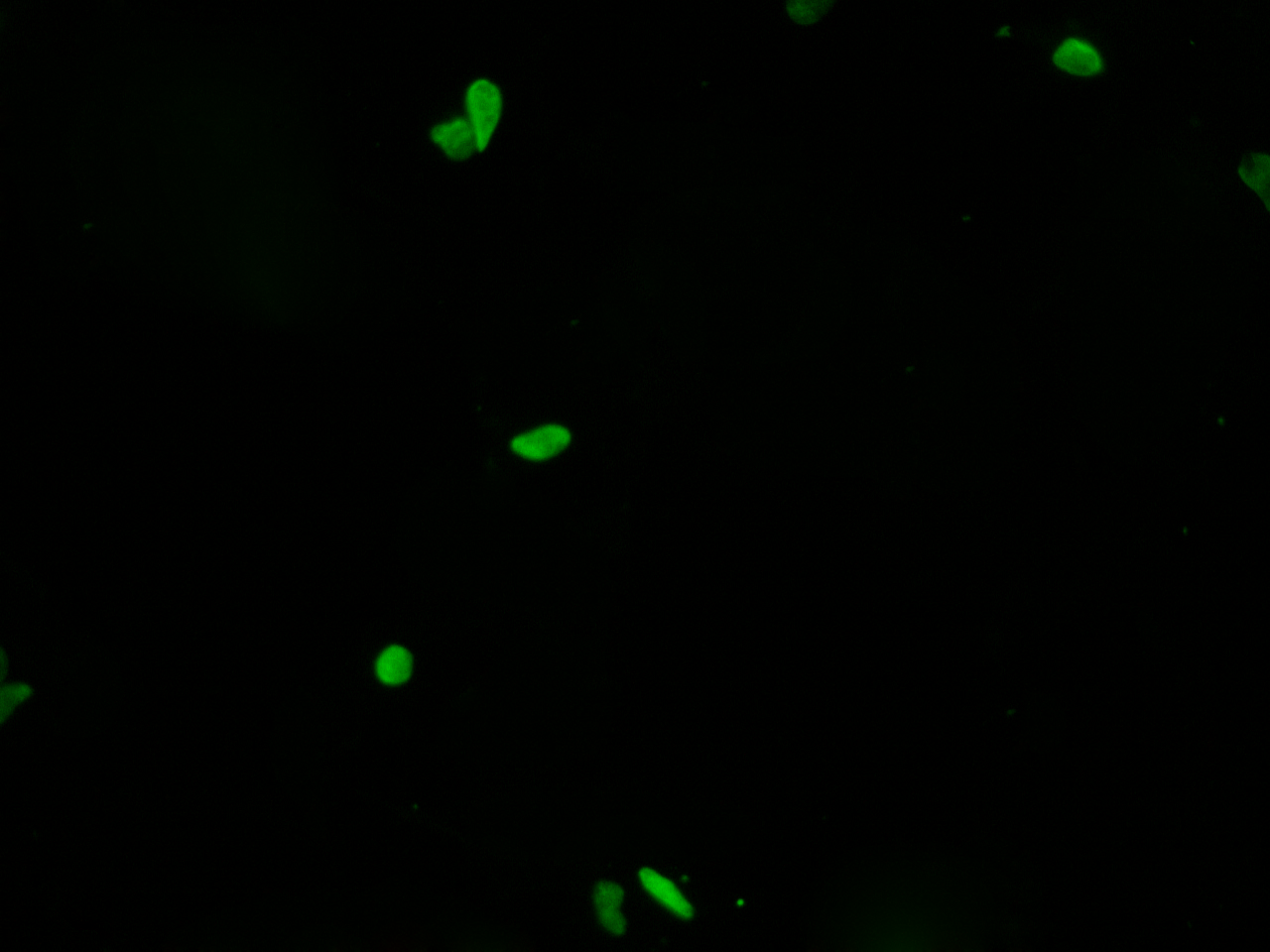

Supplement: Supplementary file 2 [file DataSheet4.ZIP › Original source data 4/FIGURE3.A TUNEL-DAPI-MERGE/Sen+Ischemia+SiCys/tunel.tif]

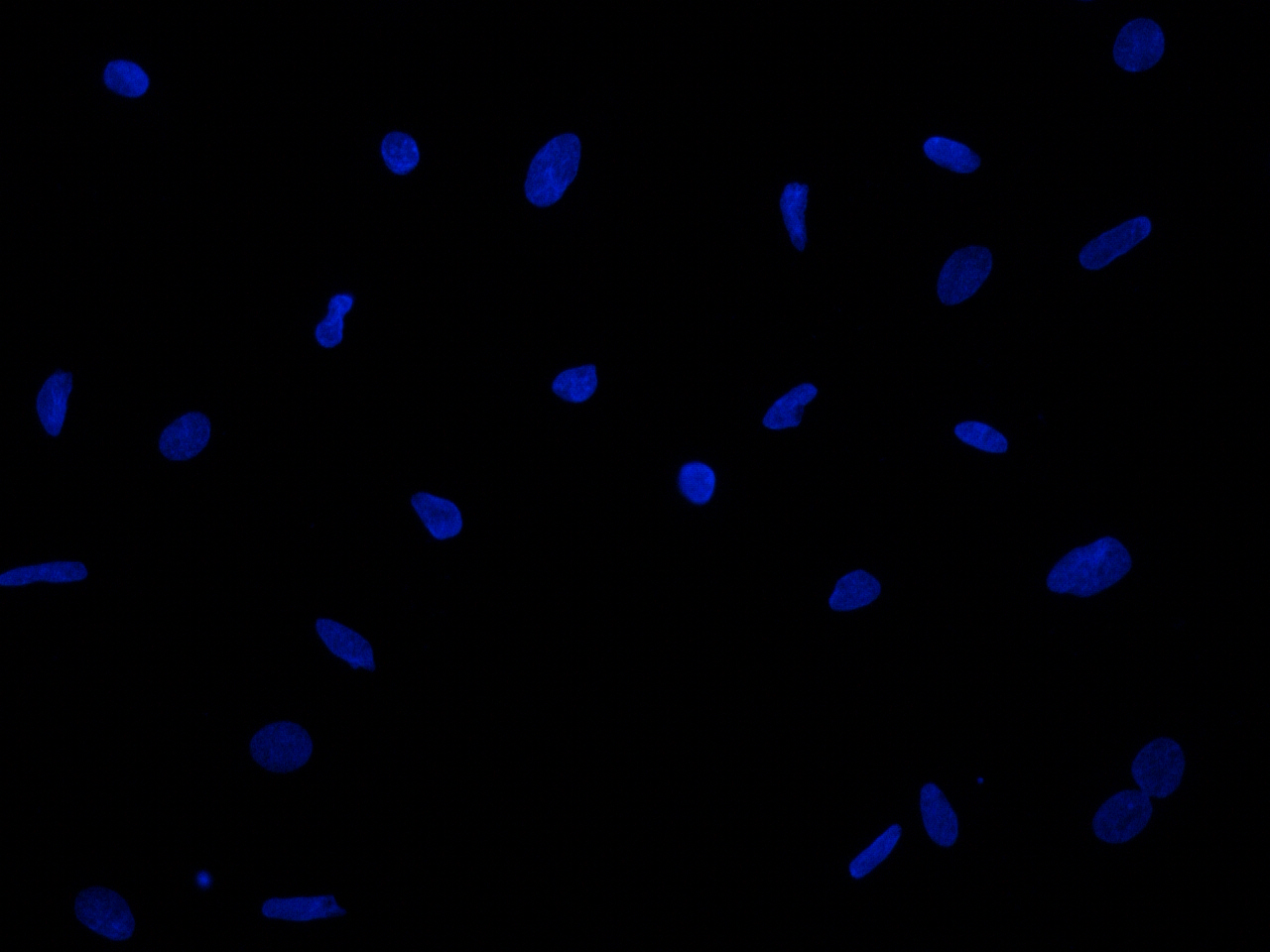

Supplement: Supplementary file 2 [file DataSheet4.ZIP › Original source data 4/FIGURE3.A TUNEL-DAPI-MERGE/Sen+Ischemia+Wnt3a/dapi.tif]

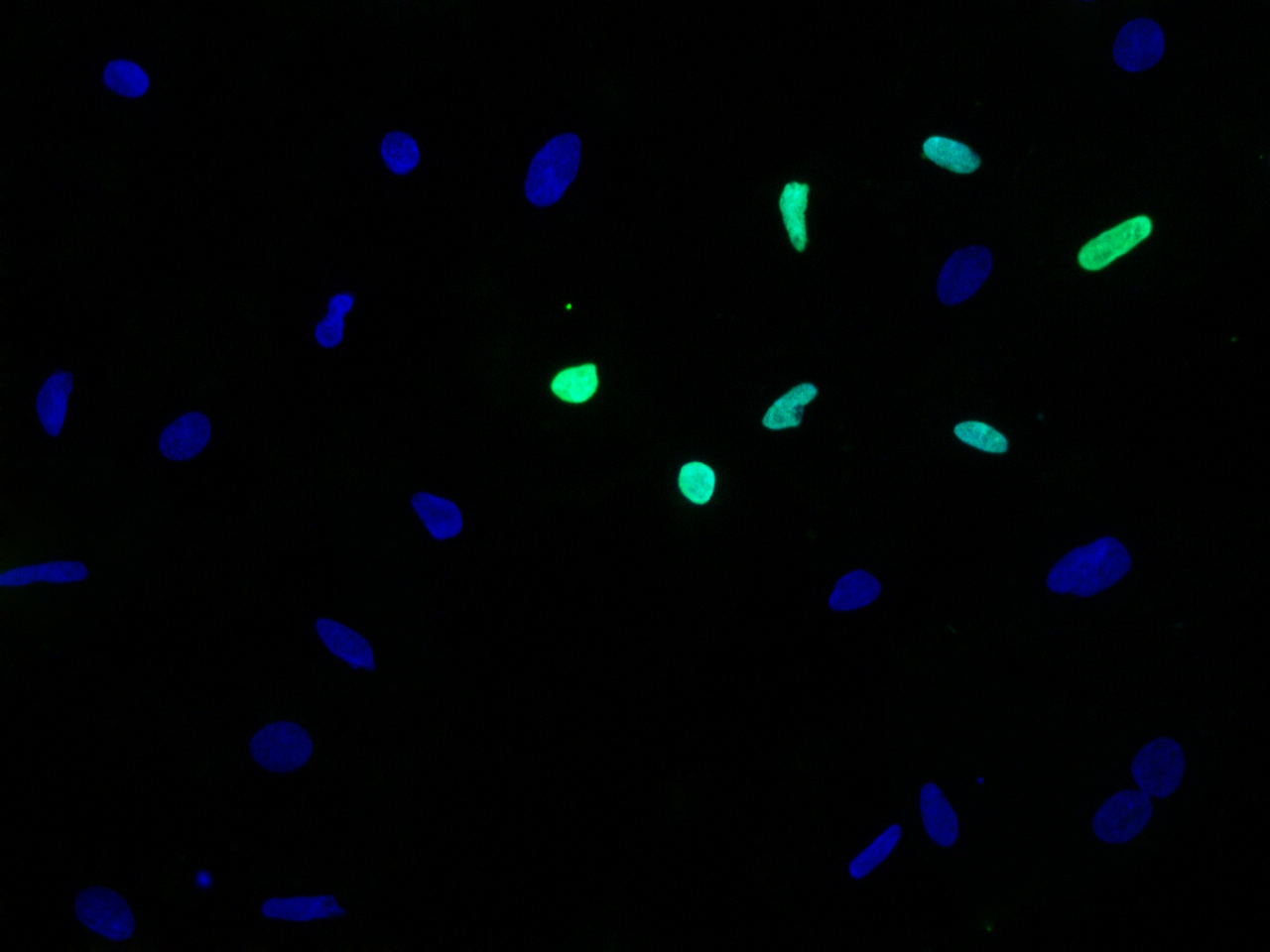

Supplement: Supplementary file 2 [file DataSheet4.ZIP › Original source data 4/FIGURE3.A TUNEL-DAPI-MERGE/Sen+Ischemia+Wnt3a/merge.tif]

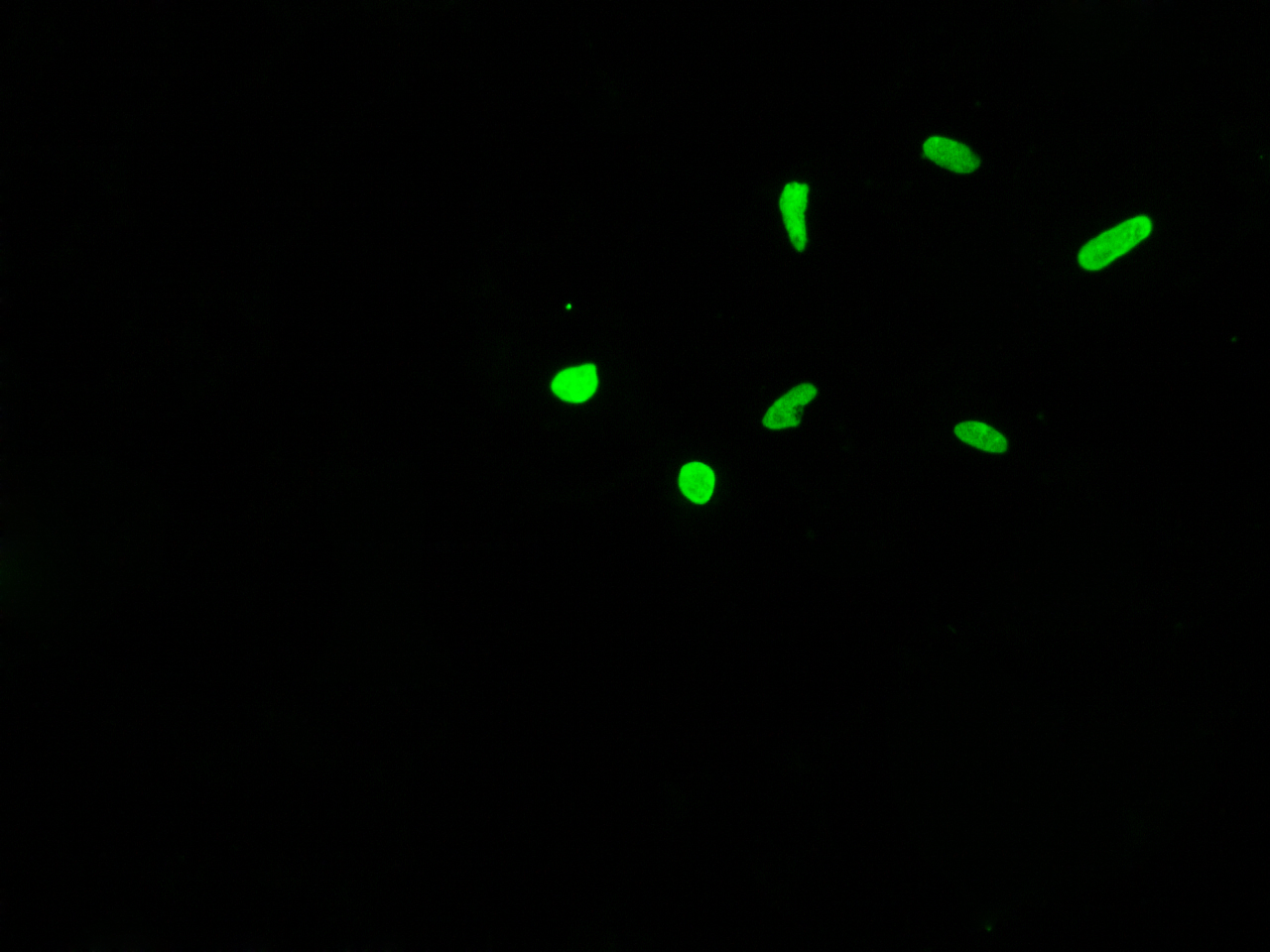

Supplement: Supplementary file 2 [file DataSheet4.ZIP › Original source data 4/FIGURE3.A TUNEL-DAPI-MERGE/Sen+Ischemia+Wnt3a/tunel.tif]

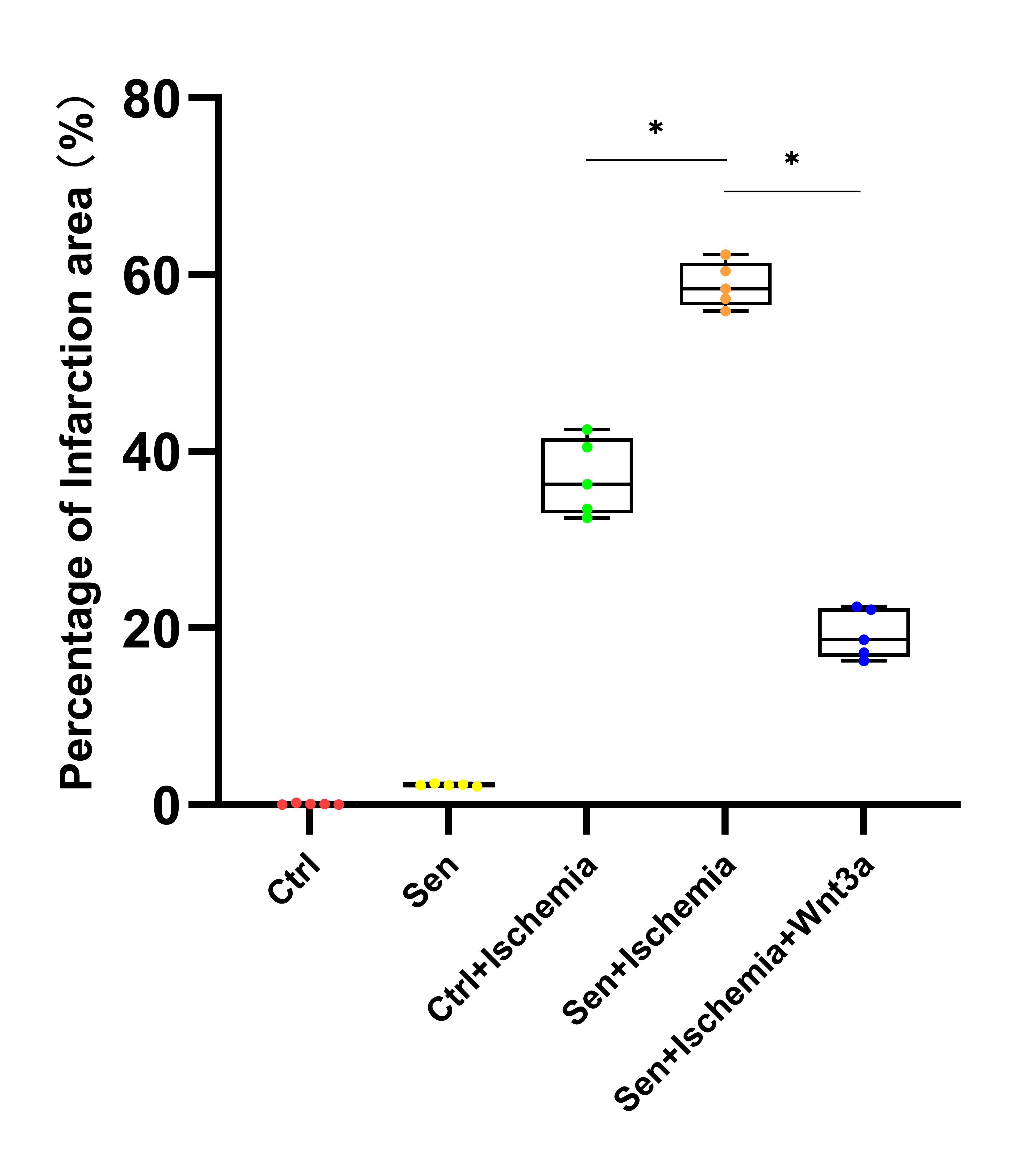

Supplement: Supplementary file 3 [file DataSheet1.ZIP › Original source data 1/Prism data and figures/FIGURE1.C.jpg]

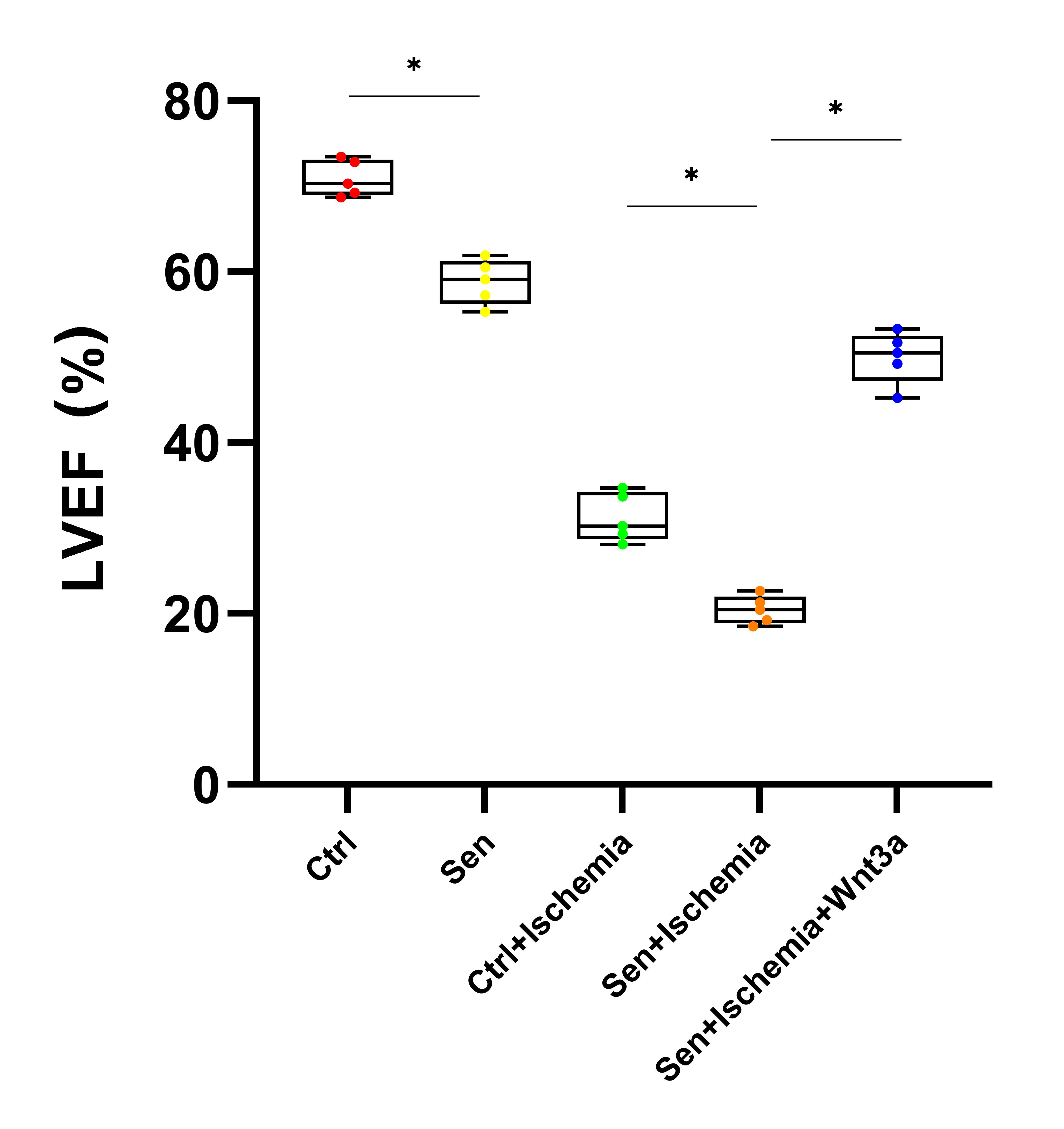

Supplement: Supplementary file 3 [file DataSheet1.ZIP › Original source data 1/Prism data and figures/FIGURE1.D.jpg]

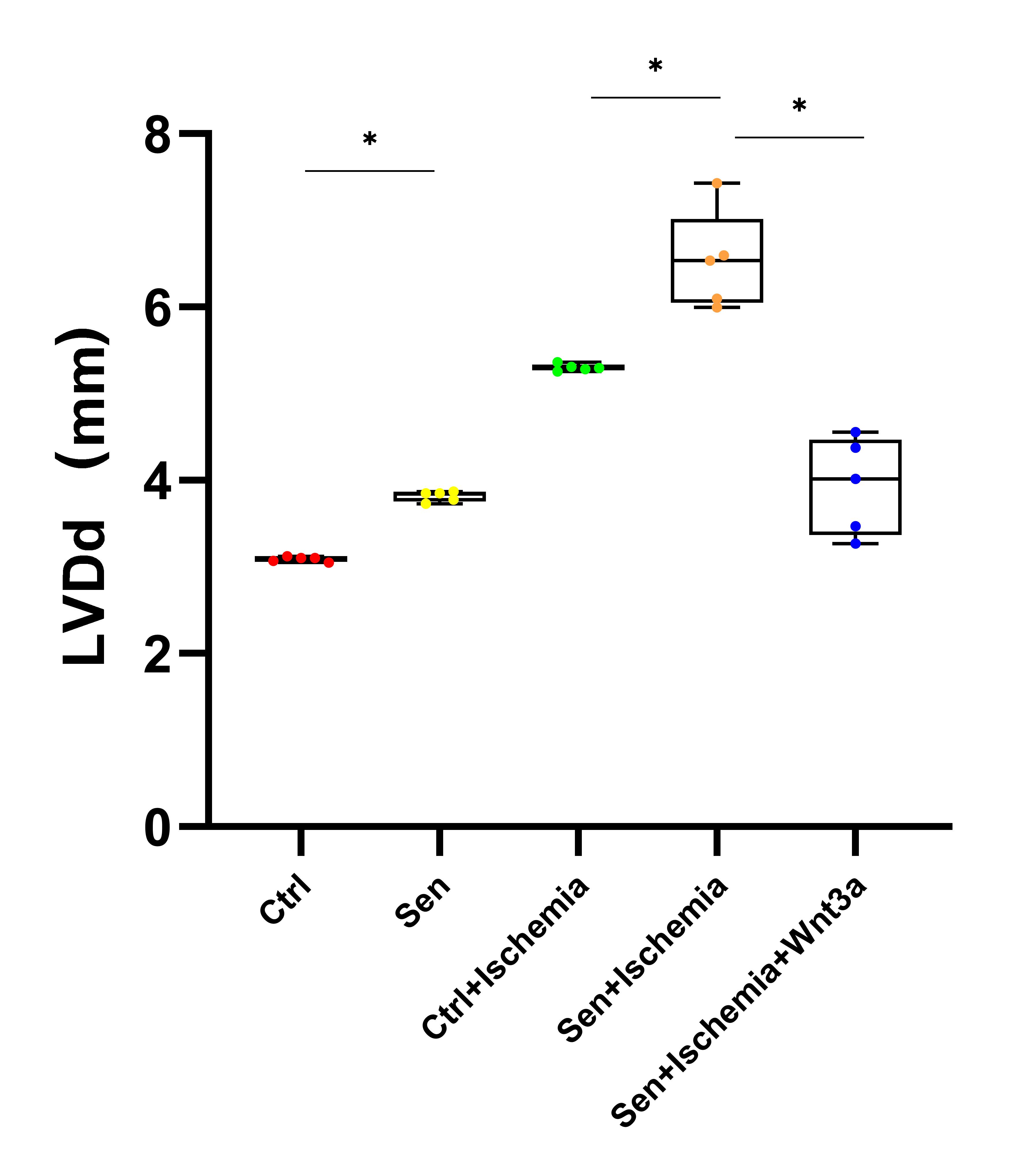

Supplement: Supplementary file 3 [file DataSheet1.ZIP › Original source data 1/Prism data and figures/FIGURE1.E.jpg]

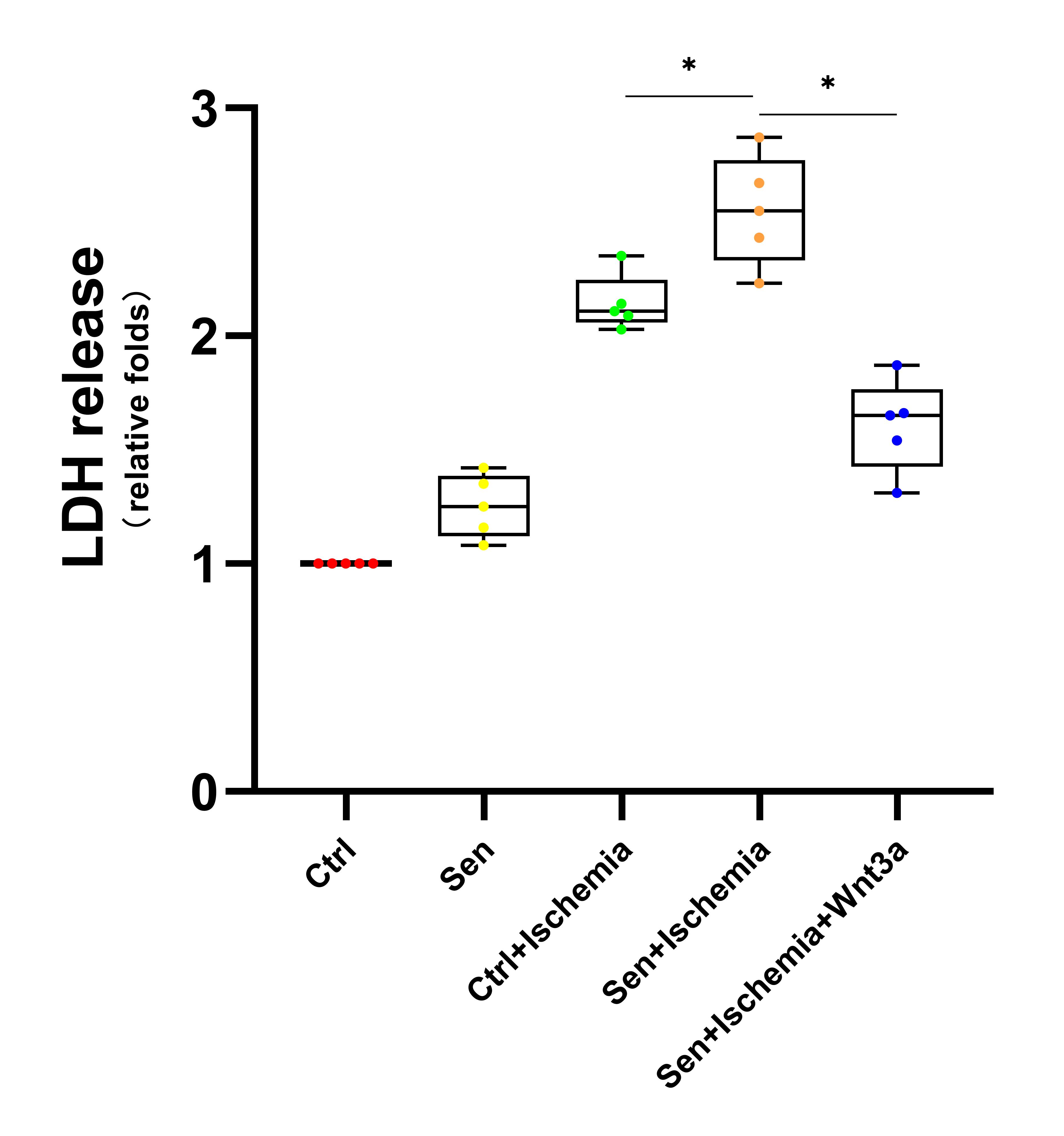

Supplement: Supplementary file 3 [file DataSheet1.ZIP › Original source data 1/Prism data and figures/FIGURE1.F.jpg]

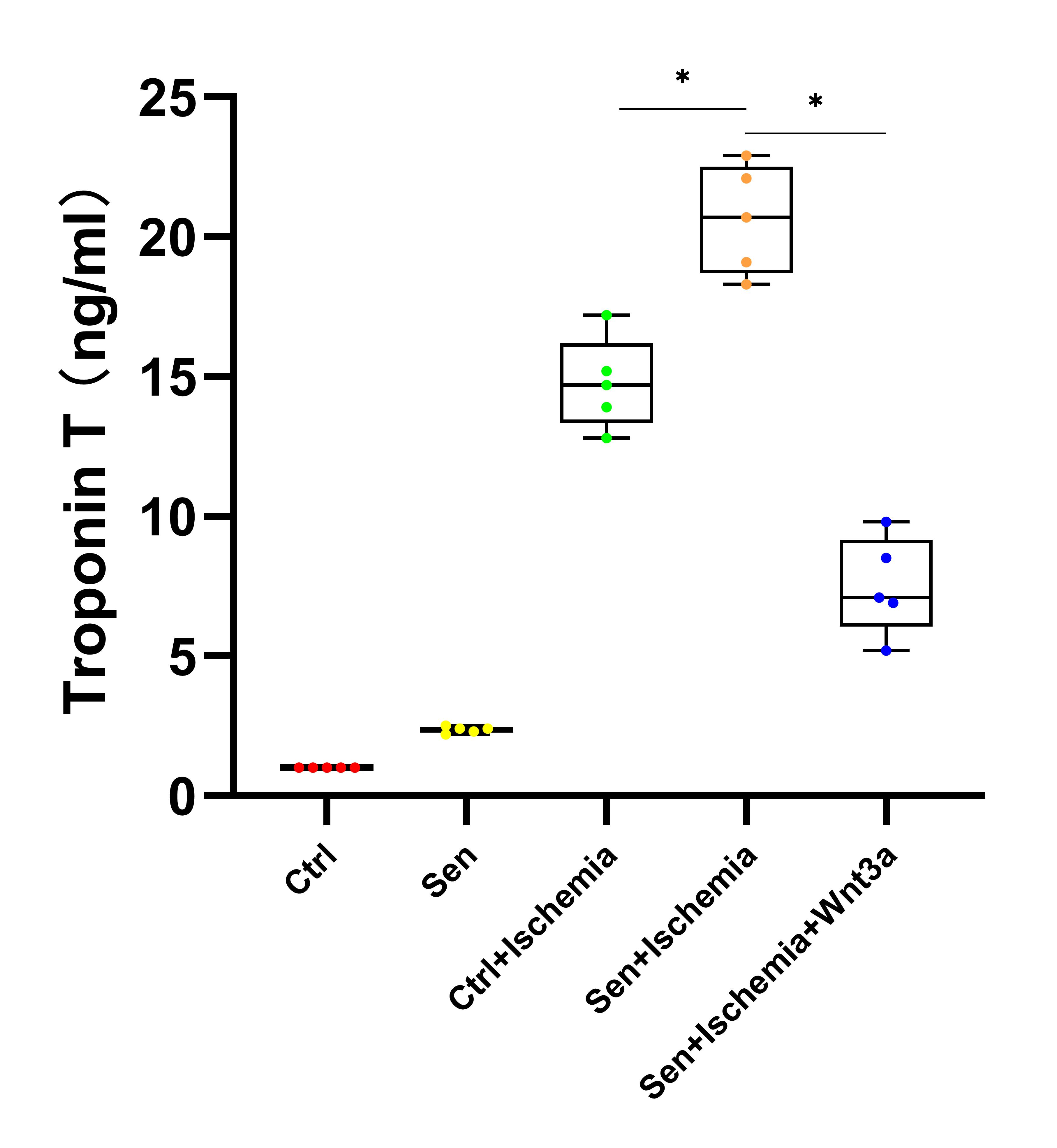

Supplement: Supplementary file 3 [file DataSheet1.ZIP › Original source data 1/Prism data and figures/FIGURE1.G.jpg]

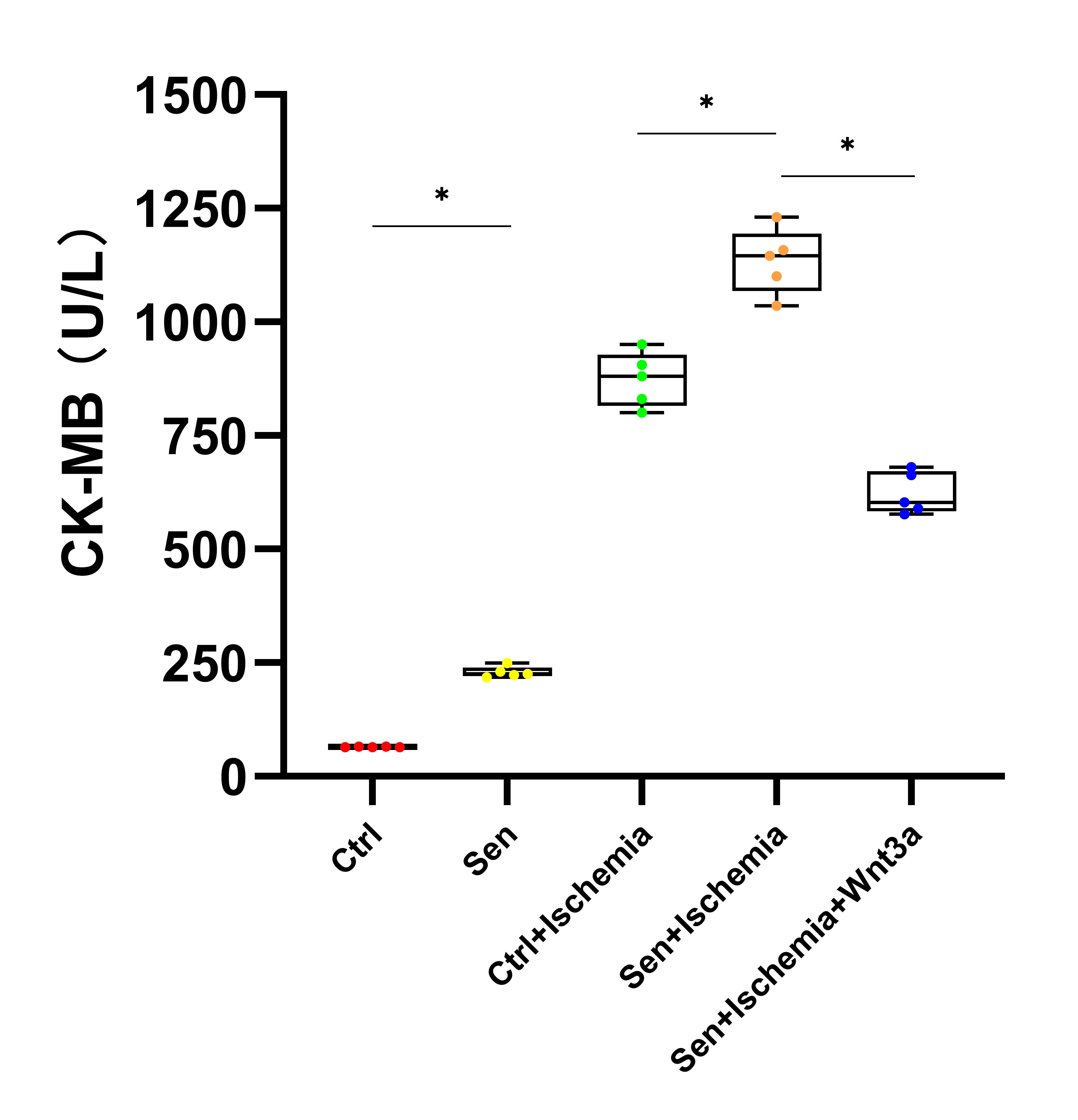

Supplement: Supplementary file 3 [file DataSheet1.ZIP › Original source data 1/Prism data and figures/FIGURE1.H.jpg]

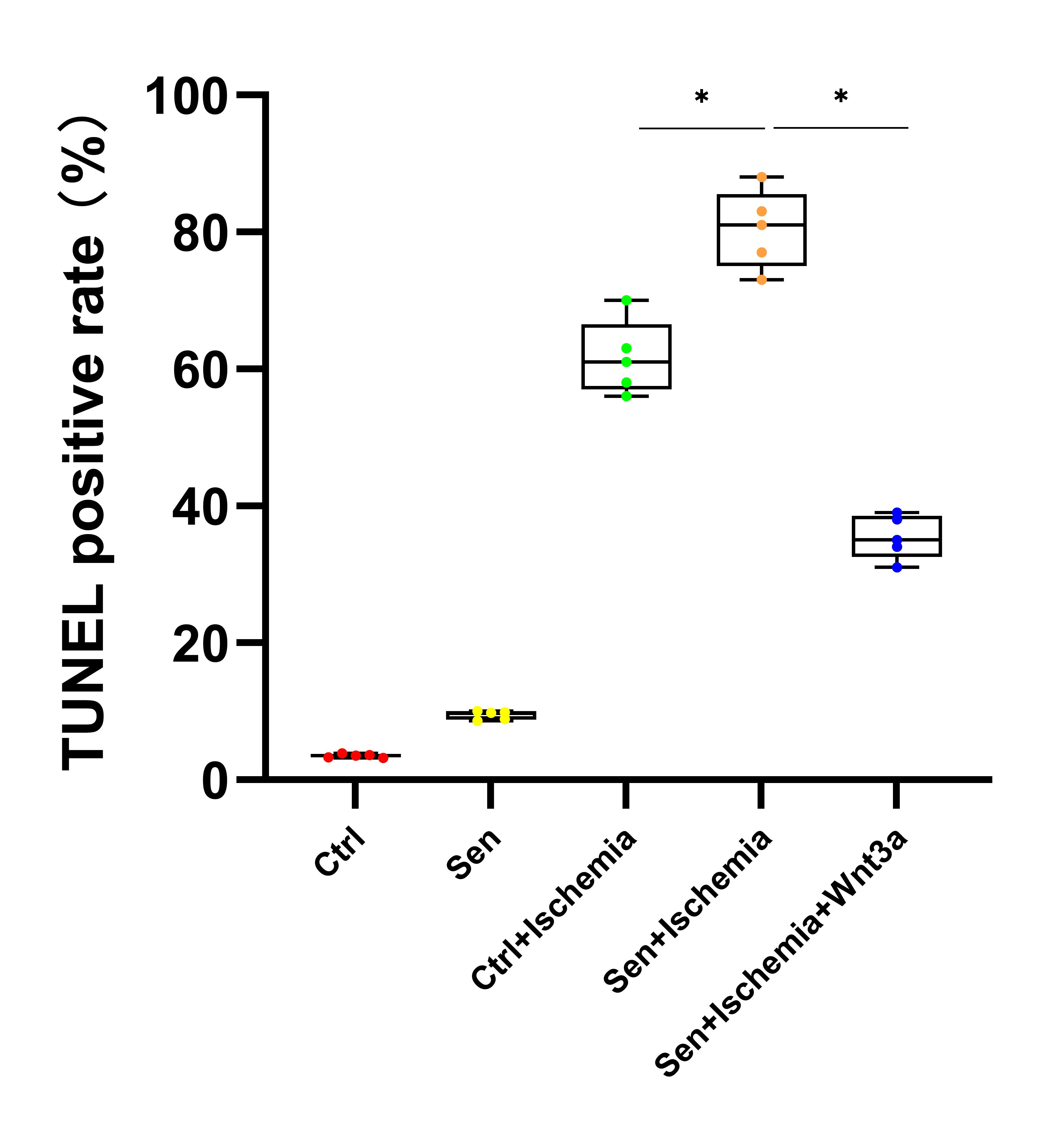

Supplement: Supplementary file 3 [file DataSheet1.ZIP › Original source data 1/Prism data and figures/FIGURE2.E.jpg]

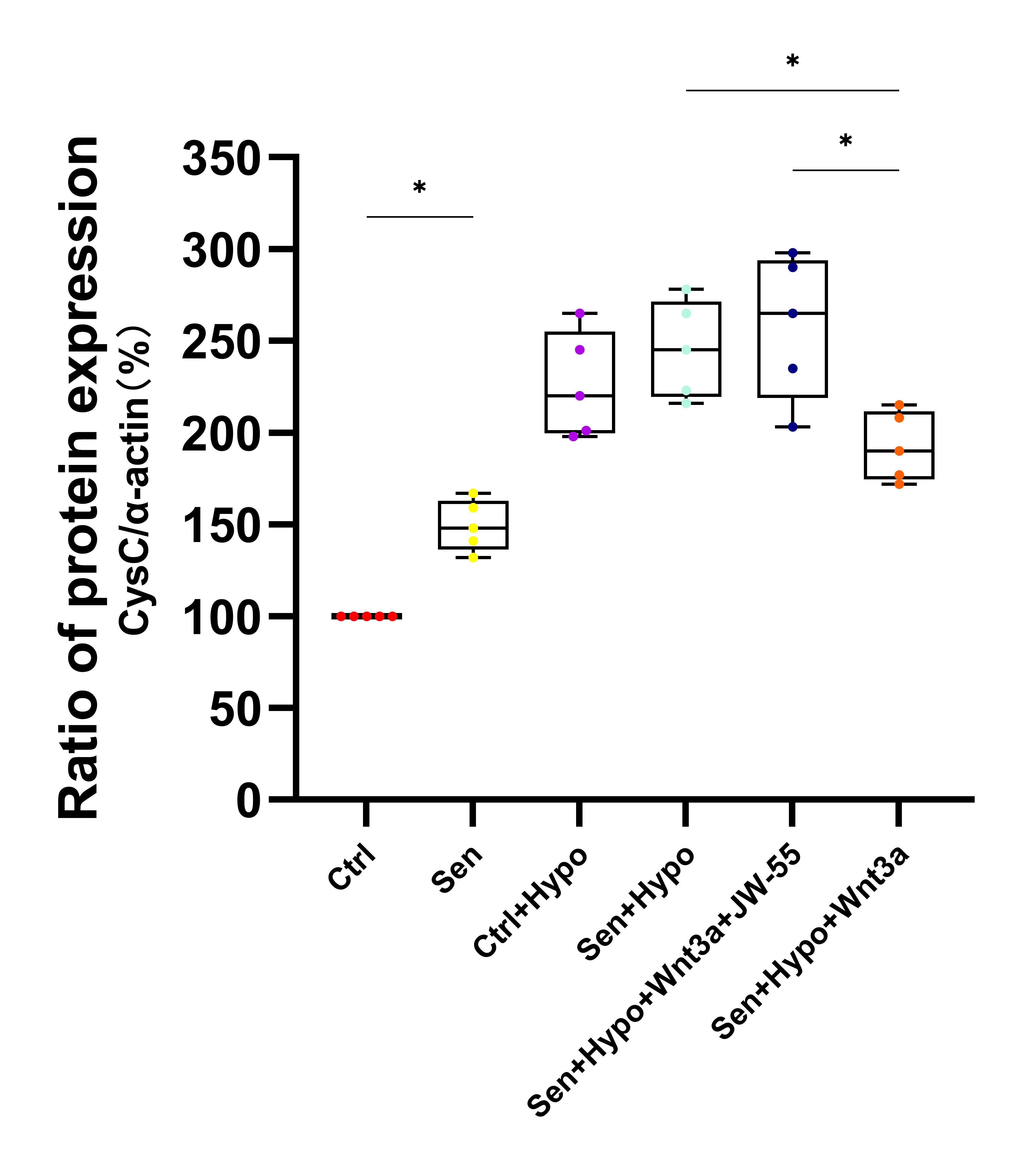

Supplement: Supplementary file 3 [file DataSheet1.ZIP › Original source data 1/Prism data and figures/FIGURE2.F.jpg]

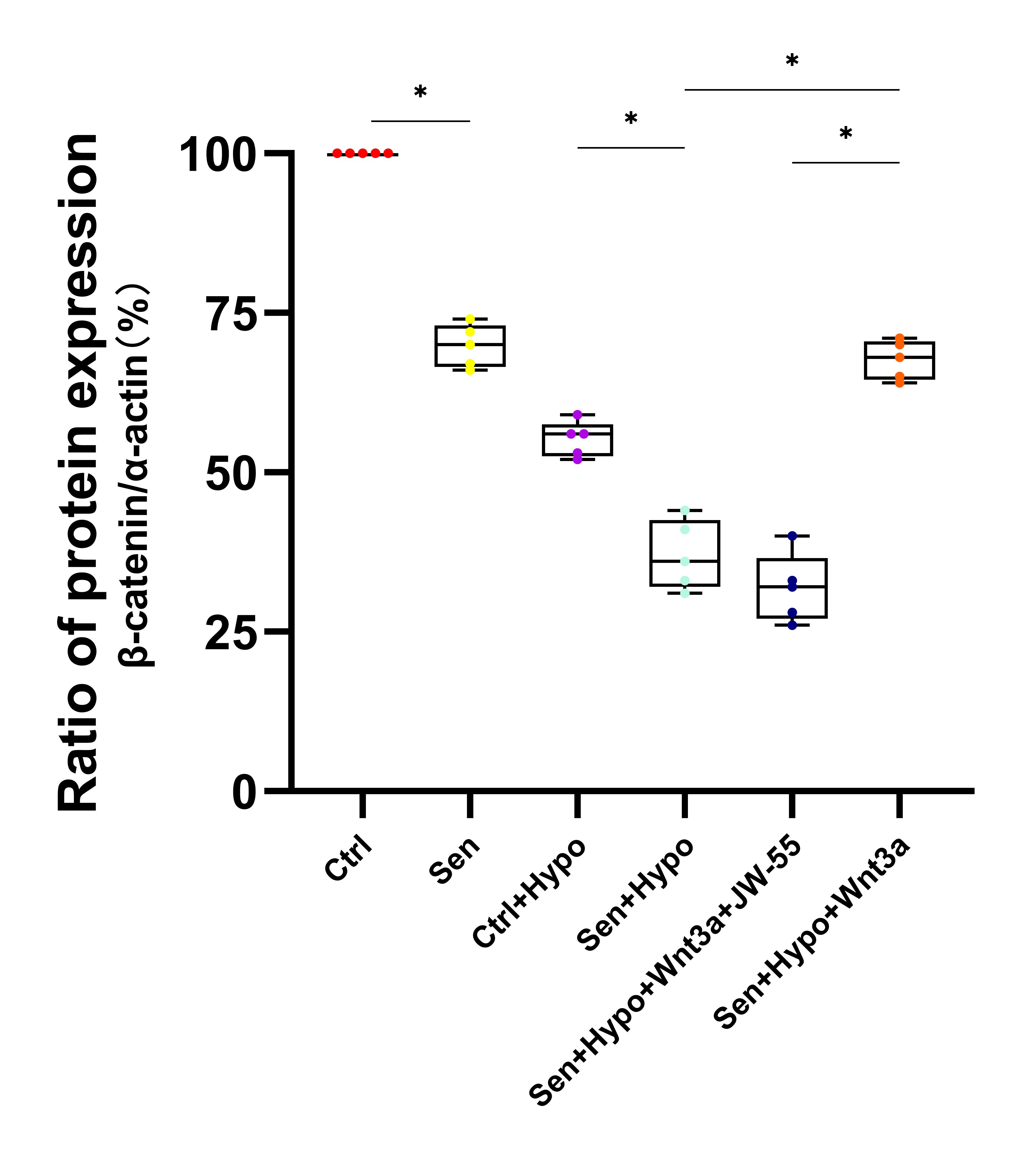

Supplement: Supplementary file 3 [file DataSheet1.ZIP › Original source data 1/Prism data and figures/FIGURE2.G.jpg]

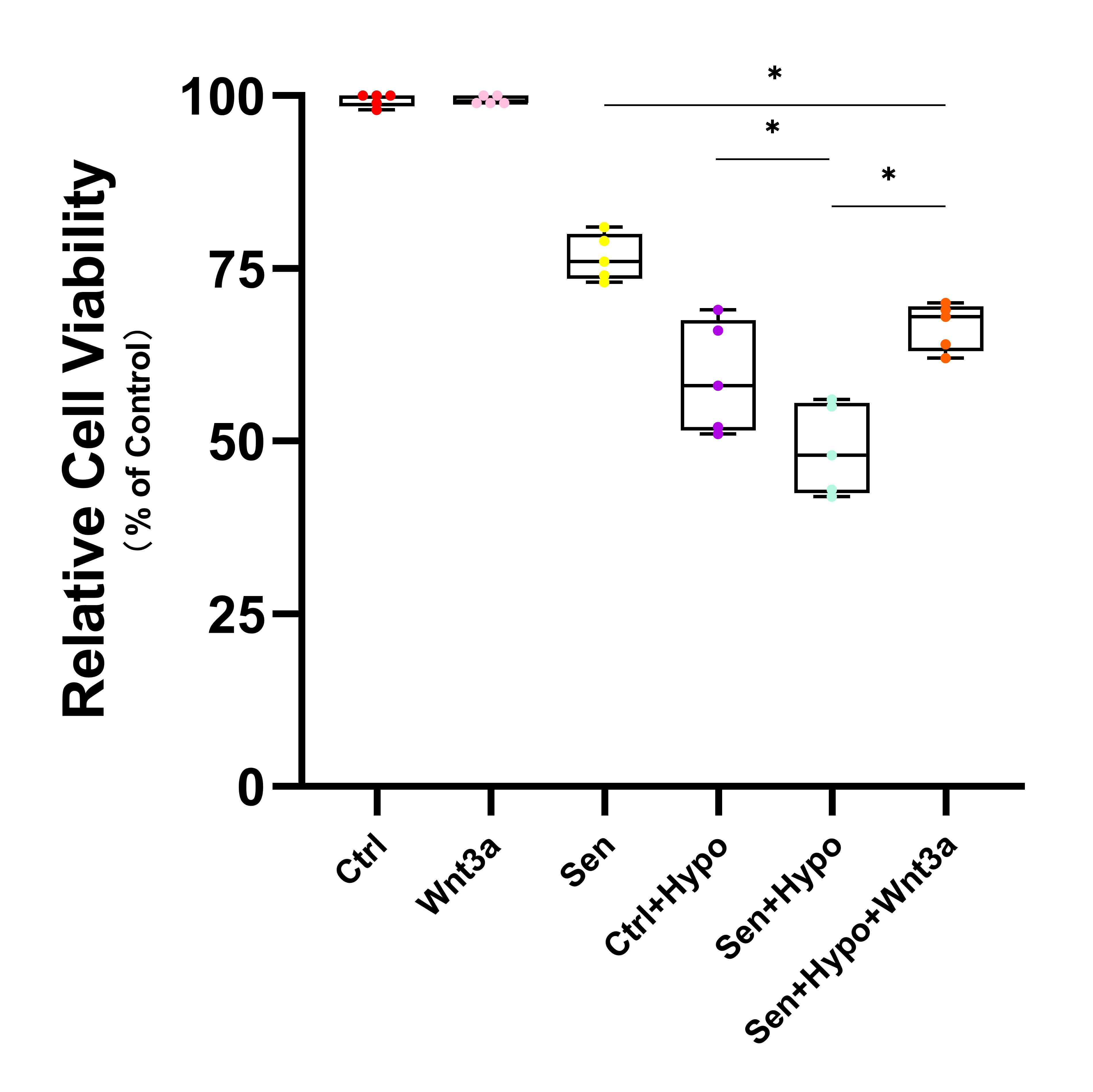

Supplement: Supplementary file 3 [file DataSheet1.ZIP › Original source data 1/Prism data and figures/FIGURE2.H.jpg]

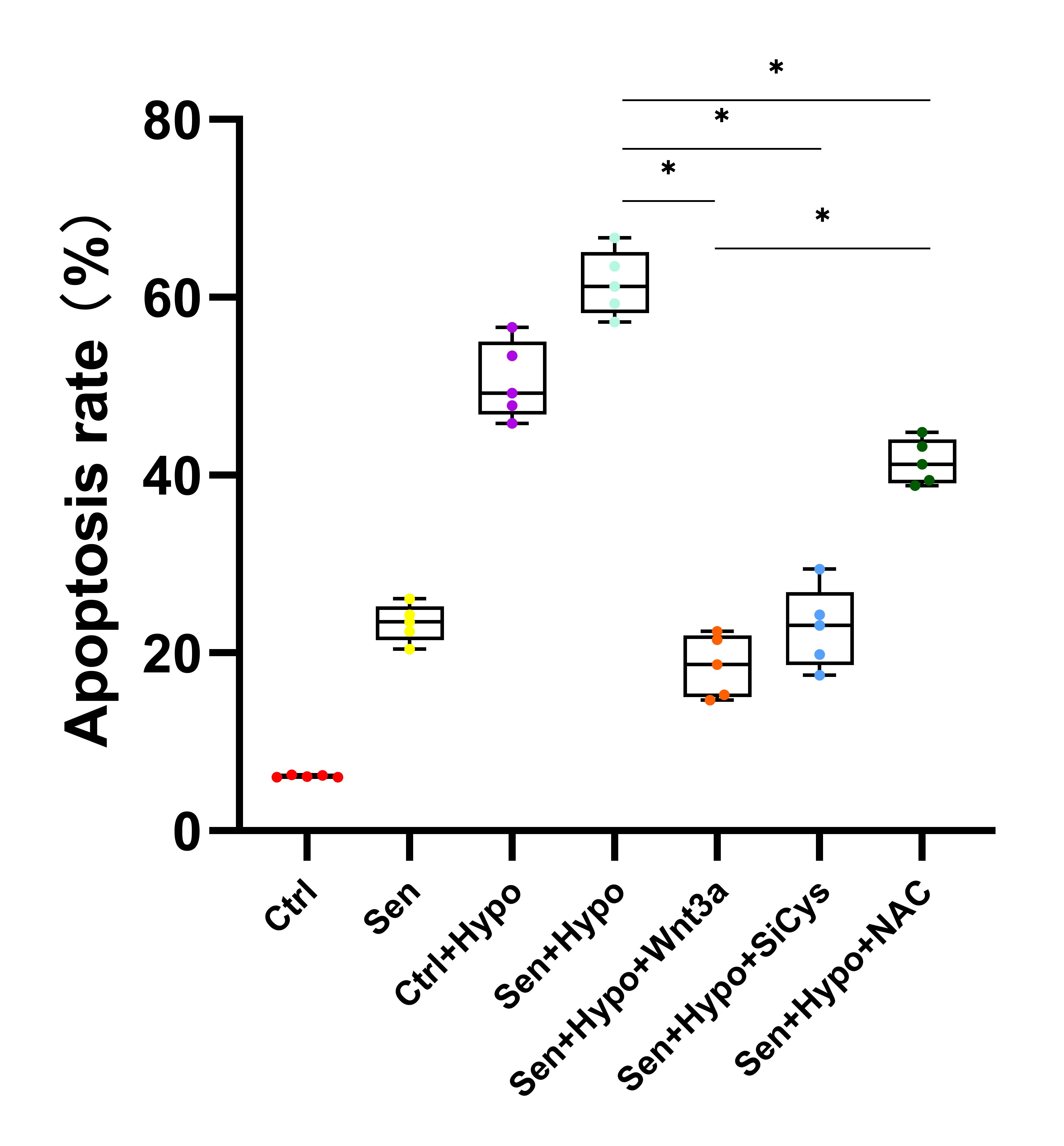

Supplement: Supplementary file 3 [file DataSheet1.ZIP › Original source data 1/Prism data and figures/FIGURE3.D.jpg]

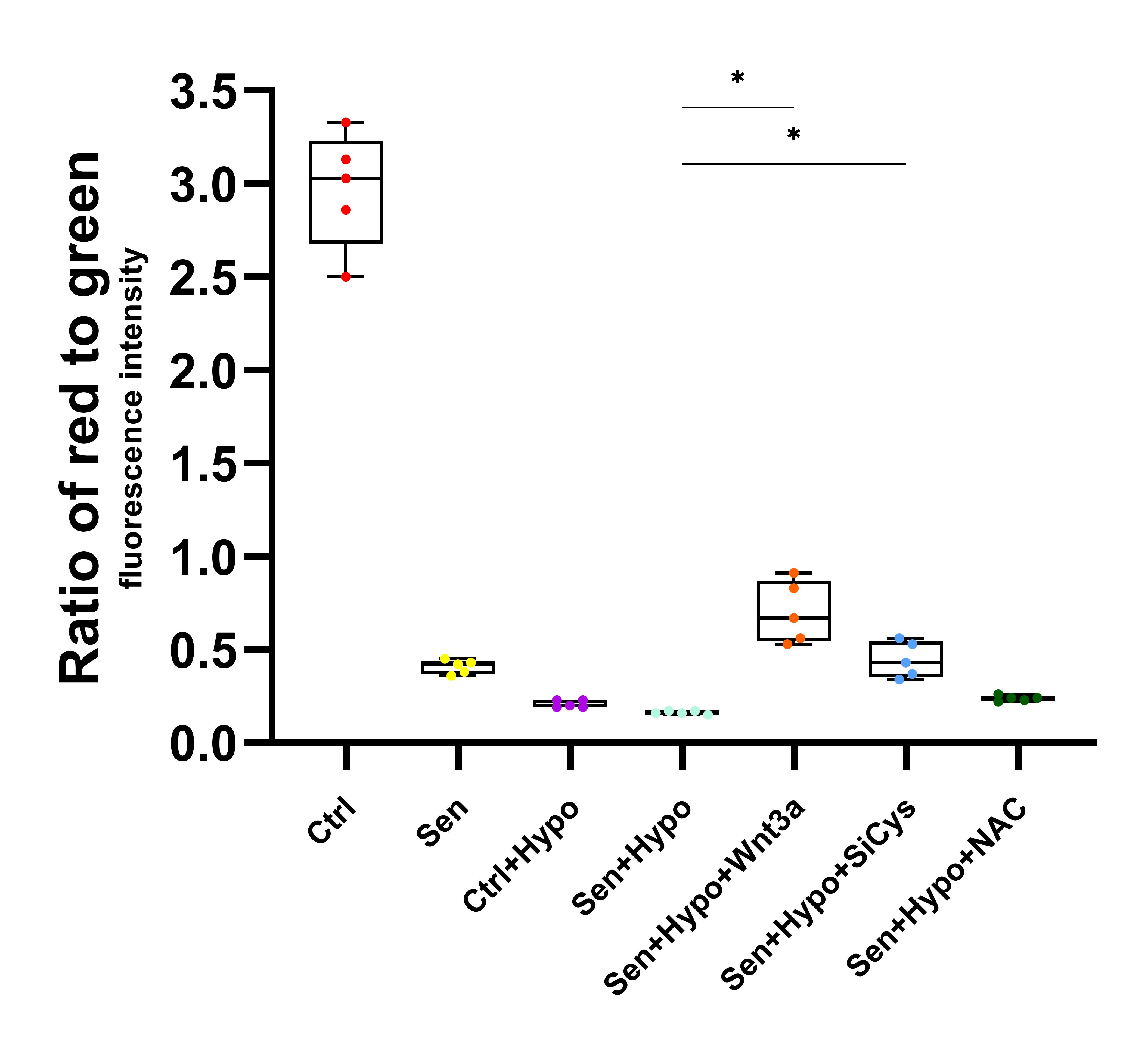

Supplement: Supplementary file 3 [file DataSheet1.ZIP › Original source data 1/Prism data and figures/FIGURE3.E.jpg]

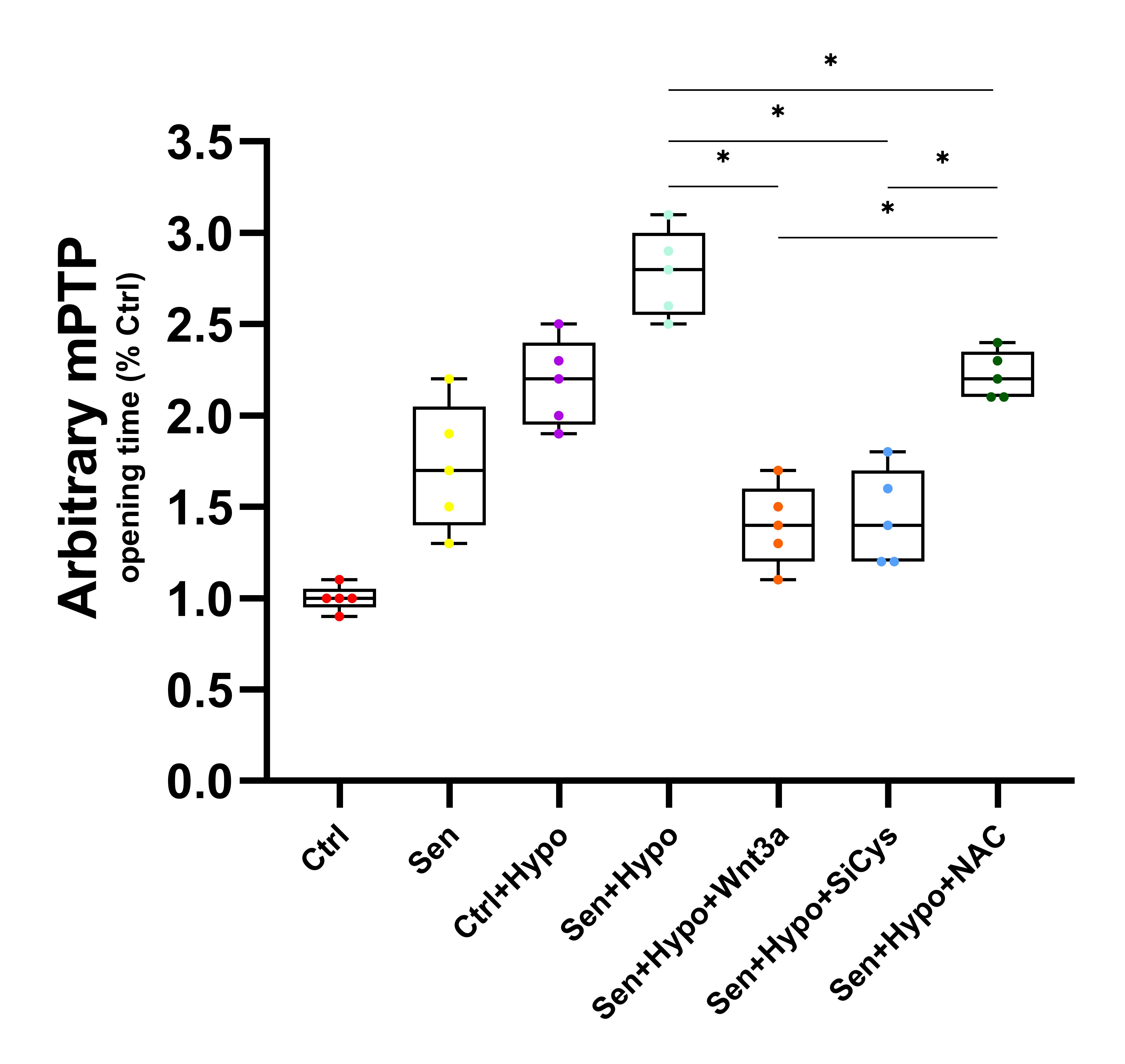

Supplement: Supplementary file 3 [file DataSheet1.ZIP › Original source data 1/Prism data and figures/FIGURE3.F.jpg]

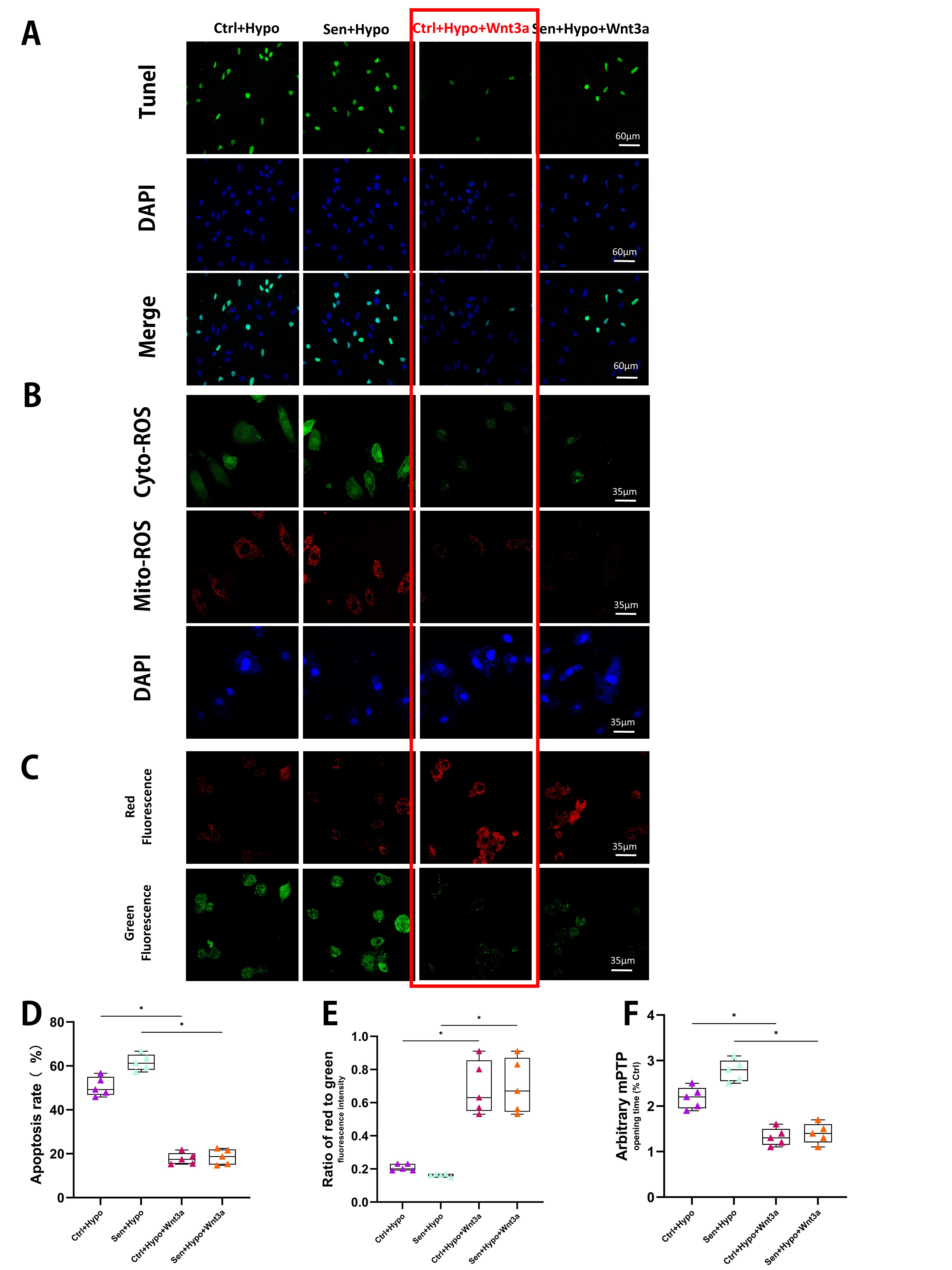

Supplement: Supplementary file 4 [file DataSheet6.ZIP › additional materials to Reviewer #1/addtion01.jpg]

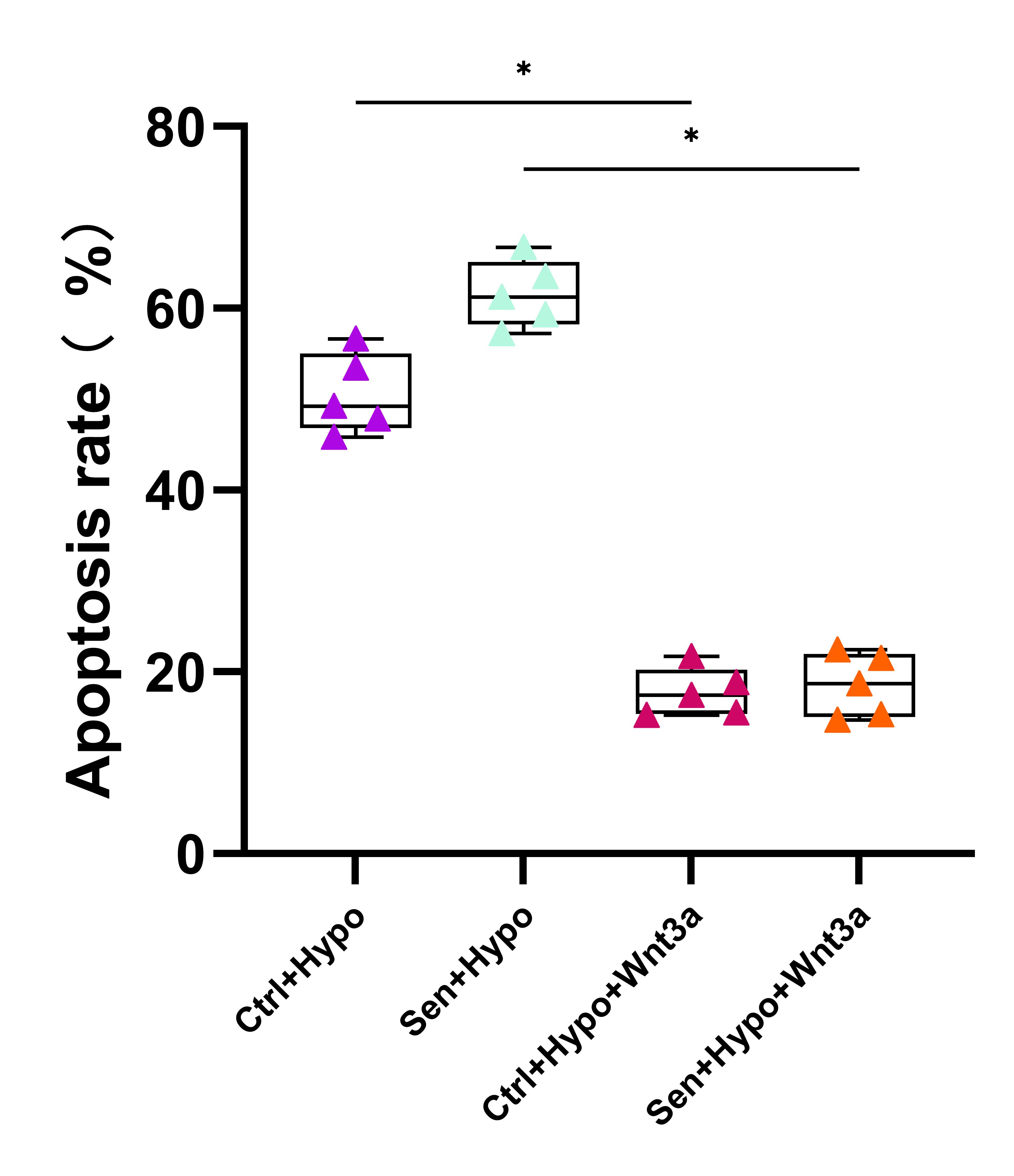

Supplement: Supplementary file 4 [file DataSheet6.ZIP › additional materials to Reviewer #1/apoptosis.jpg]

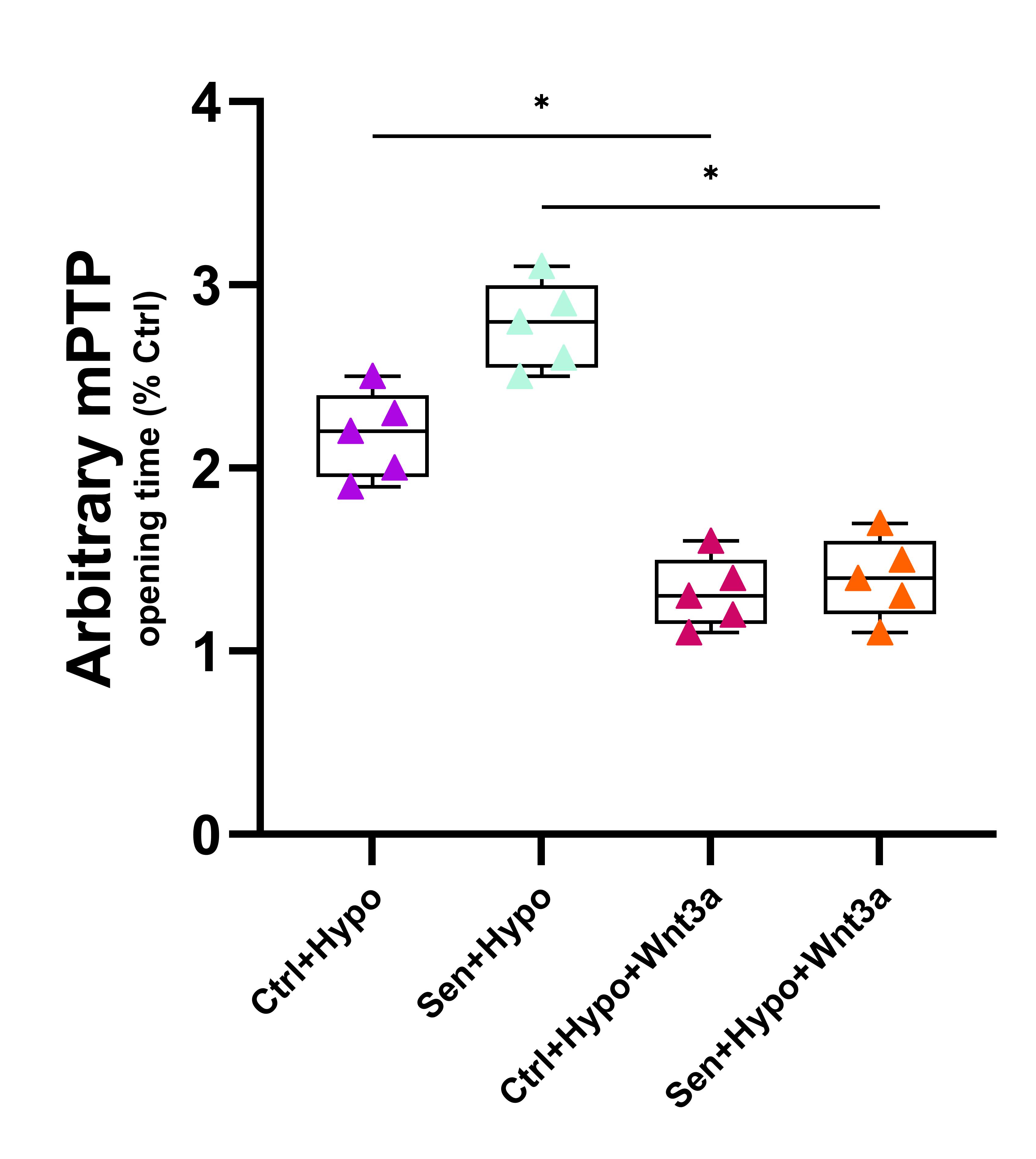

Supplement: Supplementary file 4 [file DataSheet6.ZIP › additional materials to Reviewer #1/mPTP.jpg]

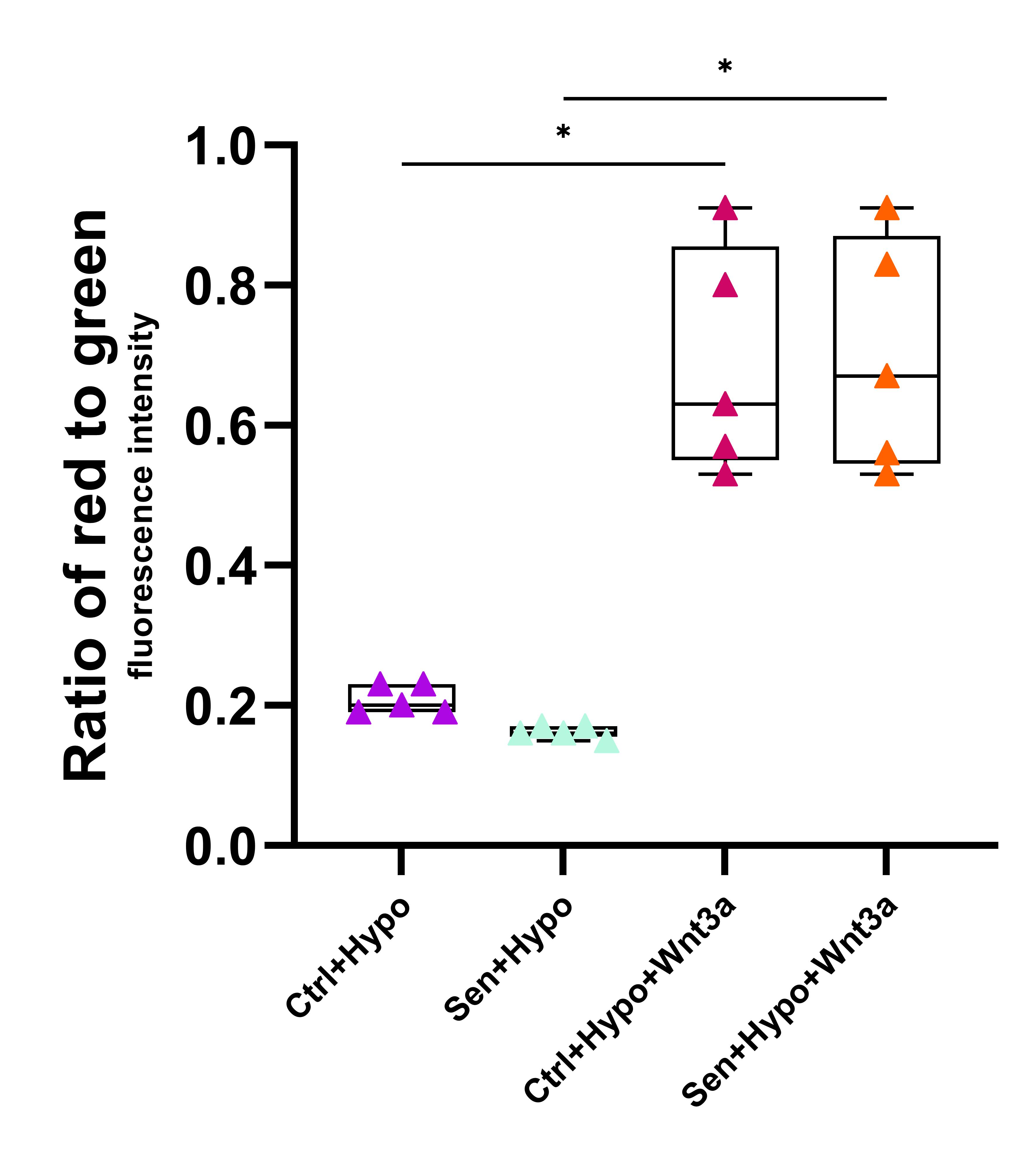

Supplement: Supplementary file 4 [file DataSheet6.ZIP › additional materials to Reviewer #1/red to green.jpg]

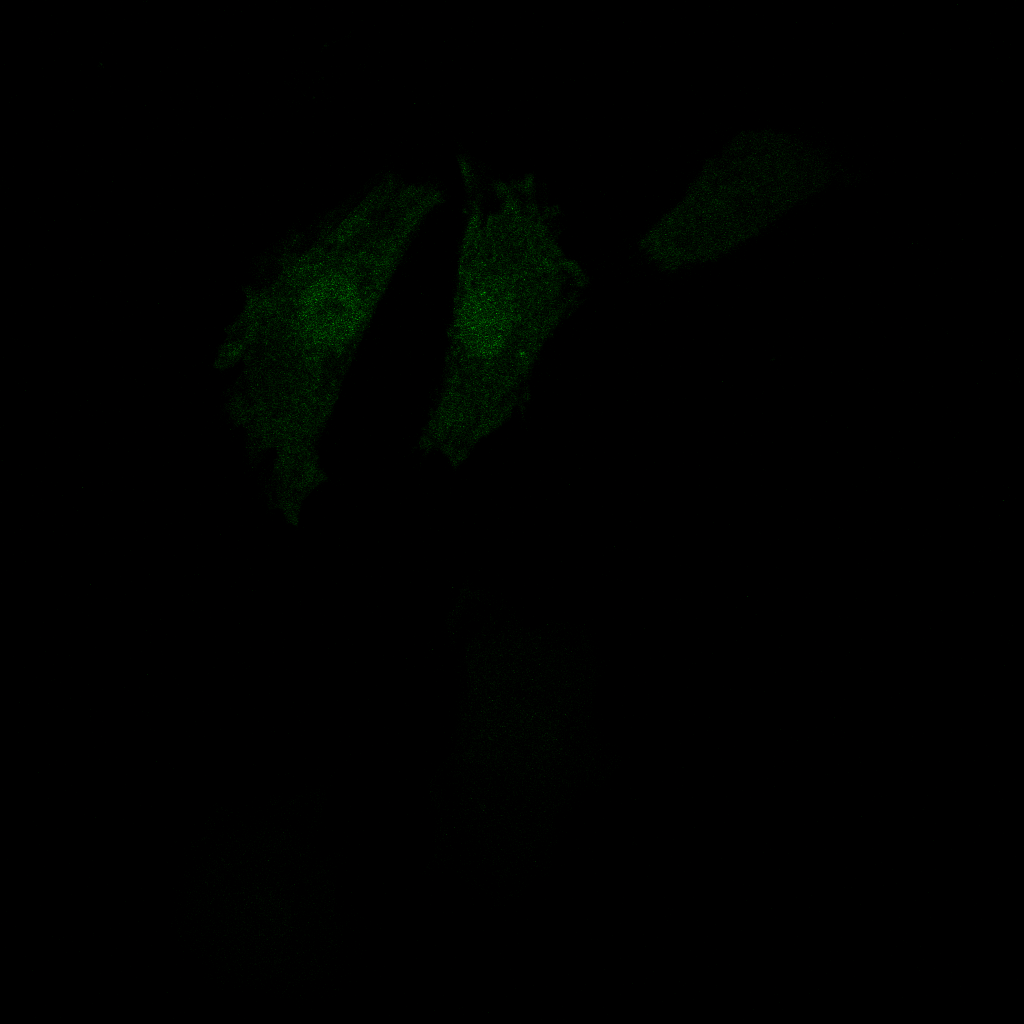

Supplement: Supplementary file 5 [file DataSheet2.ZIP › Original source data 2/FIGURE3.B ROS/Ctrl/Cyto-ROS.tif]

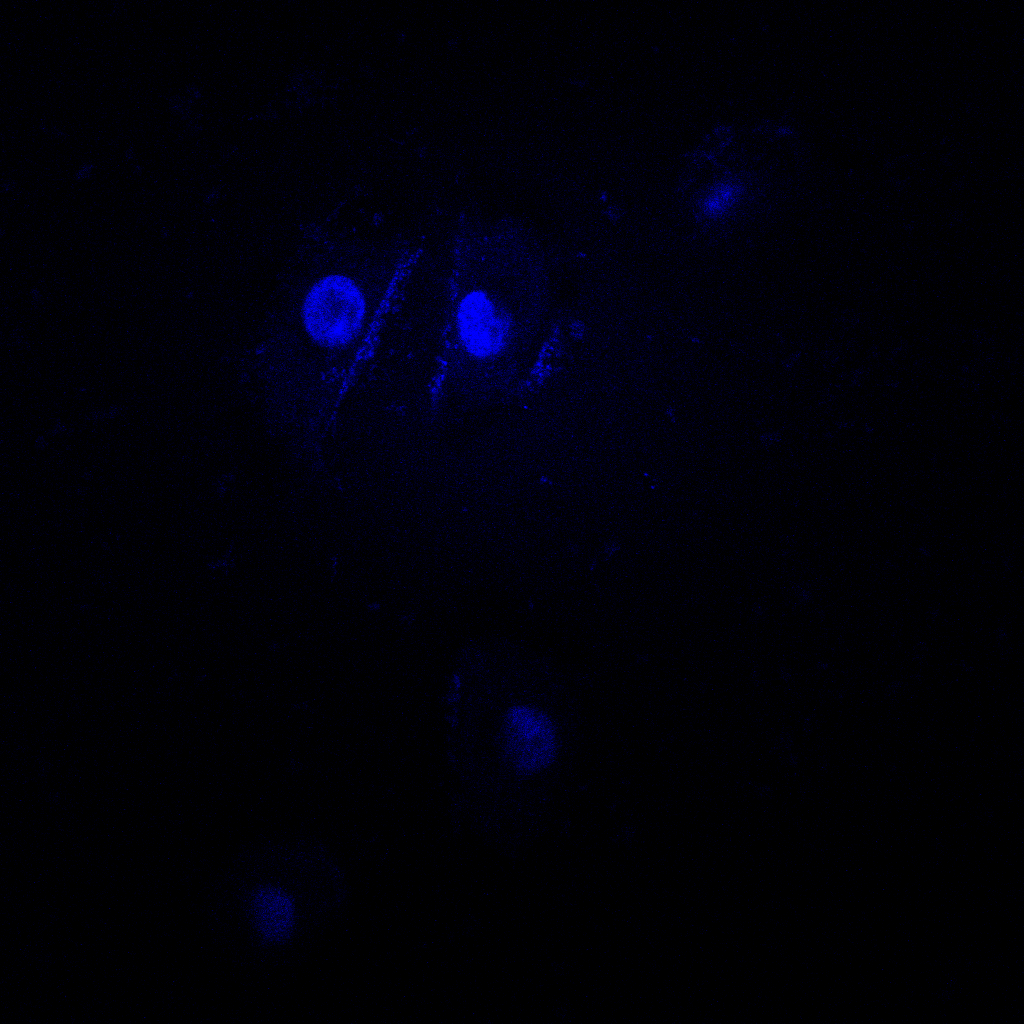

Supplement: Supplementary file 5 [file DataSheet2.ZIP › Original source data 2/FIGURE3.B ROS/Ctrl/DAPI.tif]

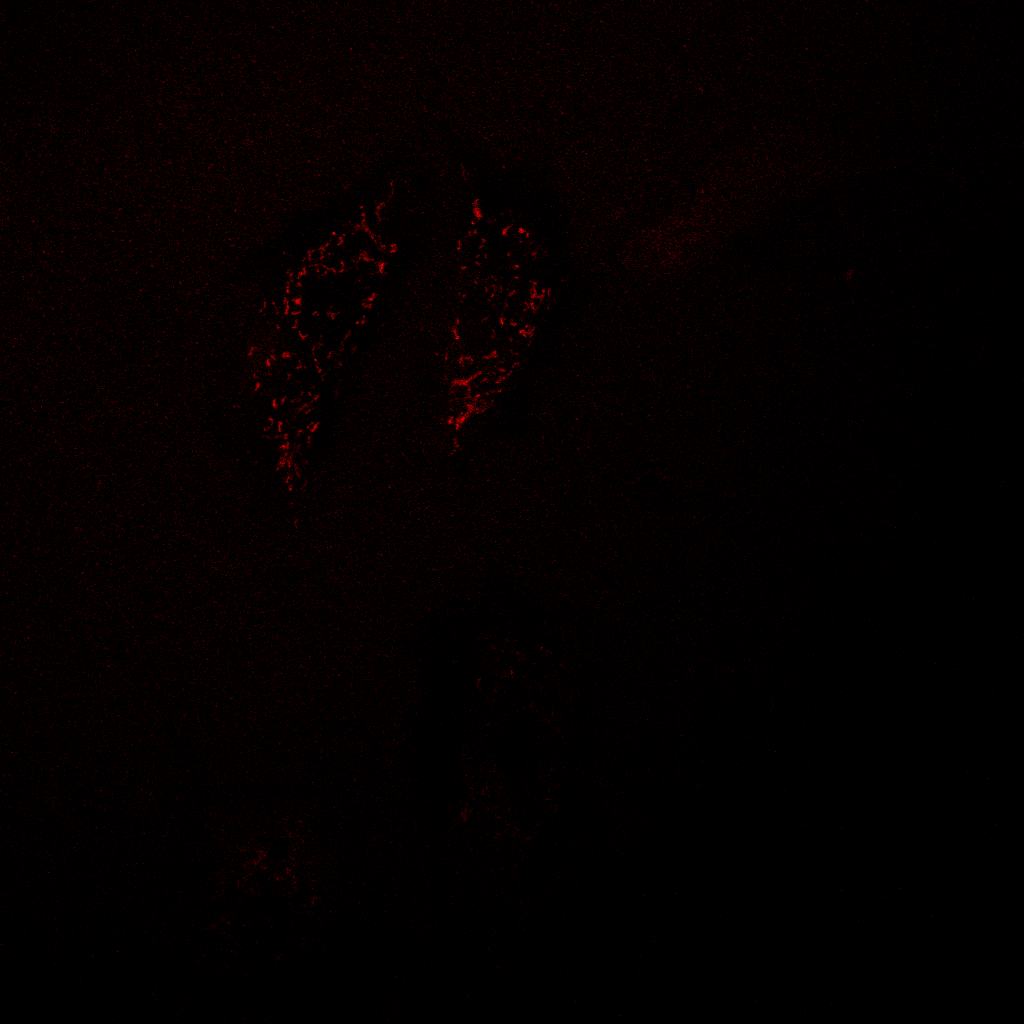

Supplement: Supplementary file 5 [file DataSheet2.ZIP › Original source data 2/FIGURE3.B ROS/Ctrl/Mito-ROS.tif]

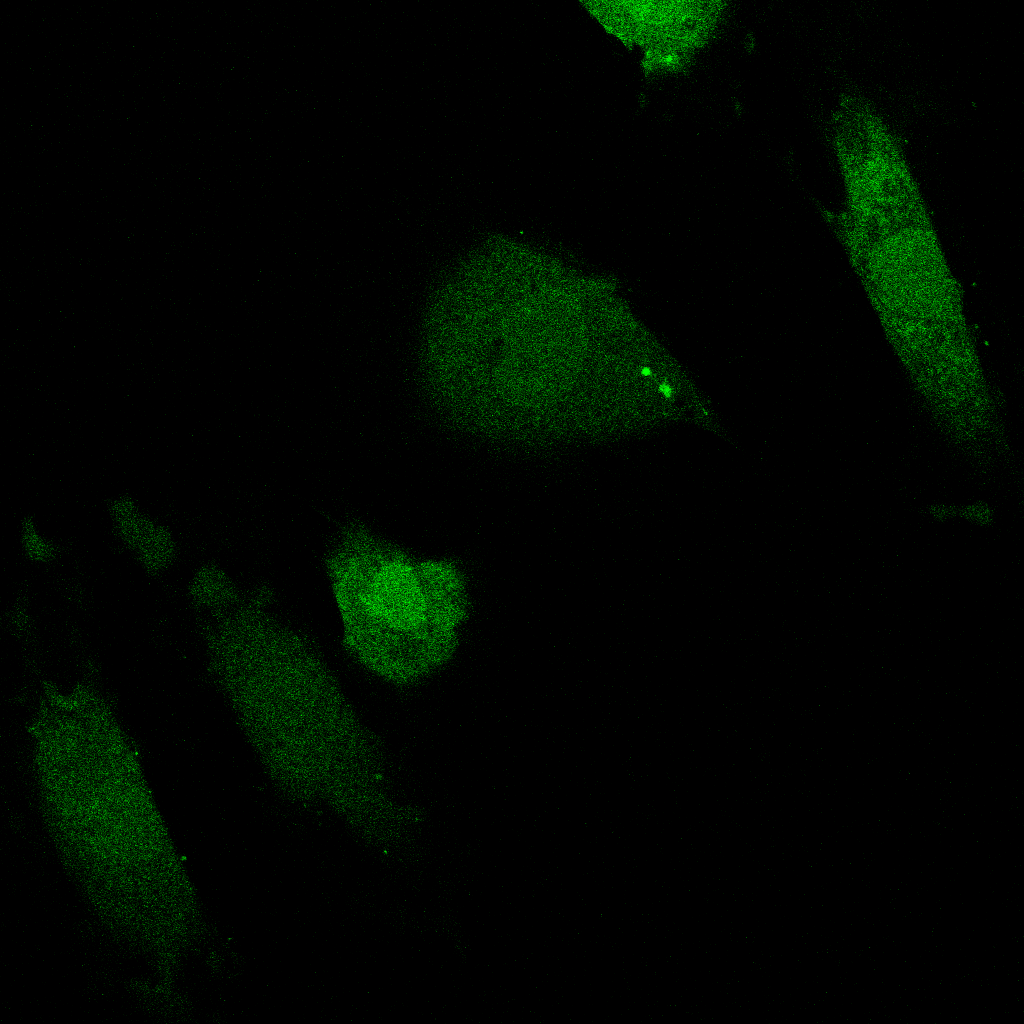

Supplement: Supplementary file 5 [file DataSheet2.ZIP › Original source data 2/FIGURE3.B ROS/Ctrl+Ischemia/Cyto-ROS.tif]

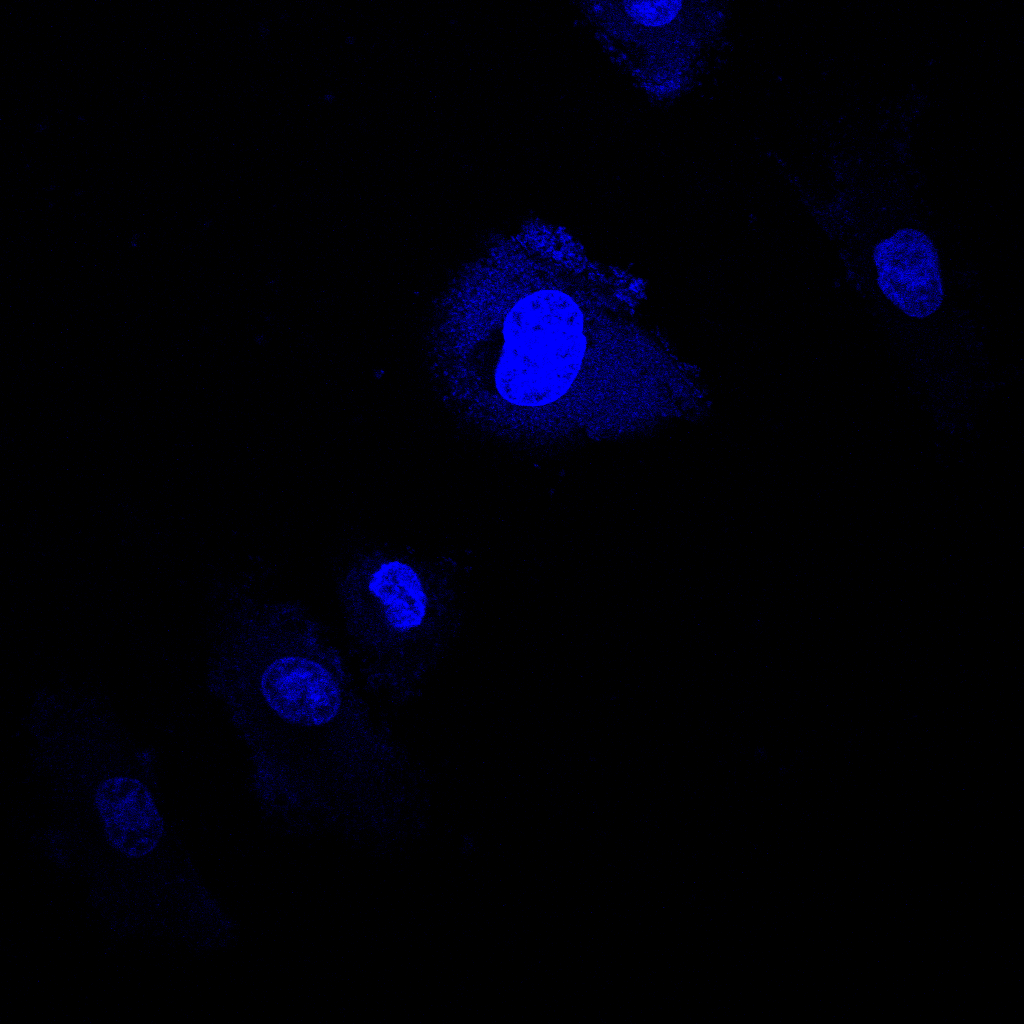

Supplement: Supplementary file 5 [file DataSheet2.ZIP › Original source data 2/FIGURE3.B ROS/Ctrl+Ischemia/DAPI.tif]

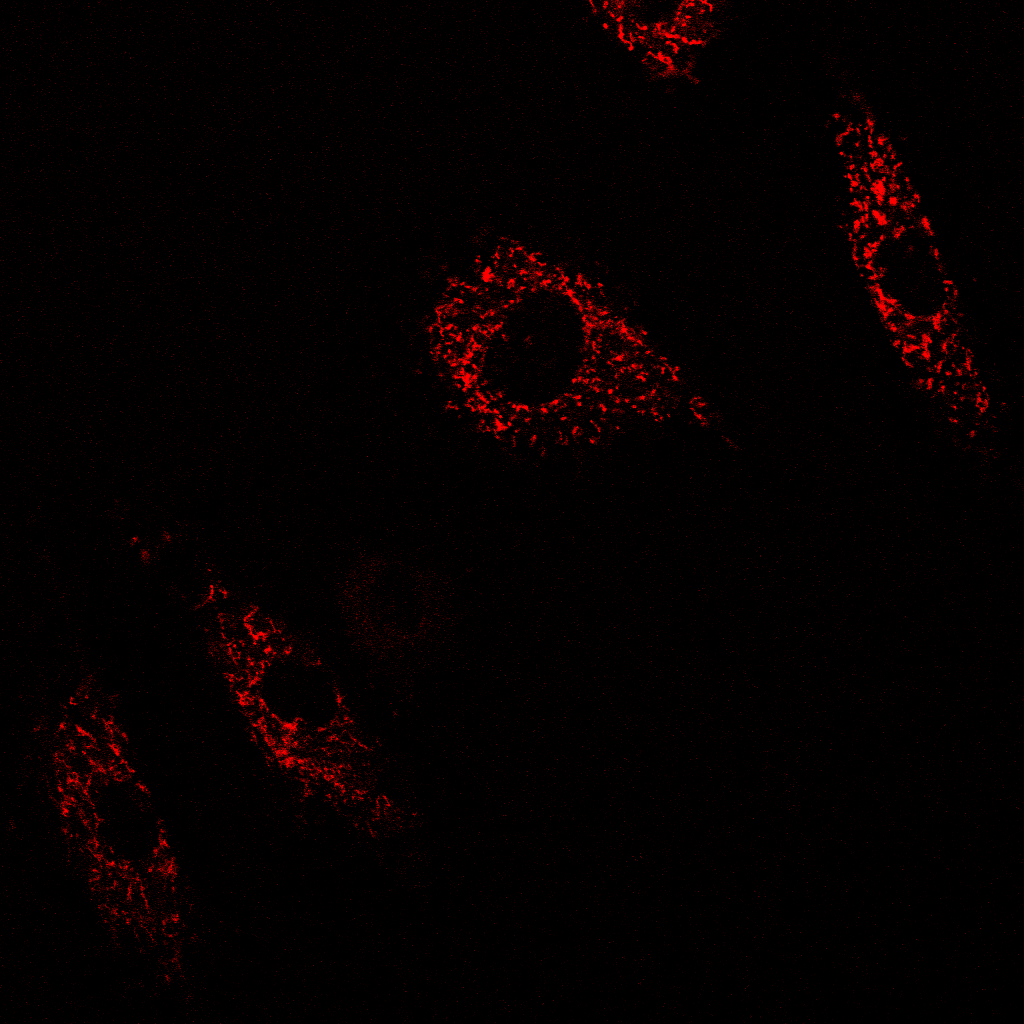

Supplement: Supplementary file 5 [file DataSheet2.ZIP › Original source data 2/FIGURE3.B ROS/Ctrl+Ischemia/Mito-ROS.tif]

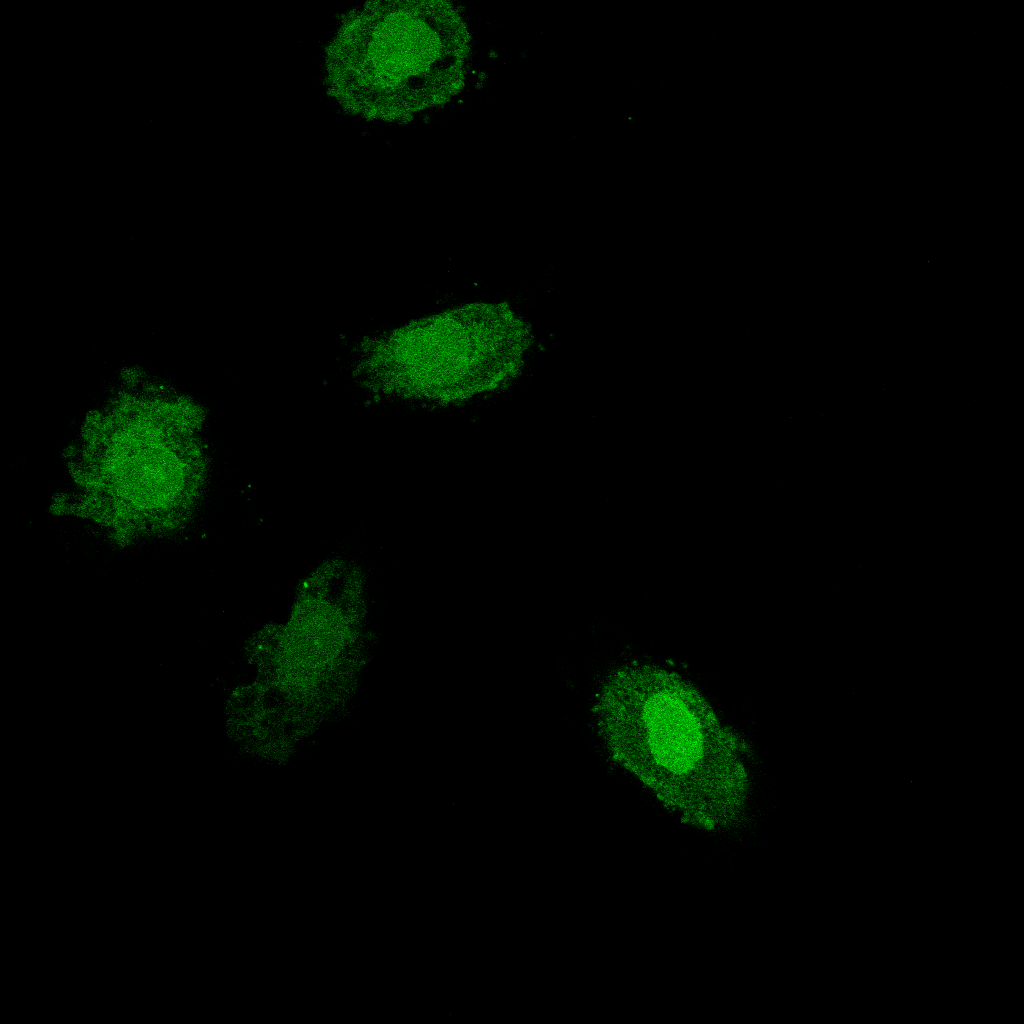

Supplement: Supplementary file 5 [file DataSheet2.ZIP › Original source data 2/FIGURE3.B ROS/Sen/Cyto-ROS.tif]

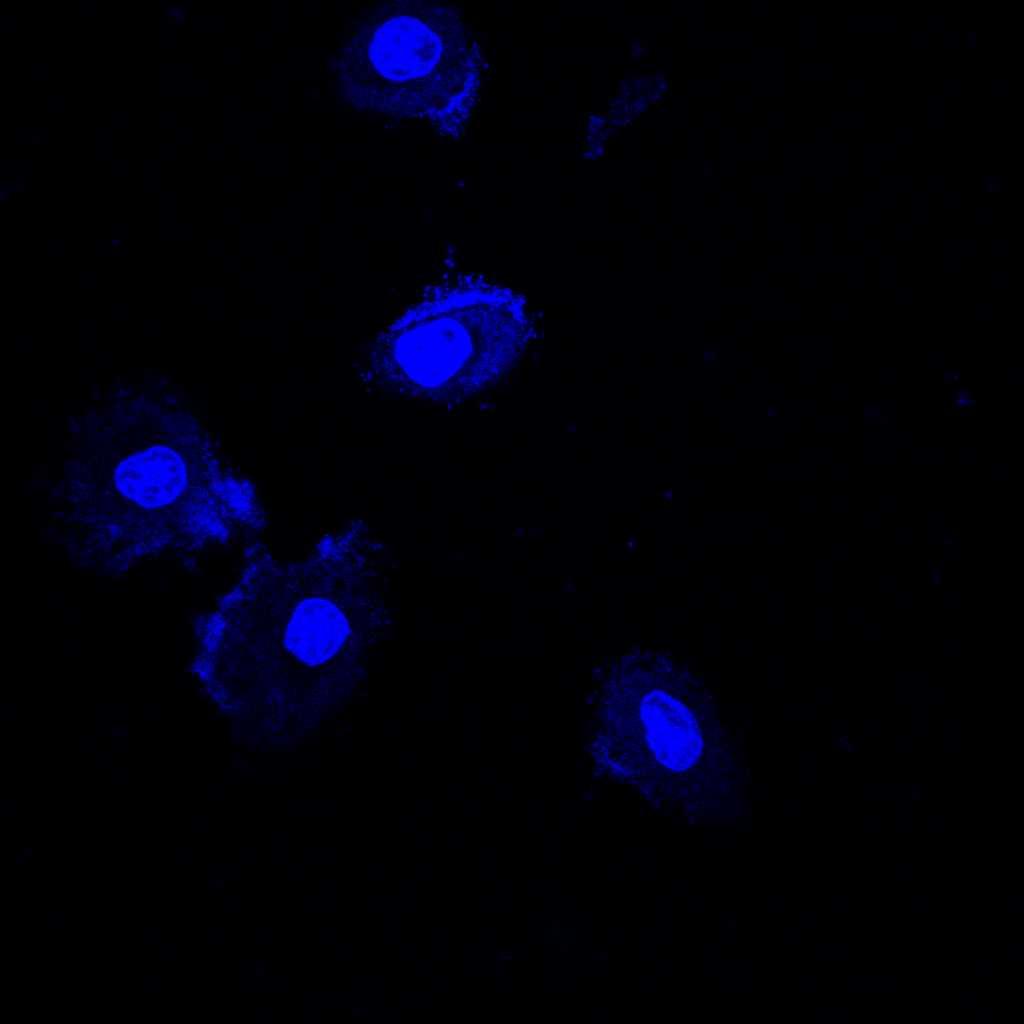

Supplement: Supplementary file 5 [file DataSheet2.ZIP › Original source data 2/FIGURE3.B ROS/Sen/DAPI.tif]

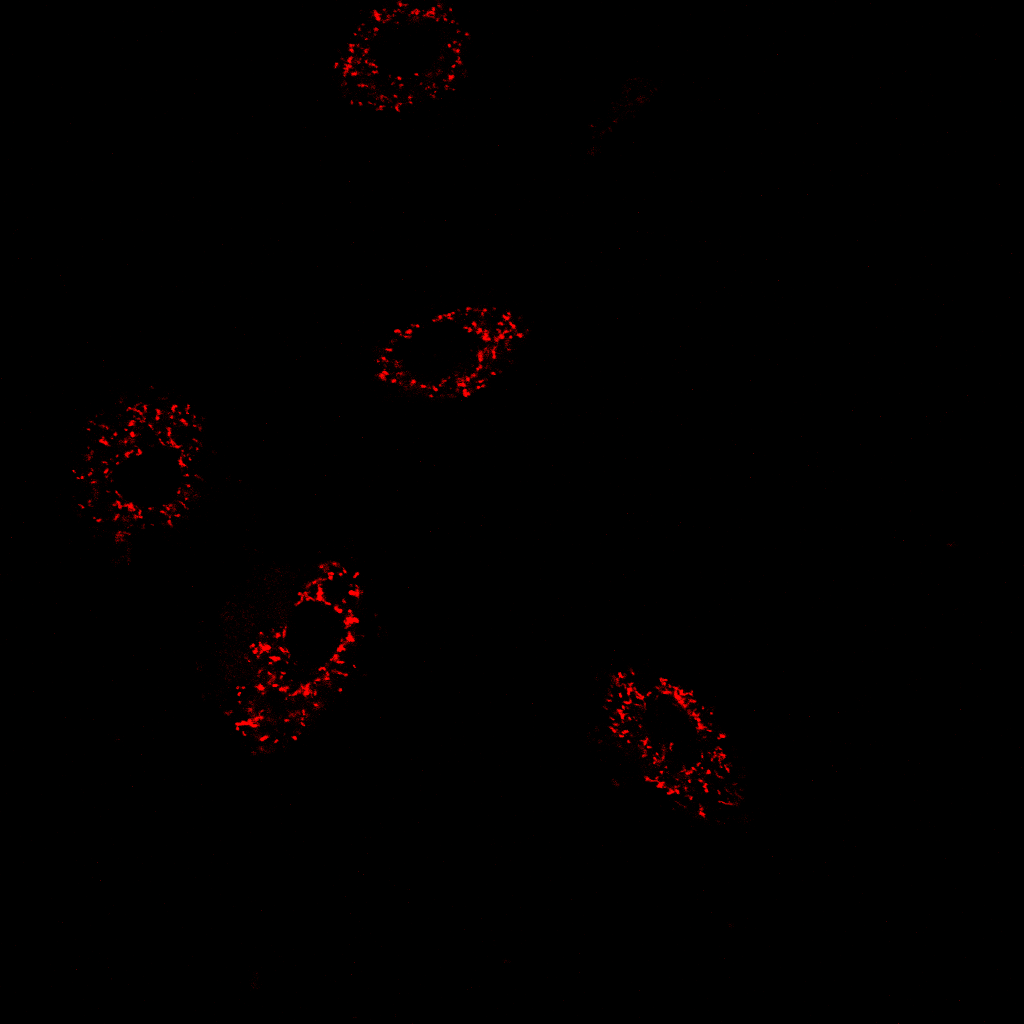

Supplement: Supplementary file 5 [file DataSheet2.ZIP › Original source data 2/FIGURE3.B ROS/Sen/Mito-ROS.tif]

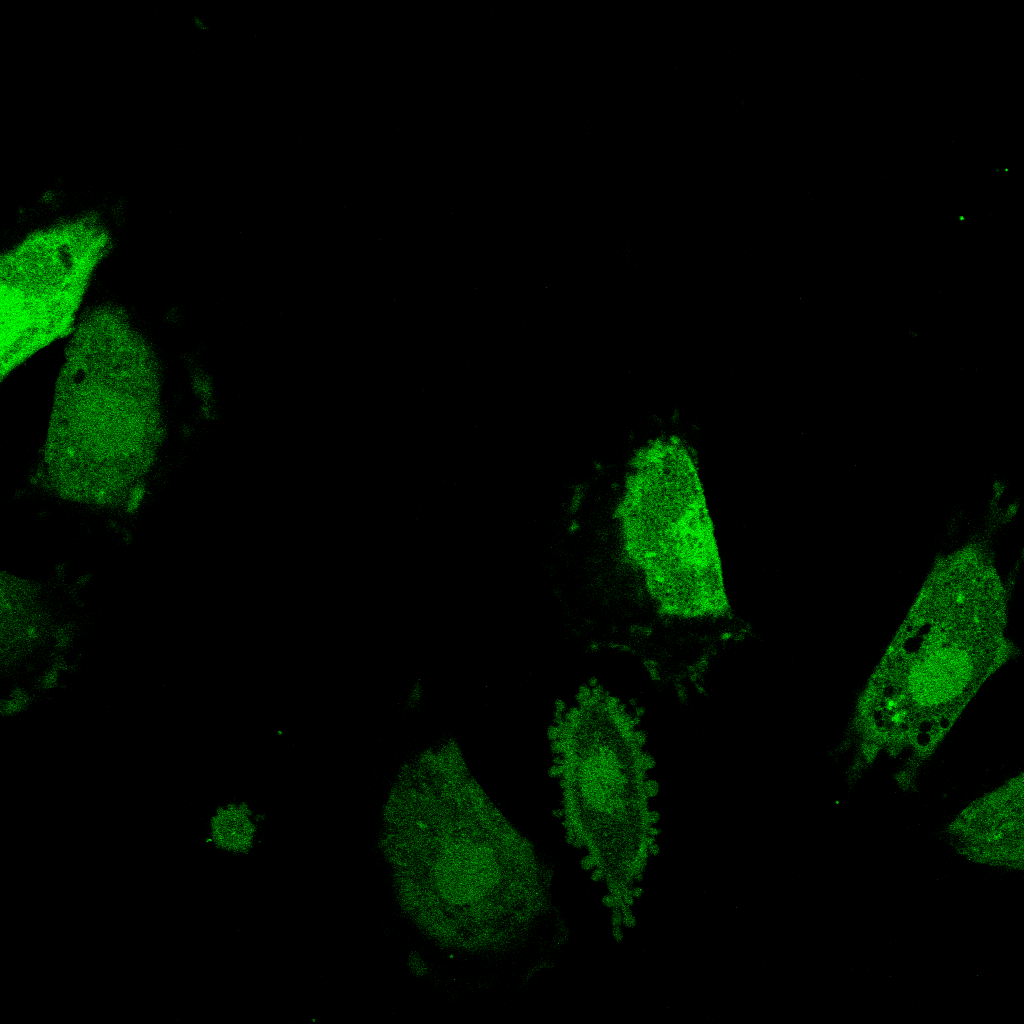

Supplement: Supplementary file 5 [file DataSheet2.ZIP › Original source data 2/FIGURE3.B ROS/Sen+Ischemia/Cyto-ROS.tif]

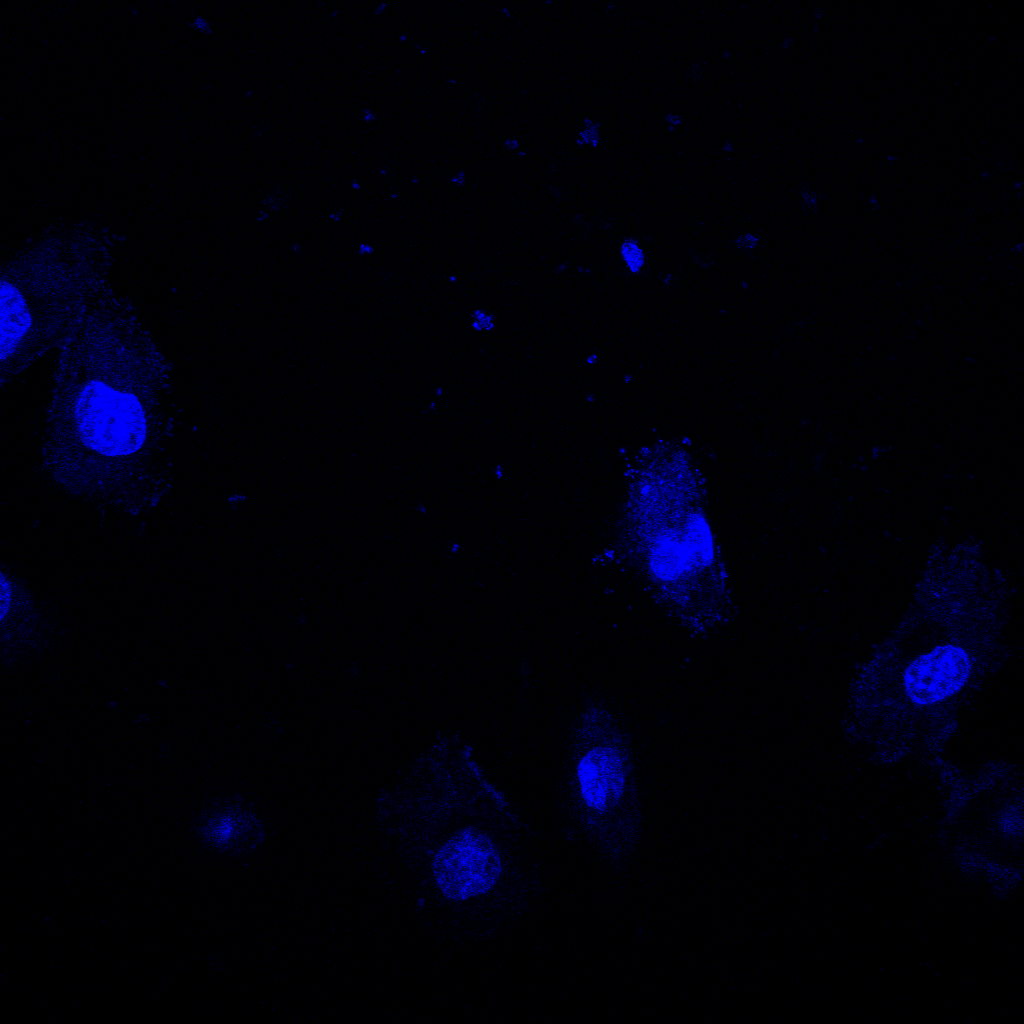

Supplement: Supplementary file 5 [file DataSheet2.ZIP › Original source data 2/FIGURE3.B ROS/Sen+Ischemia/DAPI.tif]

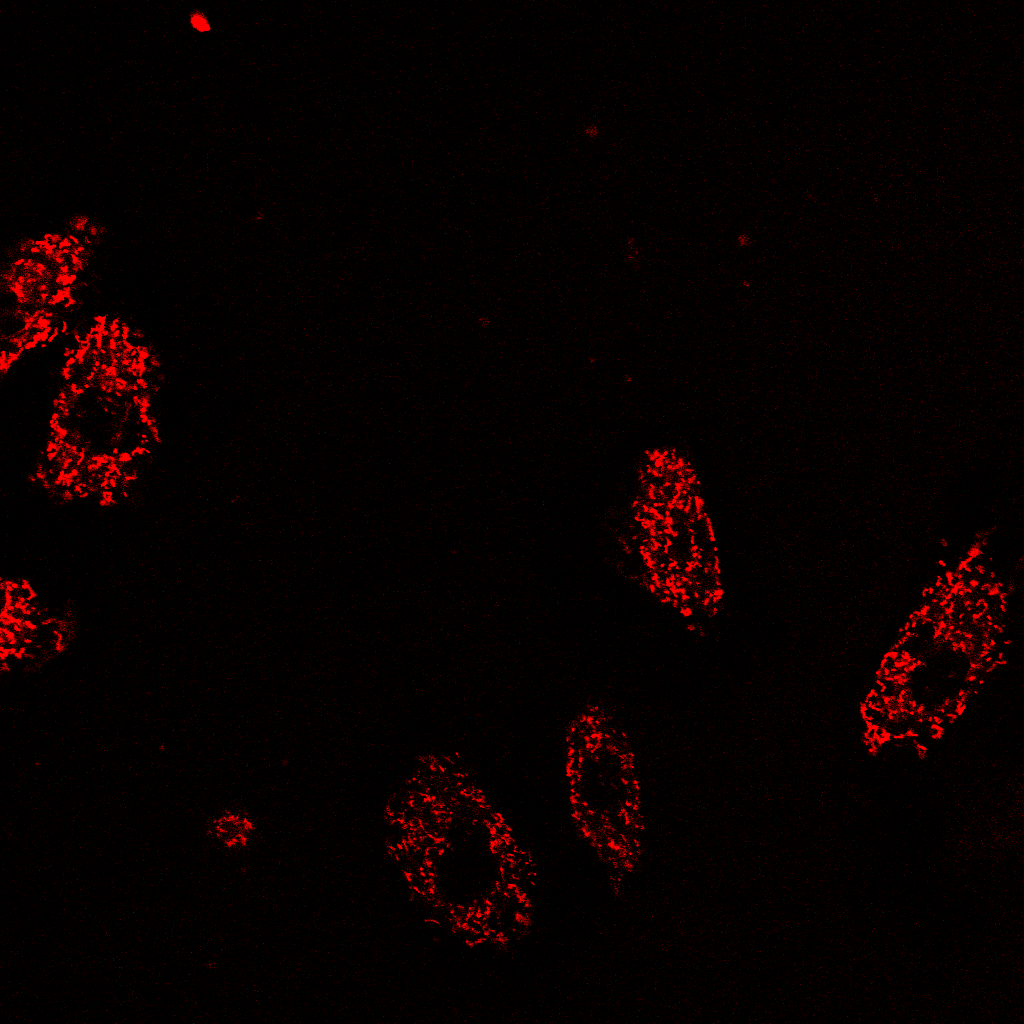

Supplement: Supplementary file 5 [file DataSheet2.ZIP › Original source data 2/FIGURE3.B ROS/Sen+Ischemia/Mito-ROS.tif]

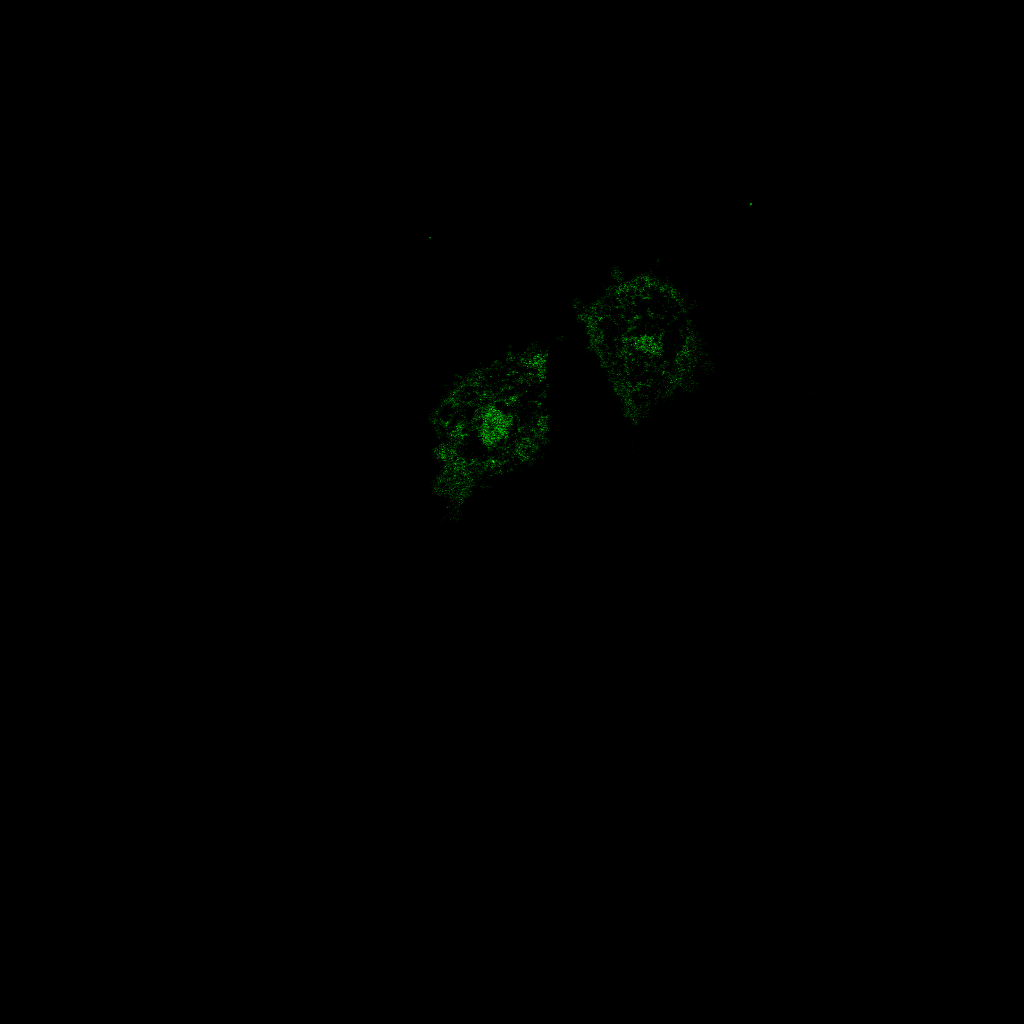

Supplement: Supplementary file 5 [file DataSheet2.ZIP › Original source data 2/FIGURE3.B ROS/Sen+Ischemia+NAC/Cyto-ROS.tif]

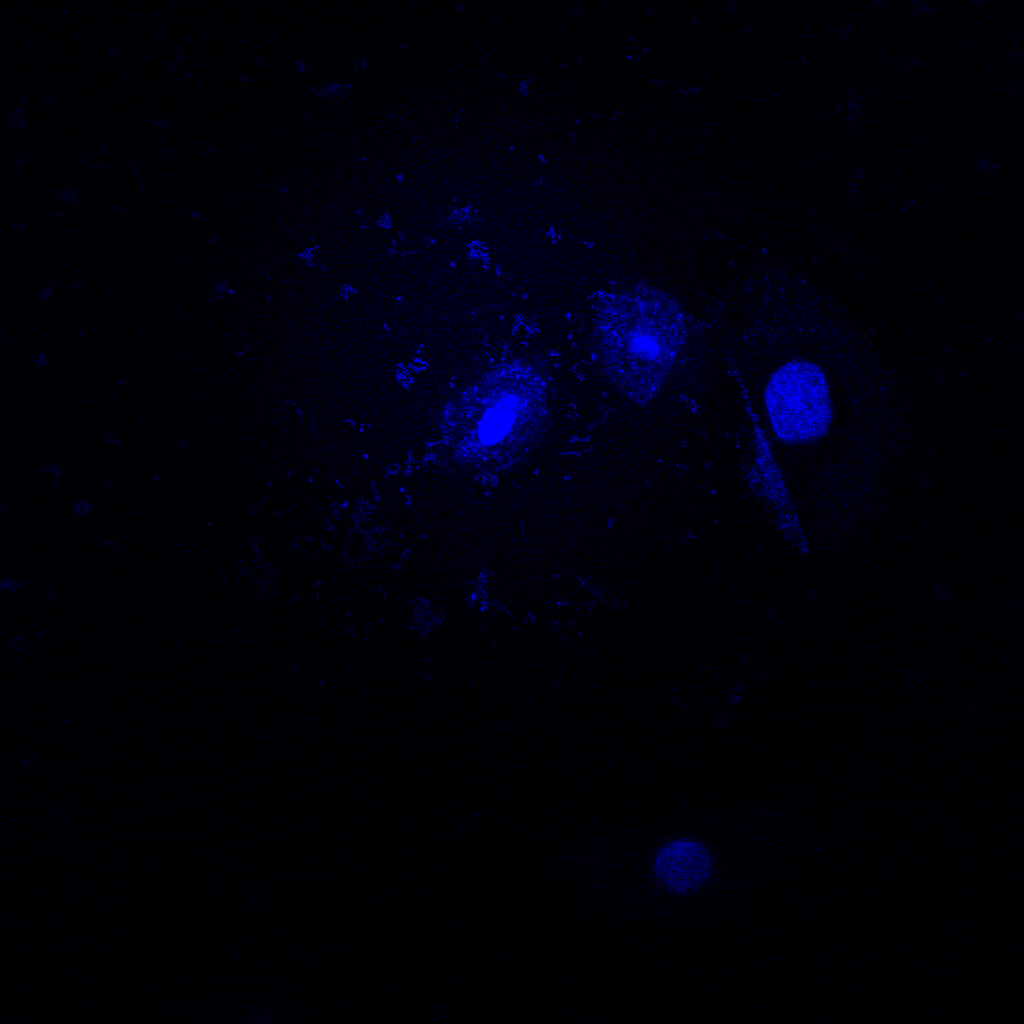

Supplement: Supplementary file 5 [file DataSheet2.ZIP › Original source data 2/FIGURE3.B ROS/Sen+Ischemia+NAC/DAPI.tif]

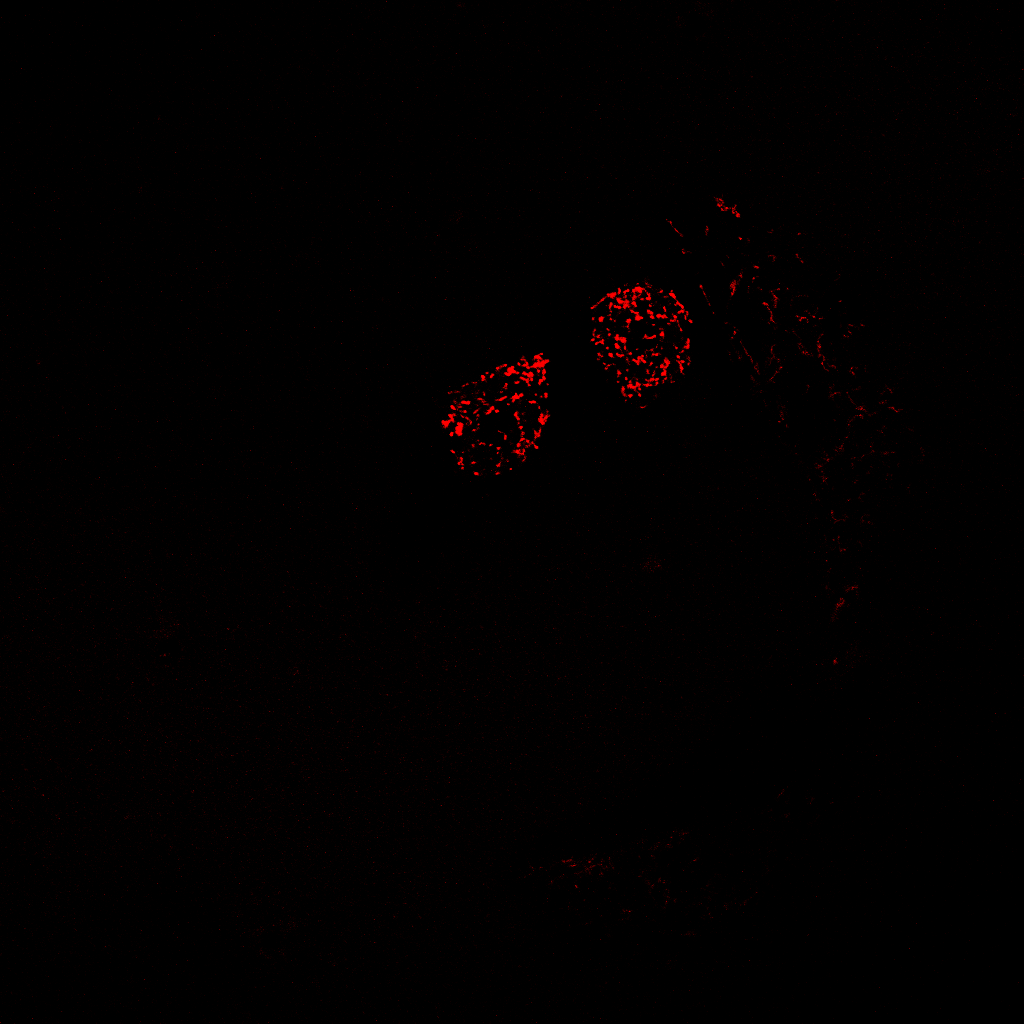

Supplement: Supplementary file 5 [file DataSheet2.ZIP › Original source data 2/FIGURE3.B ROS/Sen+Ischemia+NAC/Mito-ROS.tif]

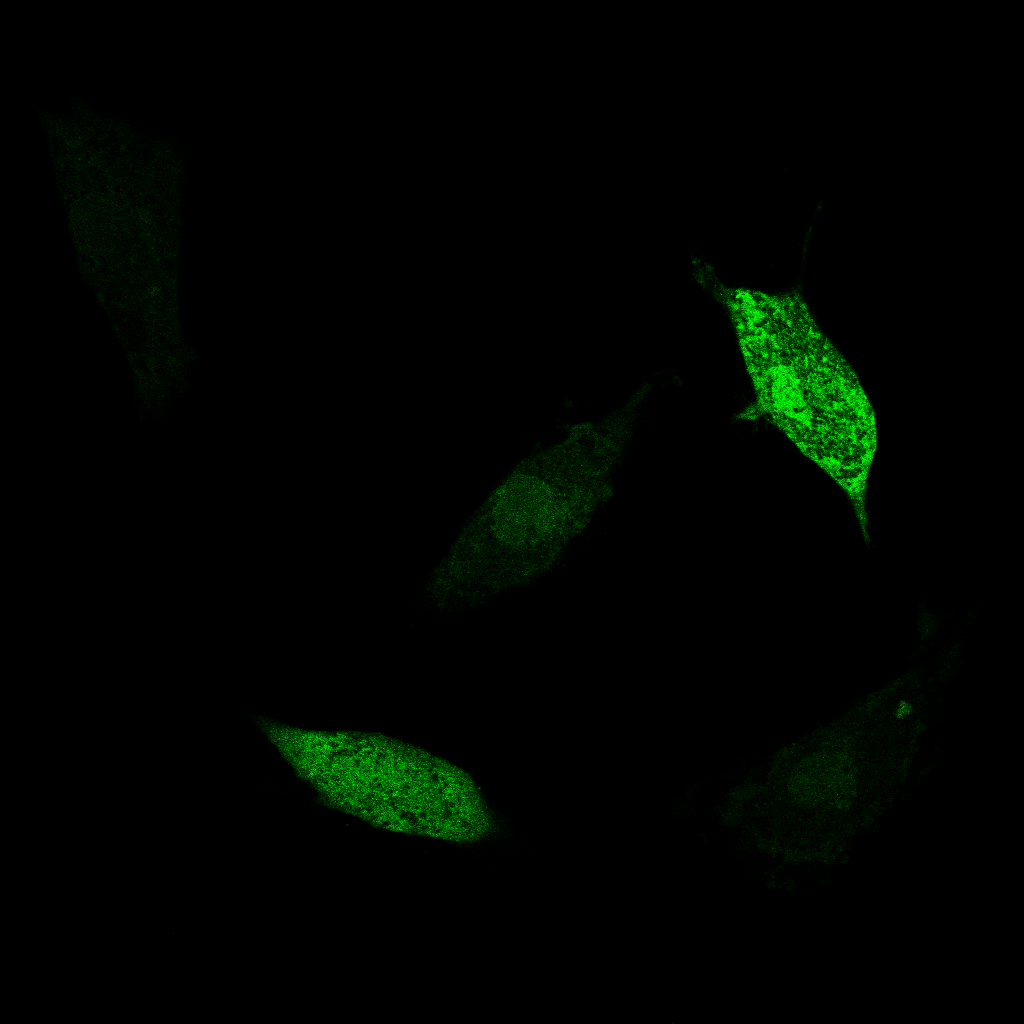

Supplement: Supplementary file 5 [file DataSheet2.ZIP › Original source data 2/FIGURE3.B ROS/Sen+Ischemia+SiCys/Cyto-ROS.tif]

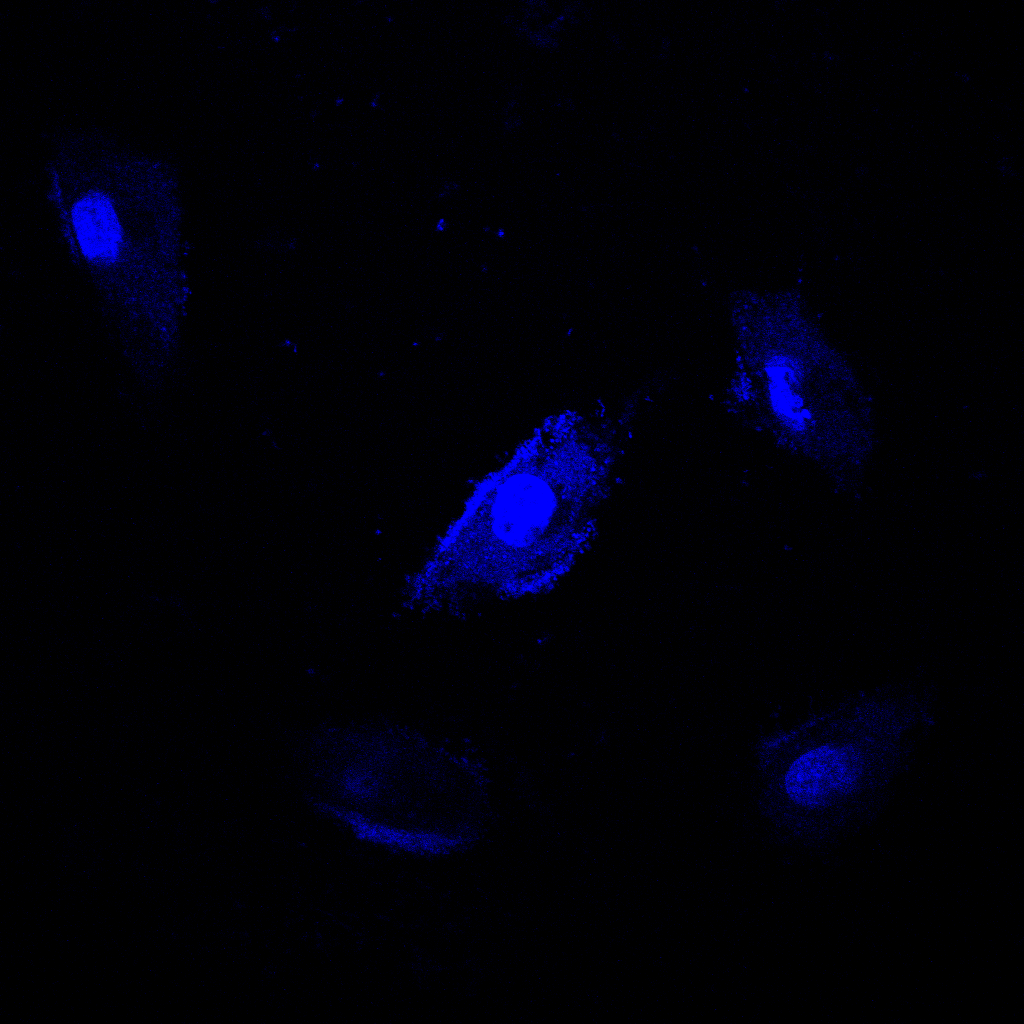

Supplement: Supplementary file 5 [file DataSheet2.ZIP › Original source data 2/FIGURE3.B ROS/Sen+Ischemia+SiCys/DAPI.tif]

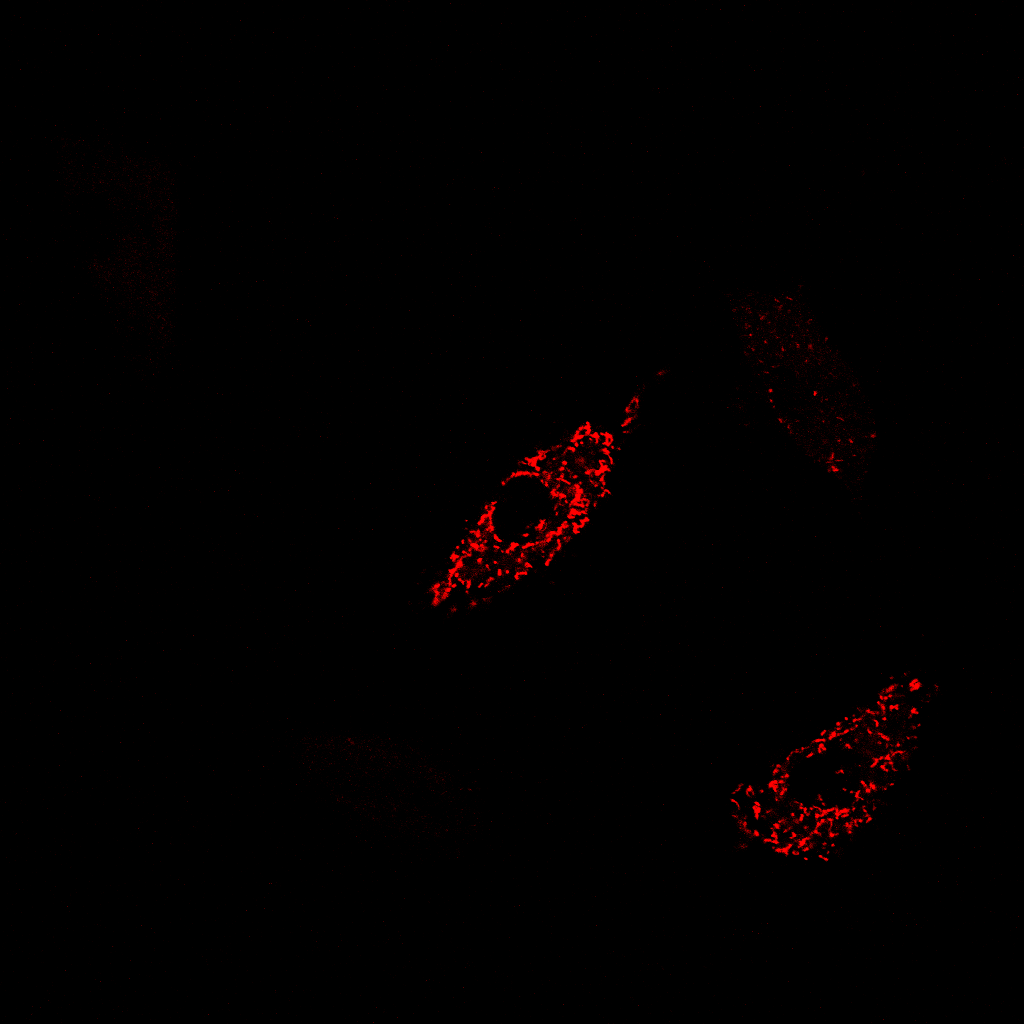

Supplement: Supplementary file 5 [file DataSheet2.ZIP › Original source data 2/FIGURE3.B ROS/Sen+Ischemia+SiCys/Mito-ROS.tif]

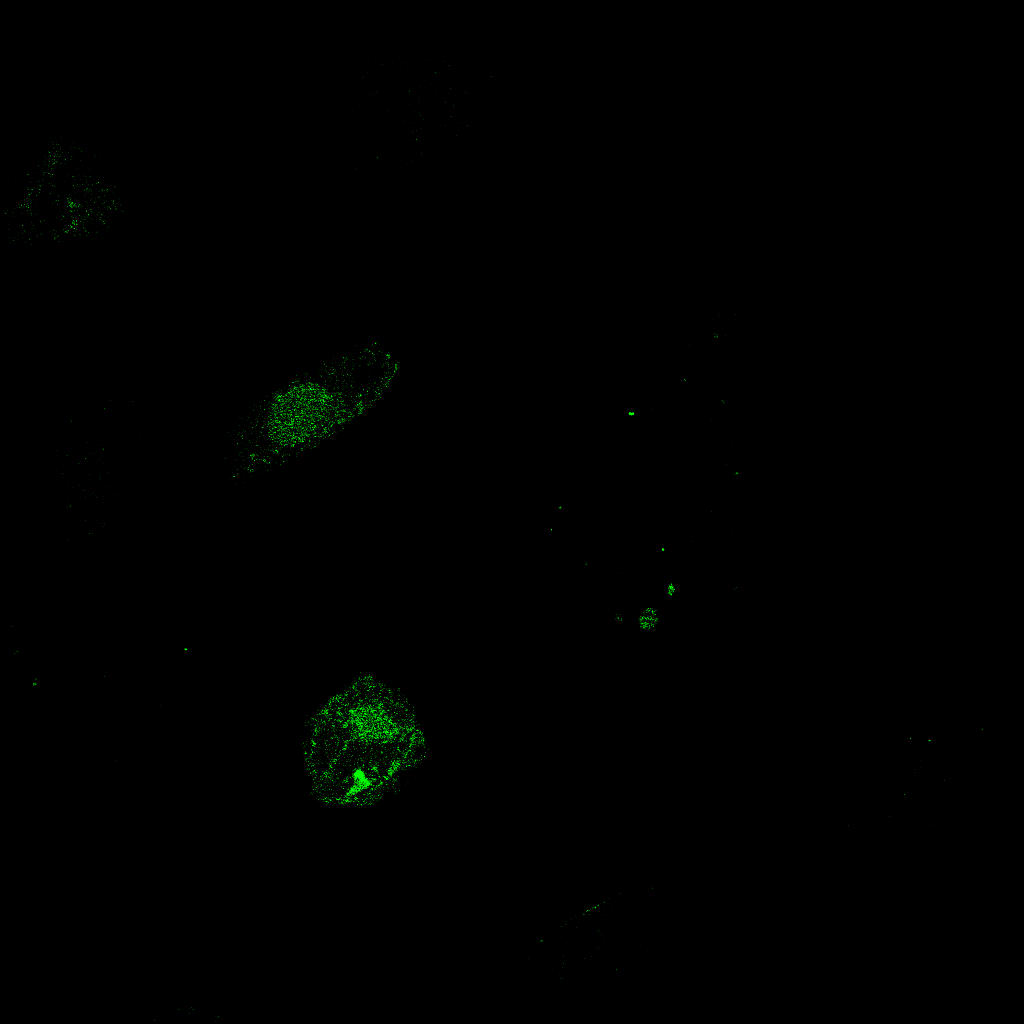

Supplement: Supplementary file 5 [file DataSheet2.ZIP › Original source data 2/FIGURE3.B ROS/Sen+Ischemia+Wnt3a/Cyto-ROS.tif]

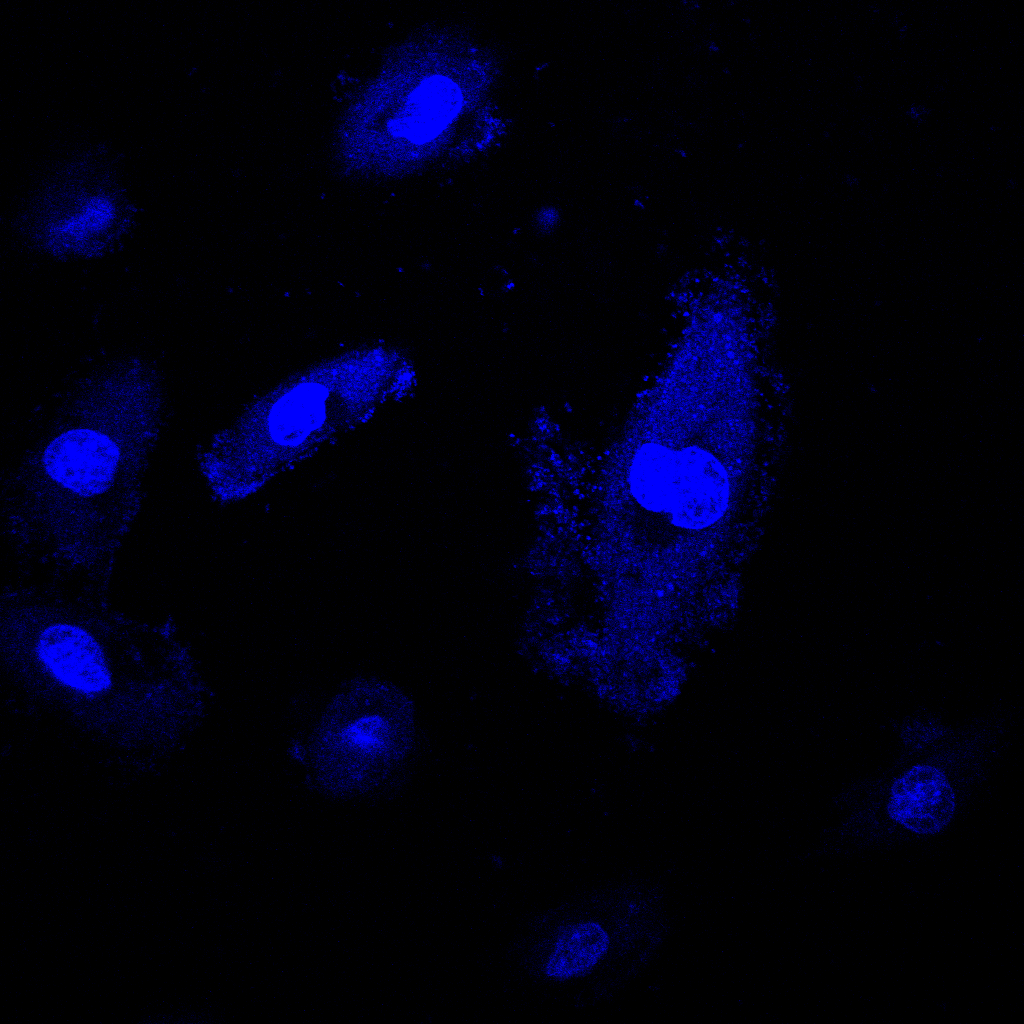

Supplement: Supplementary file 5 [file DataSheet2.ZIP › Original source data 2/FIGURE3.B ROS/Sen+Ischemia+Wnt3a/DAPI.tif]

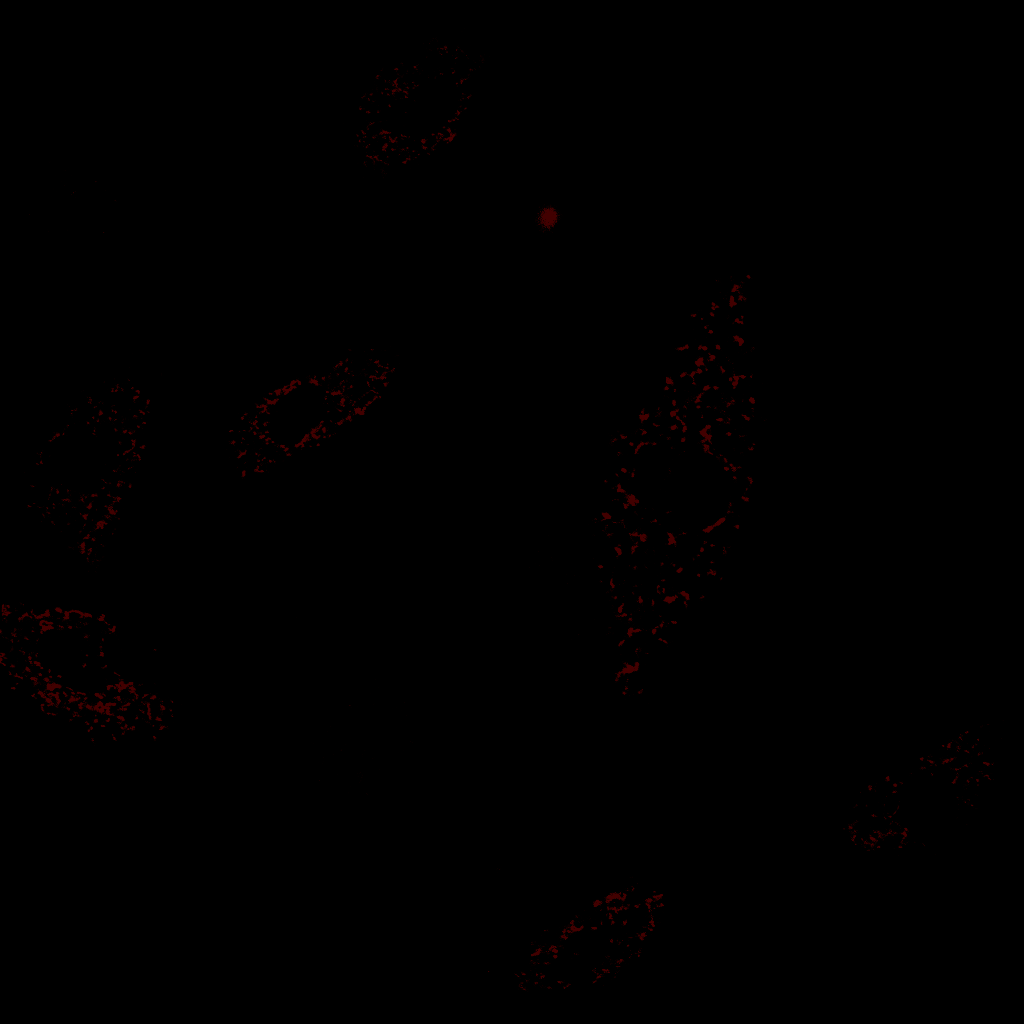

Supplement: Supplementary file 5 [file DataSheet2.ZIP › Original source data 2/FIGURE3.B ROS/Sen+Ischemia+Wnt3a/Mito-ROS.tif]

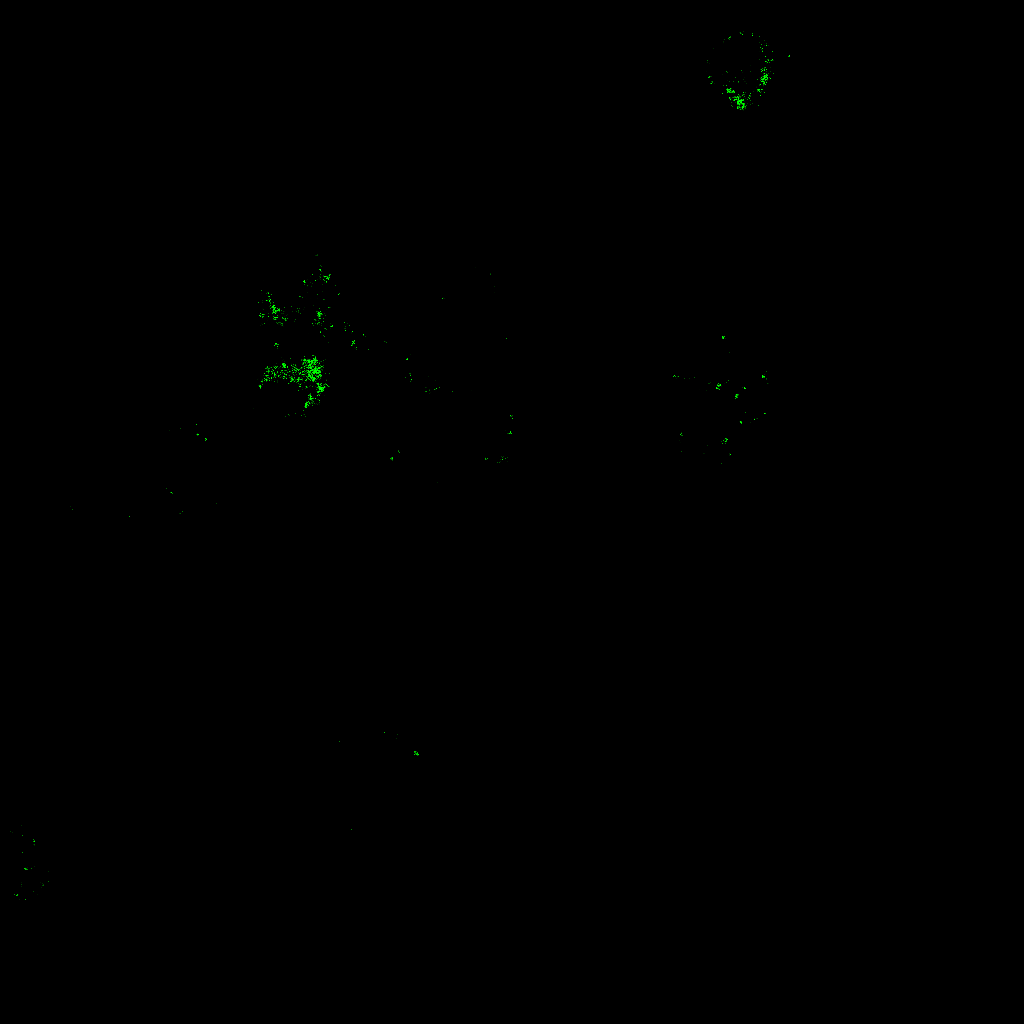

Supplement: Supplementary file 5 [file DataSheet2.ZIP › Original source data 2/FIGURE3.C RED TO GREEN/Ctrl/GREEN.tif]

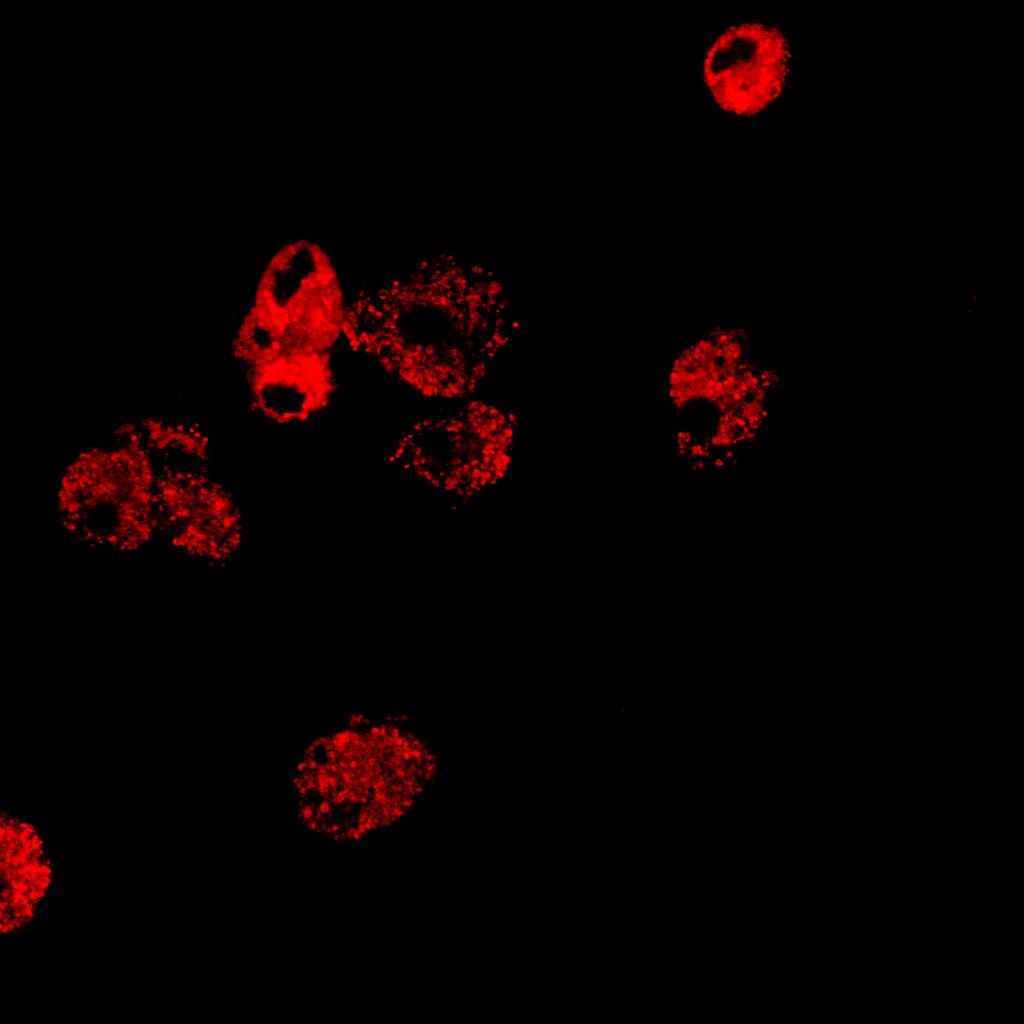

Supplement: Supplementary file 5 [file DataSheet2.ZIP › Original source data 2/FIGURE3.C RED TO GREEN/Ctrl/RED.tif]

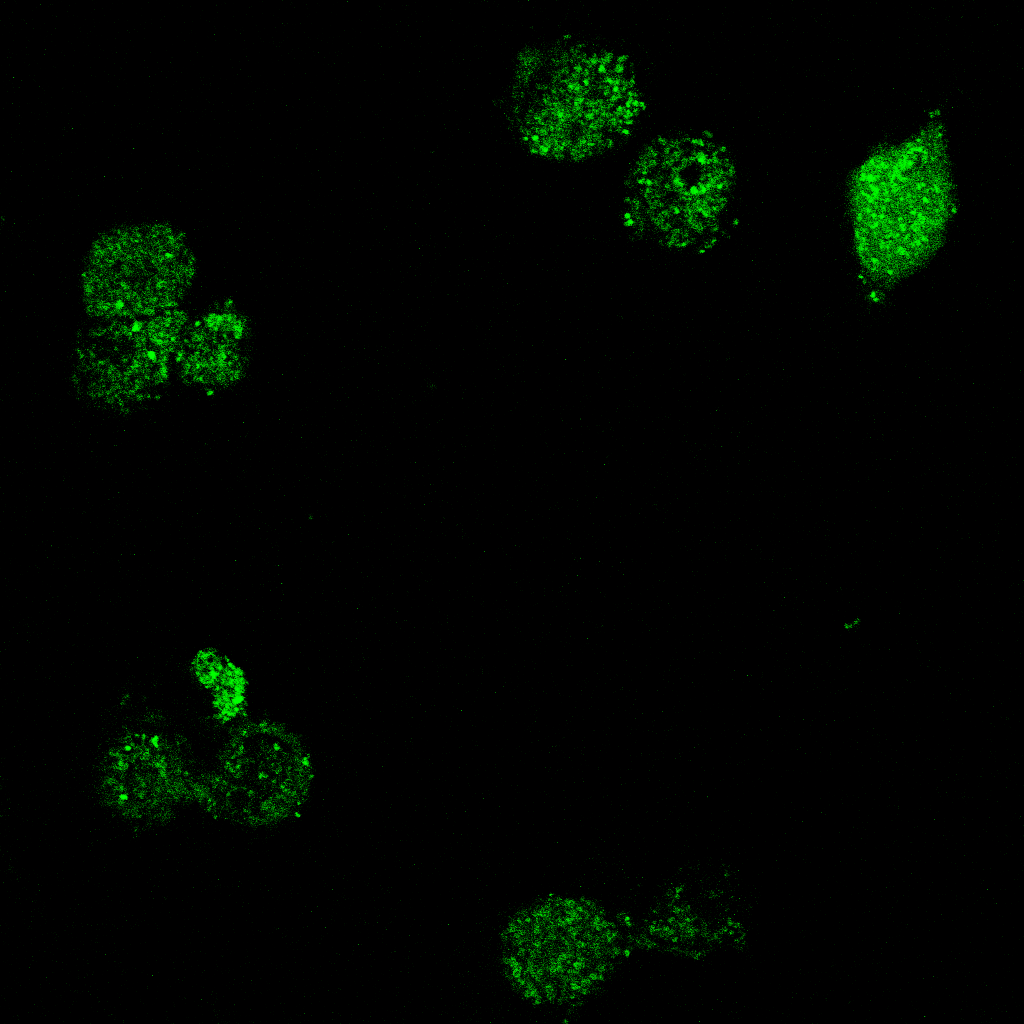

Supplement: Supplementary file 5 [file DataSheet2.ZIP › Original source data 2/FIGURE3.C RED TO GREEN/Ctrl+Ischemia/GREEN.tif]

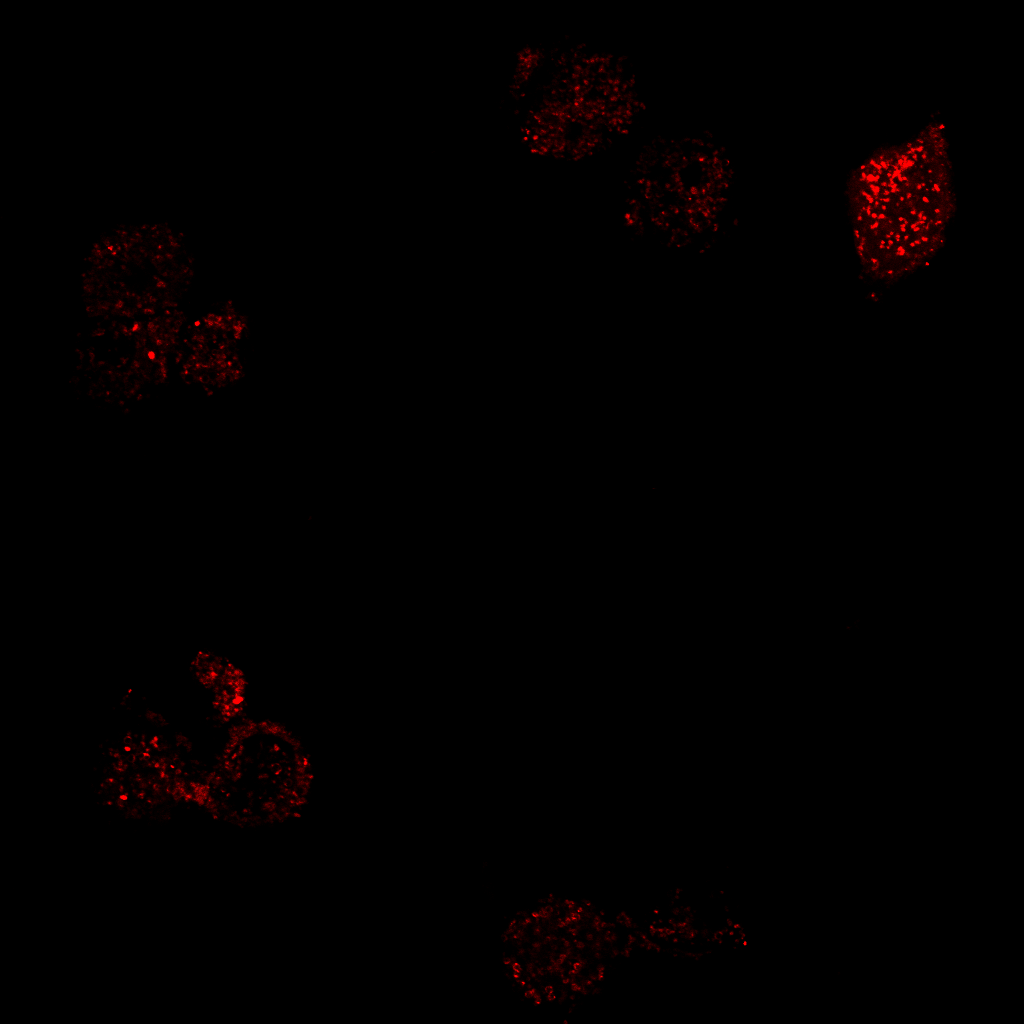

Supplement: Supplementary file 5 [file DataSheet2.ZIP › Original source data 2/FIGURE3.C RED TO GREEN/Ctrl+Ischemia/RED.tif]

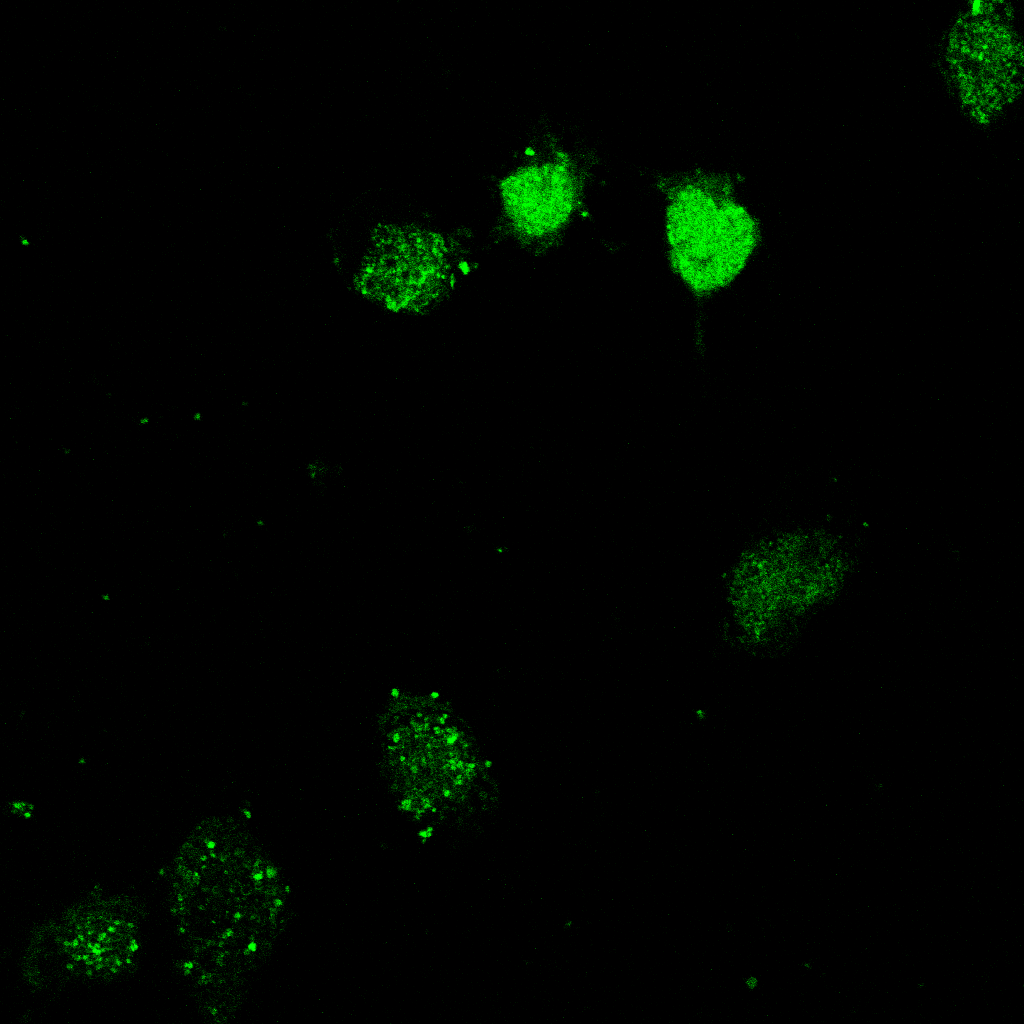

Supplement: Supplementary file 5 [file DataSheet2.ZIP › Original source data 2/FIGURE3.C RED TO GREEN/Sen/GREEN.tif]

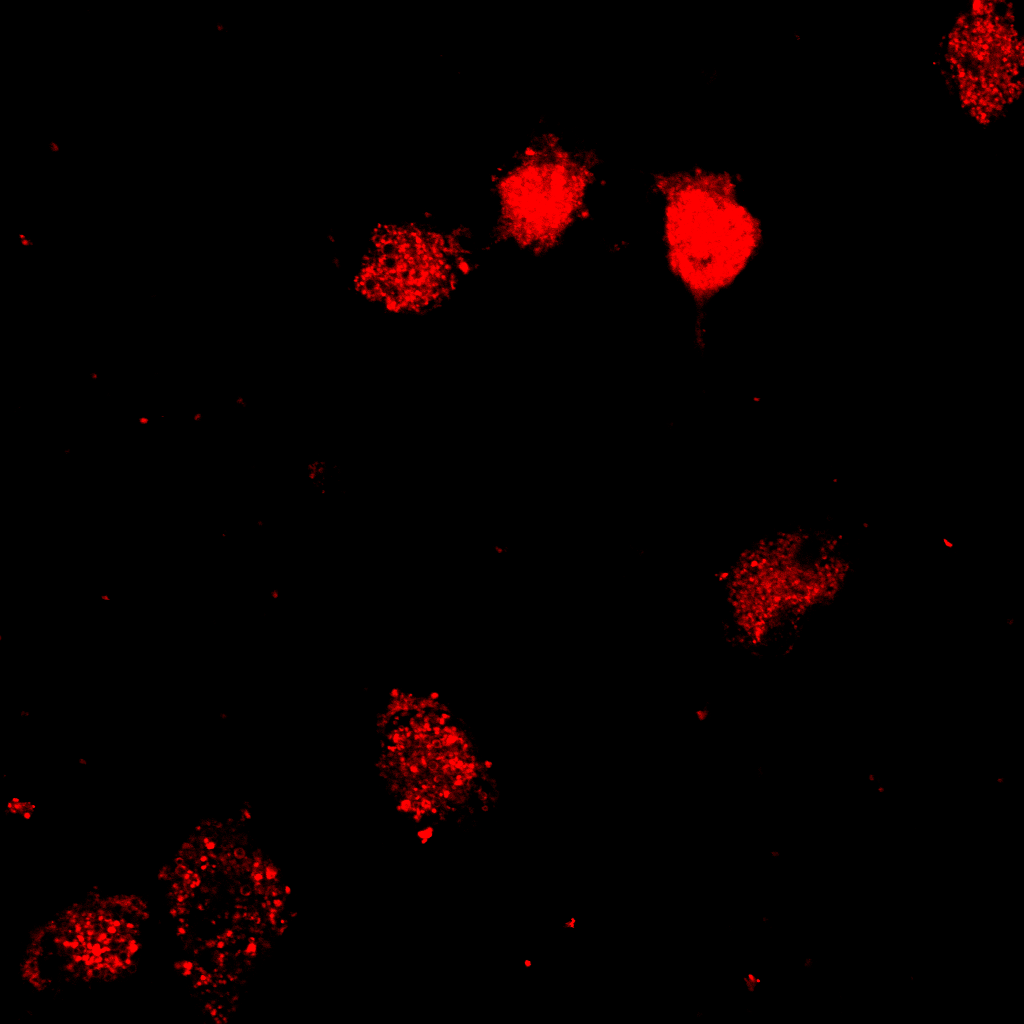

Supplement: Supplementary file 5 [file DataSheet2.ZIP › Original source data 2/FIGURE3.C RED TO GREEN/Sen/RED.tif]

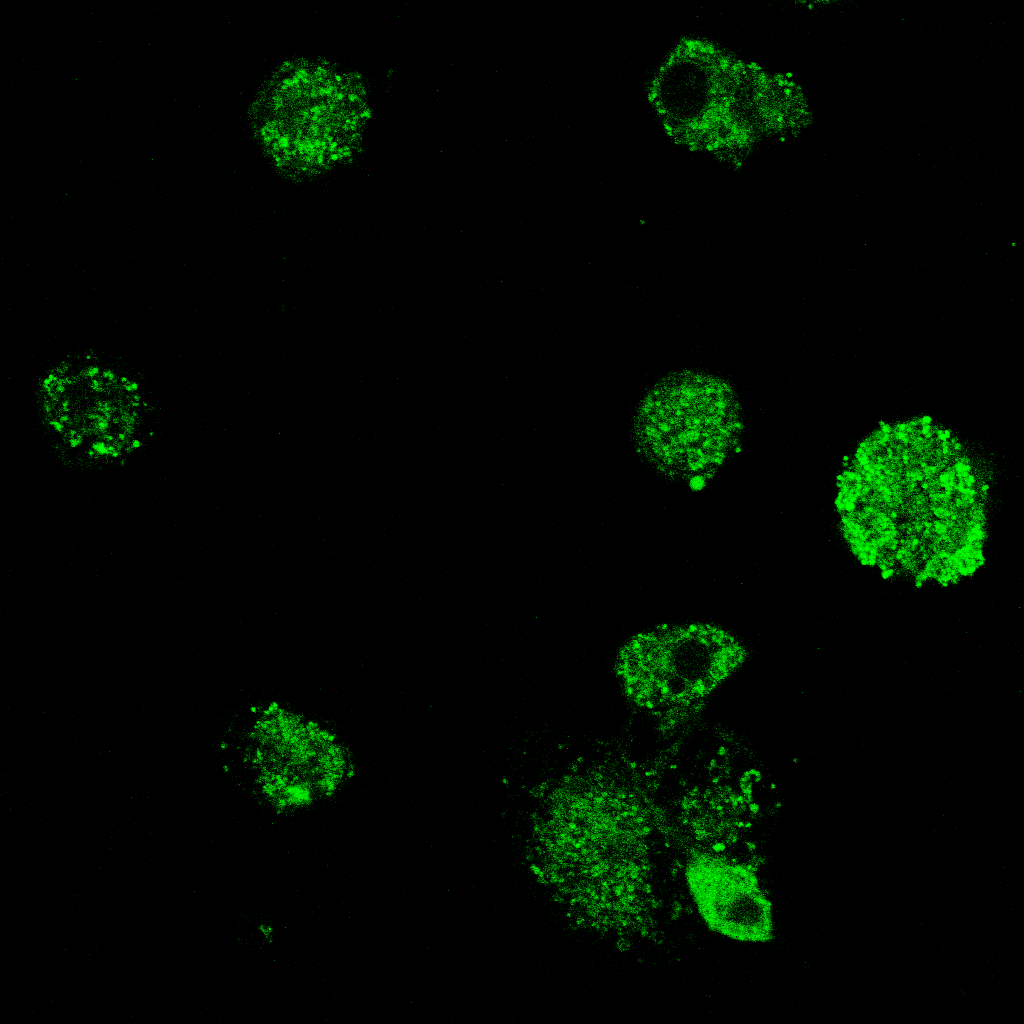

Supplement: Supplementary file 5 [file DataSheet2.ZIP › Original source data 2/FIGURE3.C RED TO GREEN/Sen+Ischemia/GREEN.tif]

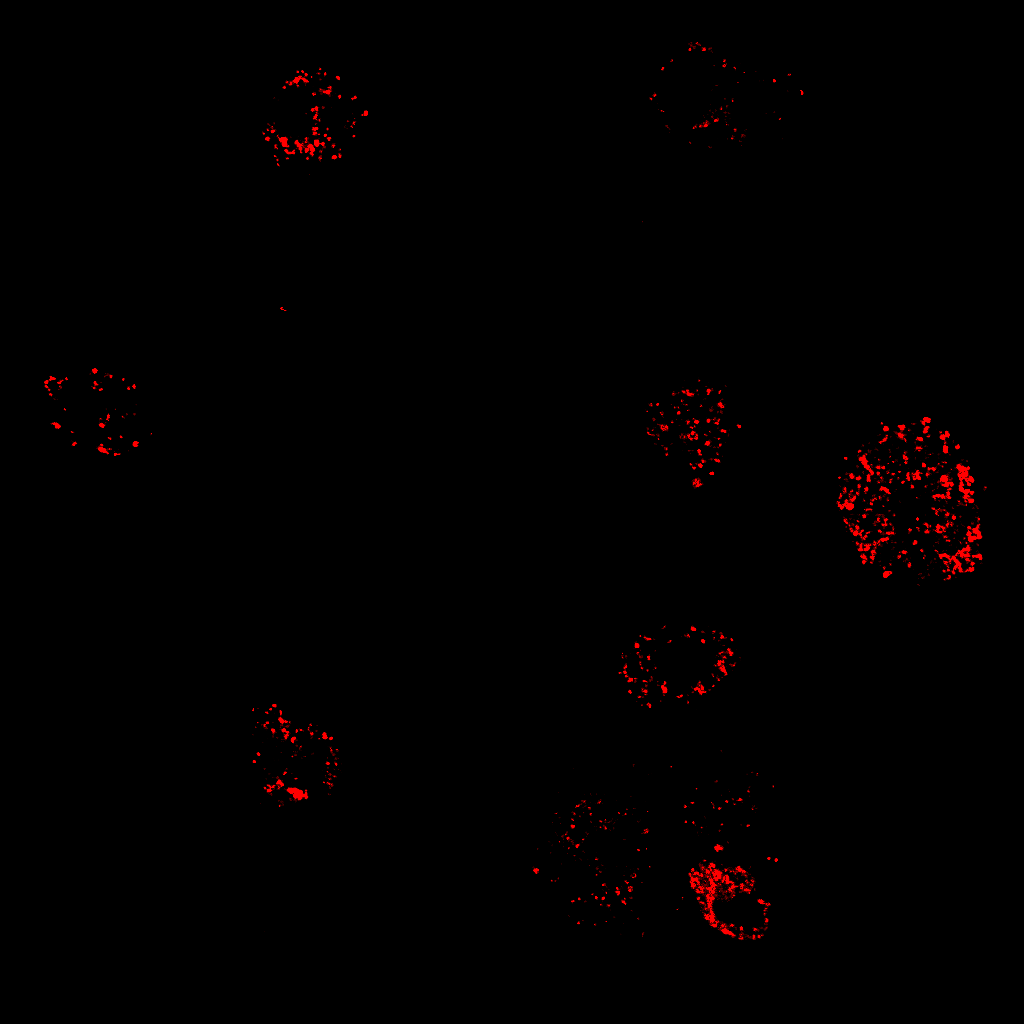

Supplement: Supplementary file 5 [file DataSheet2.ZIP › Original source data 2/FIGURE3.C RED TO GREEN/Sen+Ischemia/RED.tif]
