# Supplementary figures and images for: Wnt 3a Protects Myocardial Injury in Elderly Acute Myocardial Infarction by Inhibiting Serum Cystatin C/ROS-Induced Mitochondrial Damage (part 2 of 2)
Source: Front Physiol. 2022 Jul 22;13:950960. doi: 10.3389/fphys.2022.950960 (PMC9355253; doi:10.3389/fphys.2022.950960)

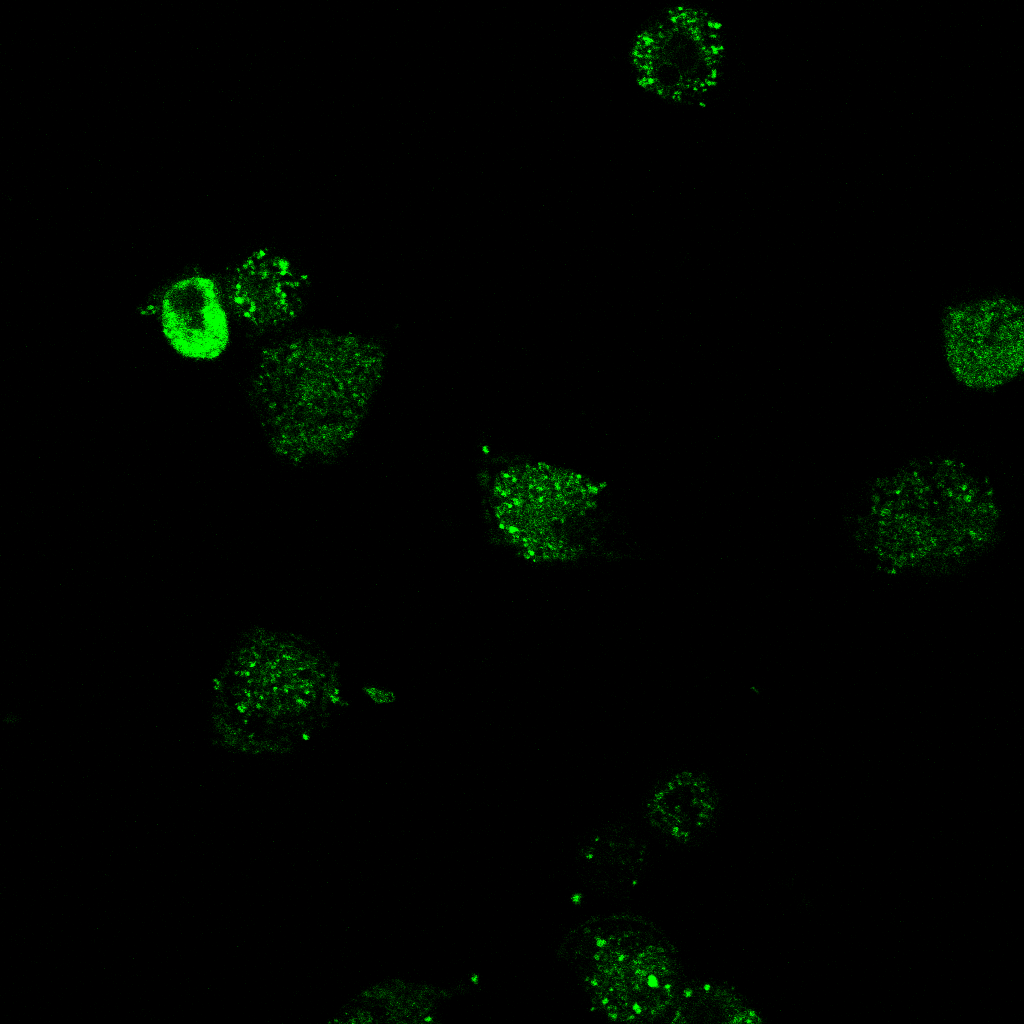

Supplement: Supplementary file 5 [file DataSheet2.ZIP › Original source data 2/FIGURE3.C RED TO GREEN/Sen+Ischemia+NAC/GREEN.tif]

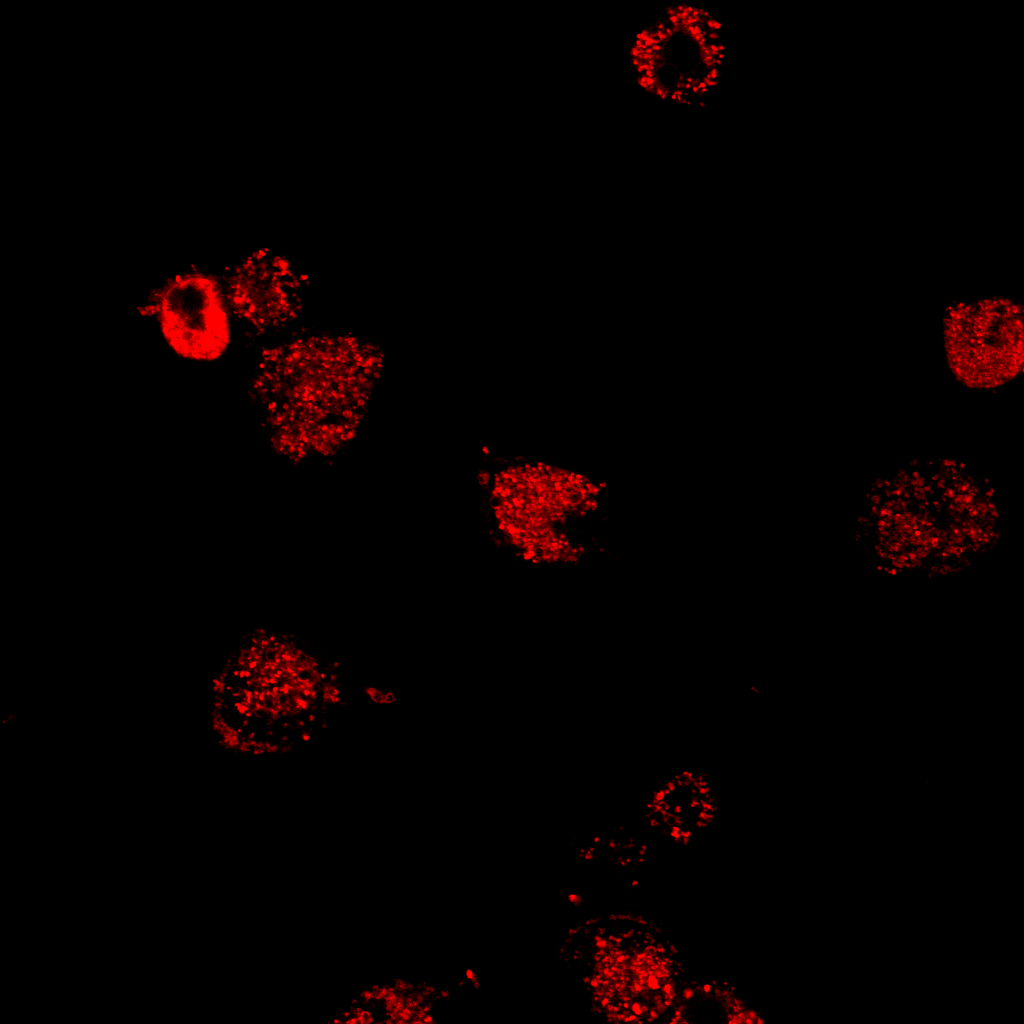

Supplement: Supplementary file 5 [file DataSheet2.ZIP › Original source data 2/FIGURE3.C RED TO GREEN/Sen+Ischemia+NAC/RED.tif]

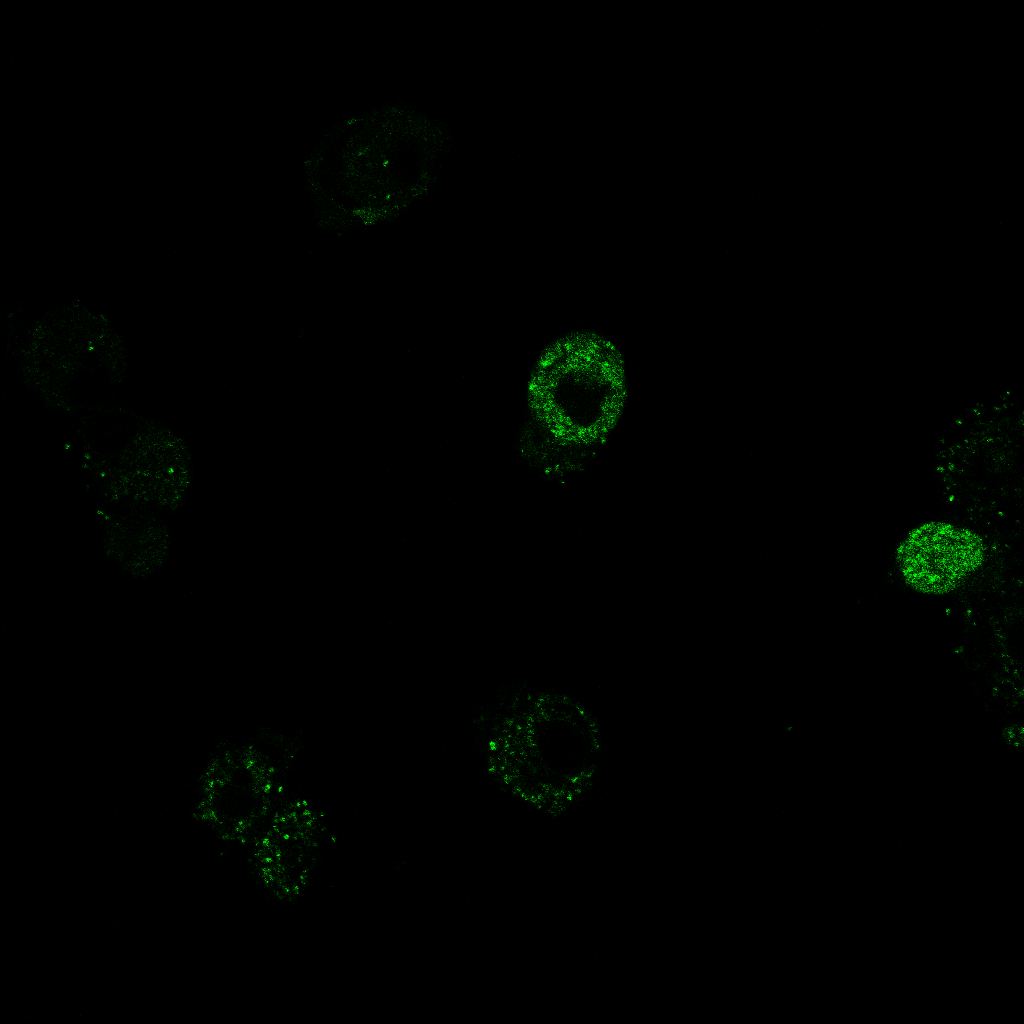

Supplement: Supplementary file 5 [file DataSheet2.ZIP › Original source data 2/FIGURE3.C RED TO GREEN/Sen+Ischemia+SiCys/GREEN.tif]

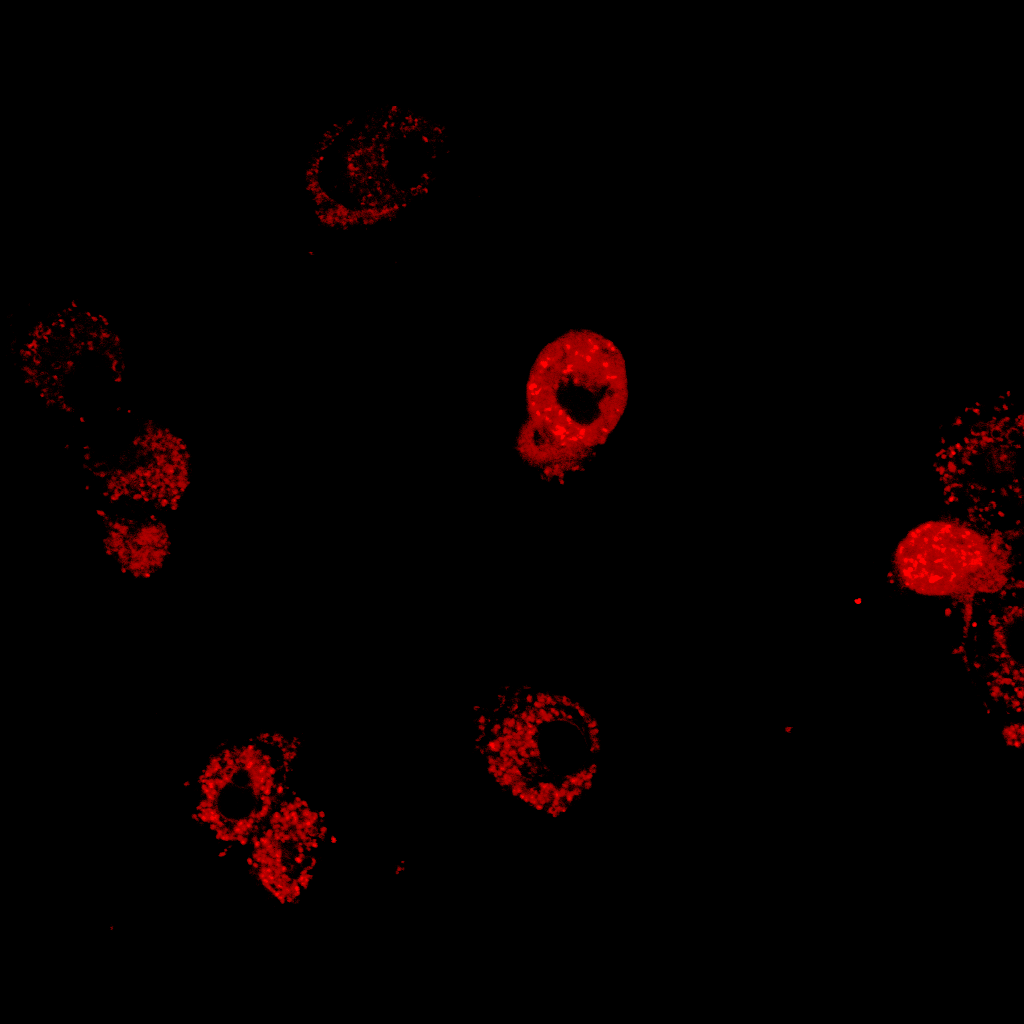

Supplement: Supplementary file 5 [file DataSheet2.ZIP › Original source data 2/FIGURE3.C RED TO GREEN/Sen+Ischemia+SiCys/RED.tif]

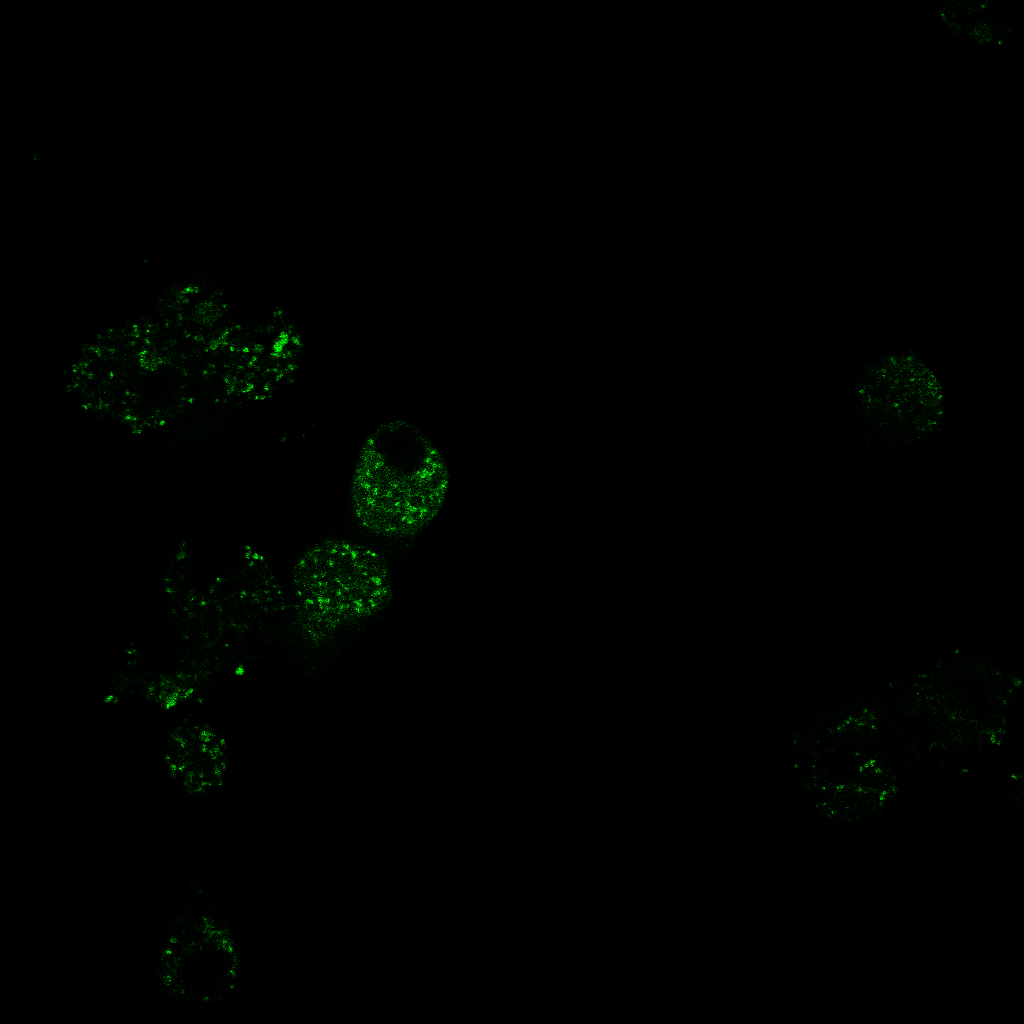

Supplement: Supplementary file 5 [file DataSheet2.ZIP › Original source data 2/FIGURE3.C RED TO GREEN/Sen+Ischemia+Wnt3a/GREEN.tif]

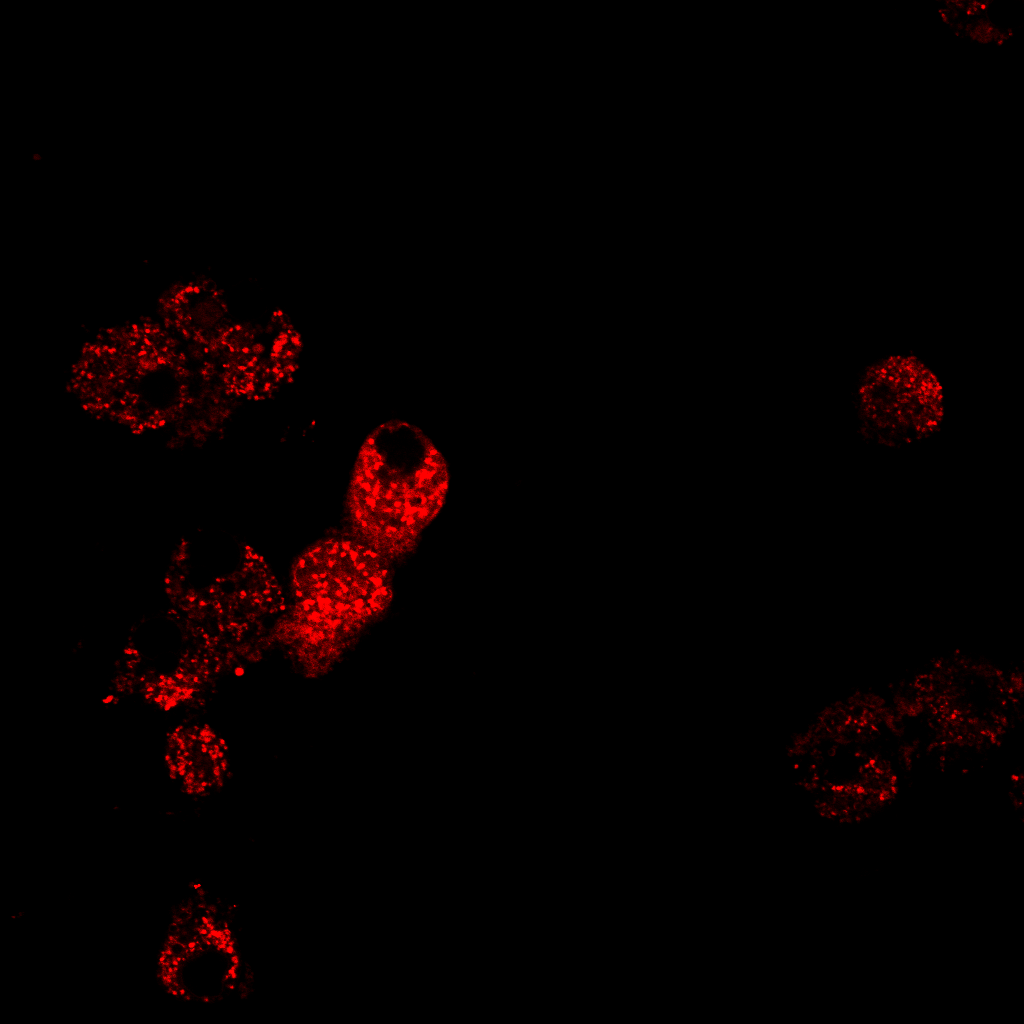

Supplement: Supplementary file 5 [file DataSheet2.ZIP › Original source data 2/FIGURE3.C RED TO GREEN/Sen+Ischemia+Wnt3a/RED.tif]

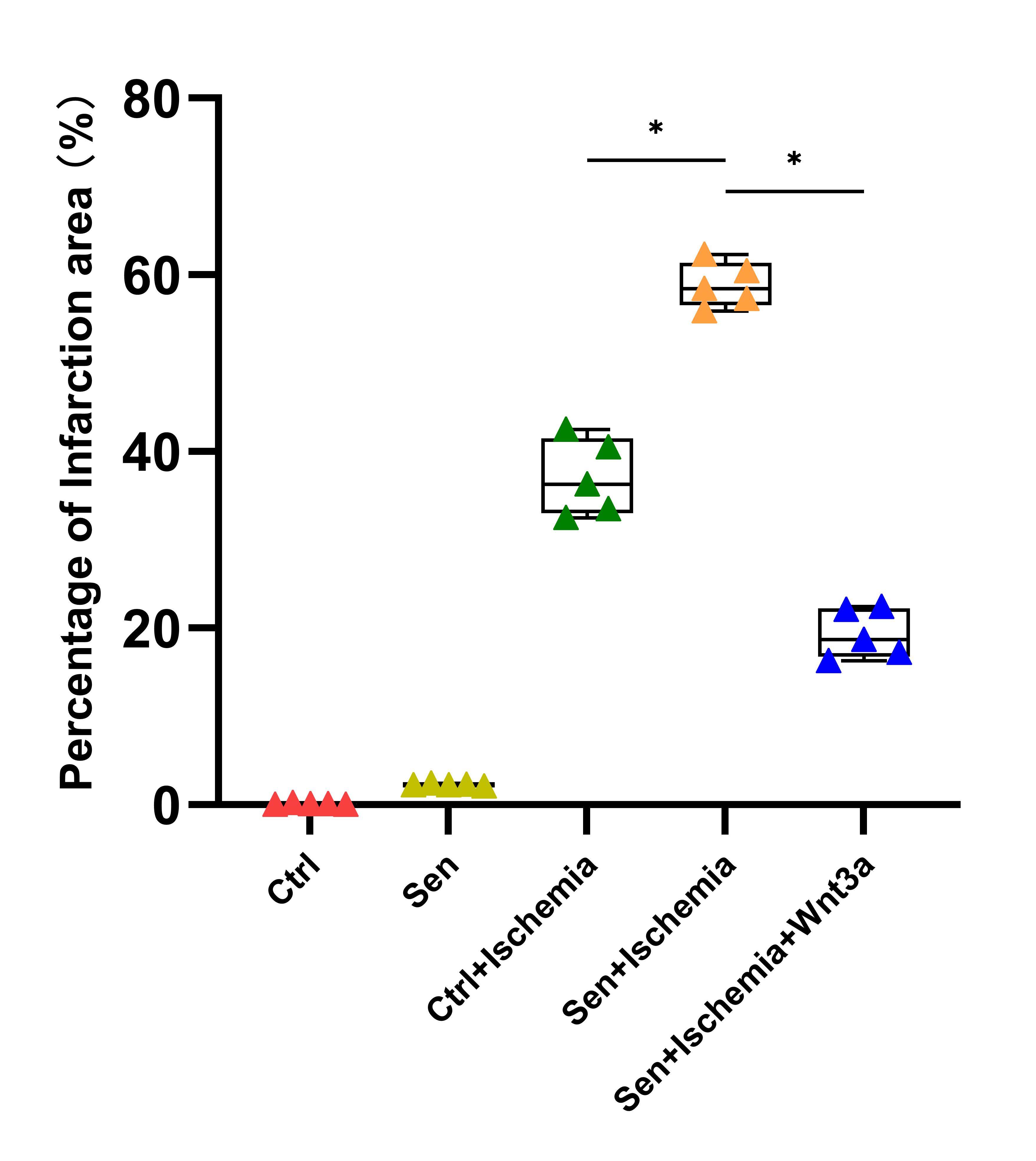

Supplement: Supplementary file 6 [file DataSheet5.ZIP › Prism data and figures-R1/FIGURE1.C.jpg]

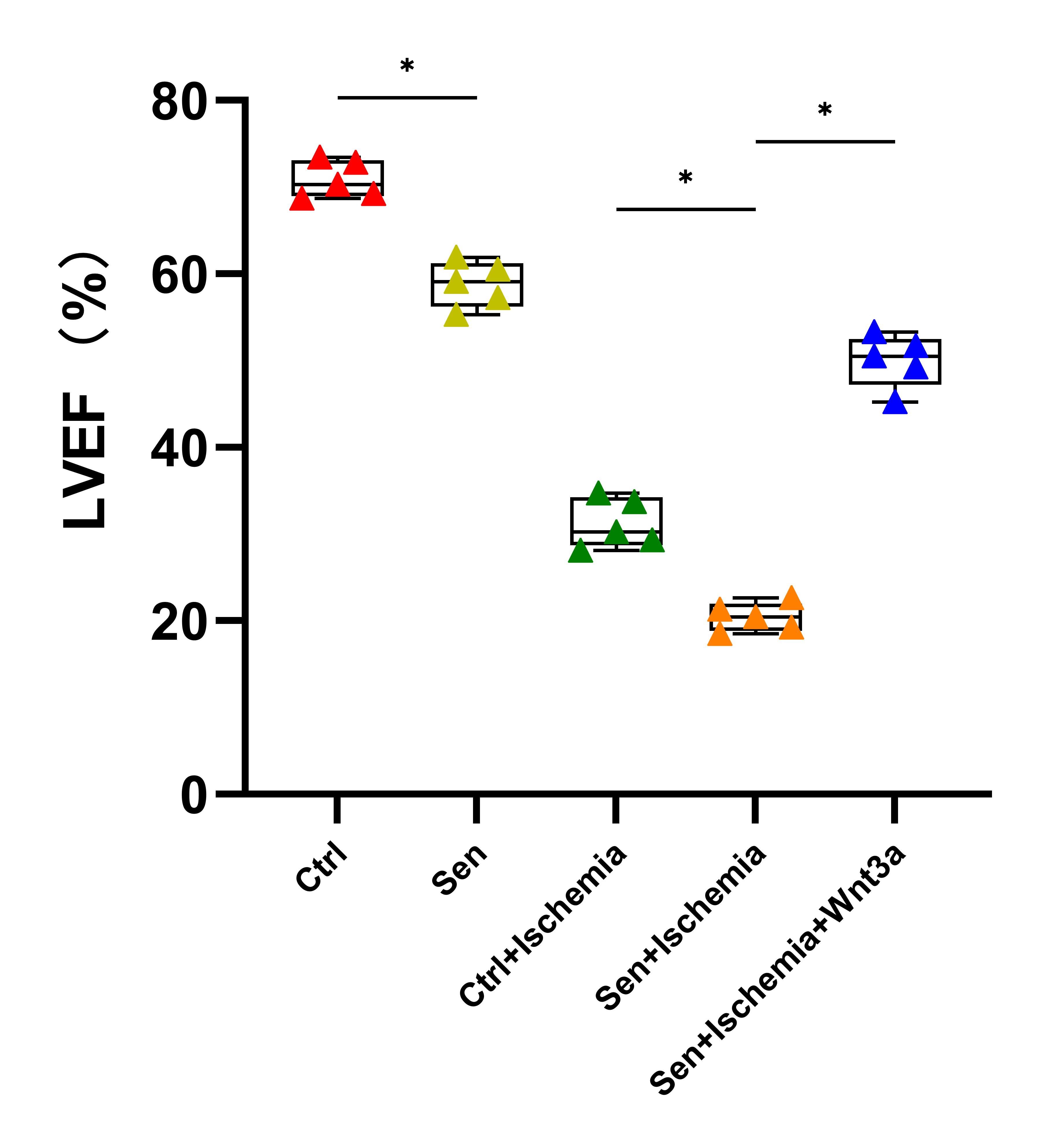

Supplement: Supplementary file 6 [file DataSheet5.ZIP › Prism data and figures-R1/FIGURE1.D.jpg]

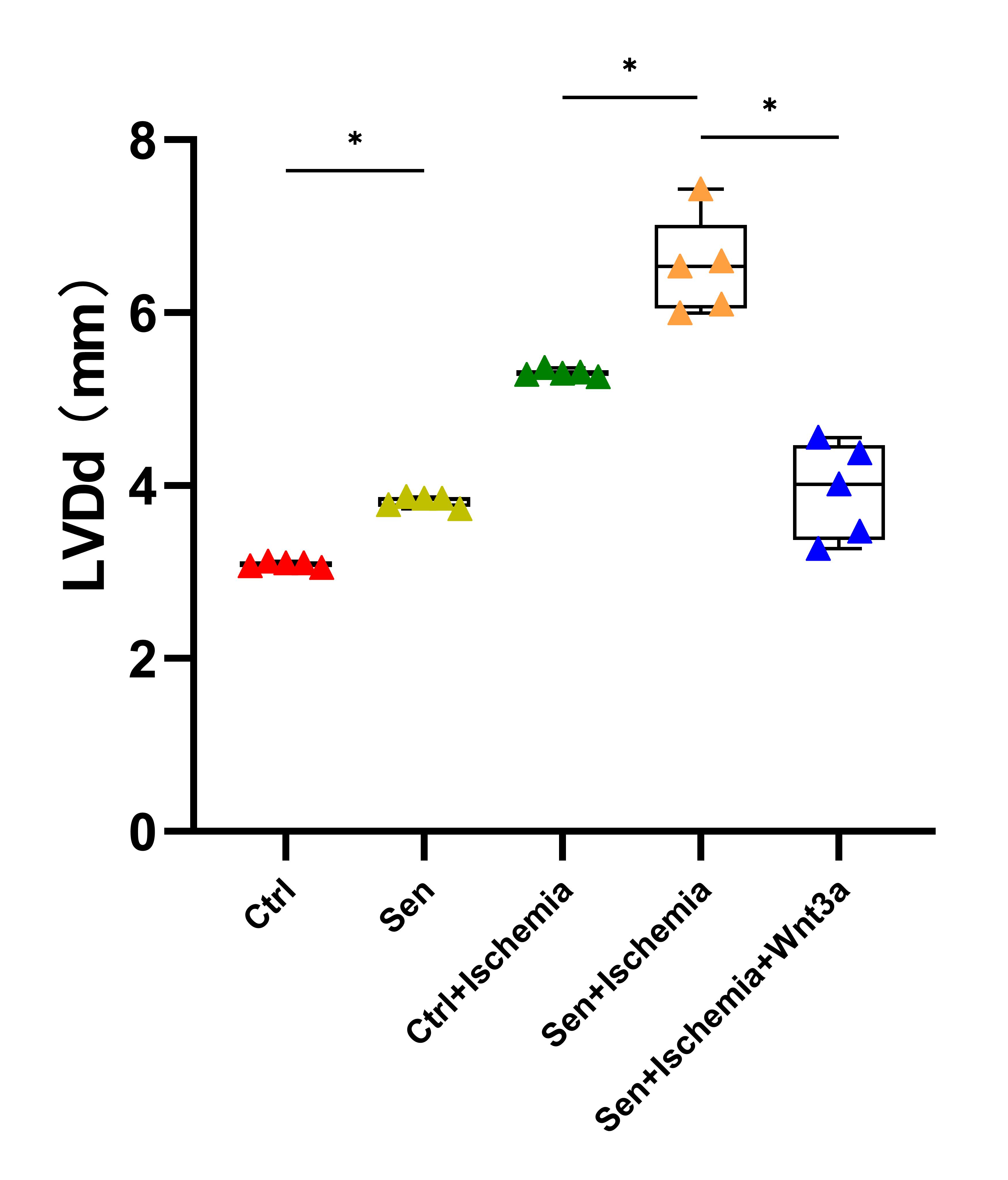

Supplement: Supplementary file 6 [file DataSheet5.ZIP › Prism data and figures-R1/FIGURE1.E.jpg]

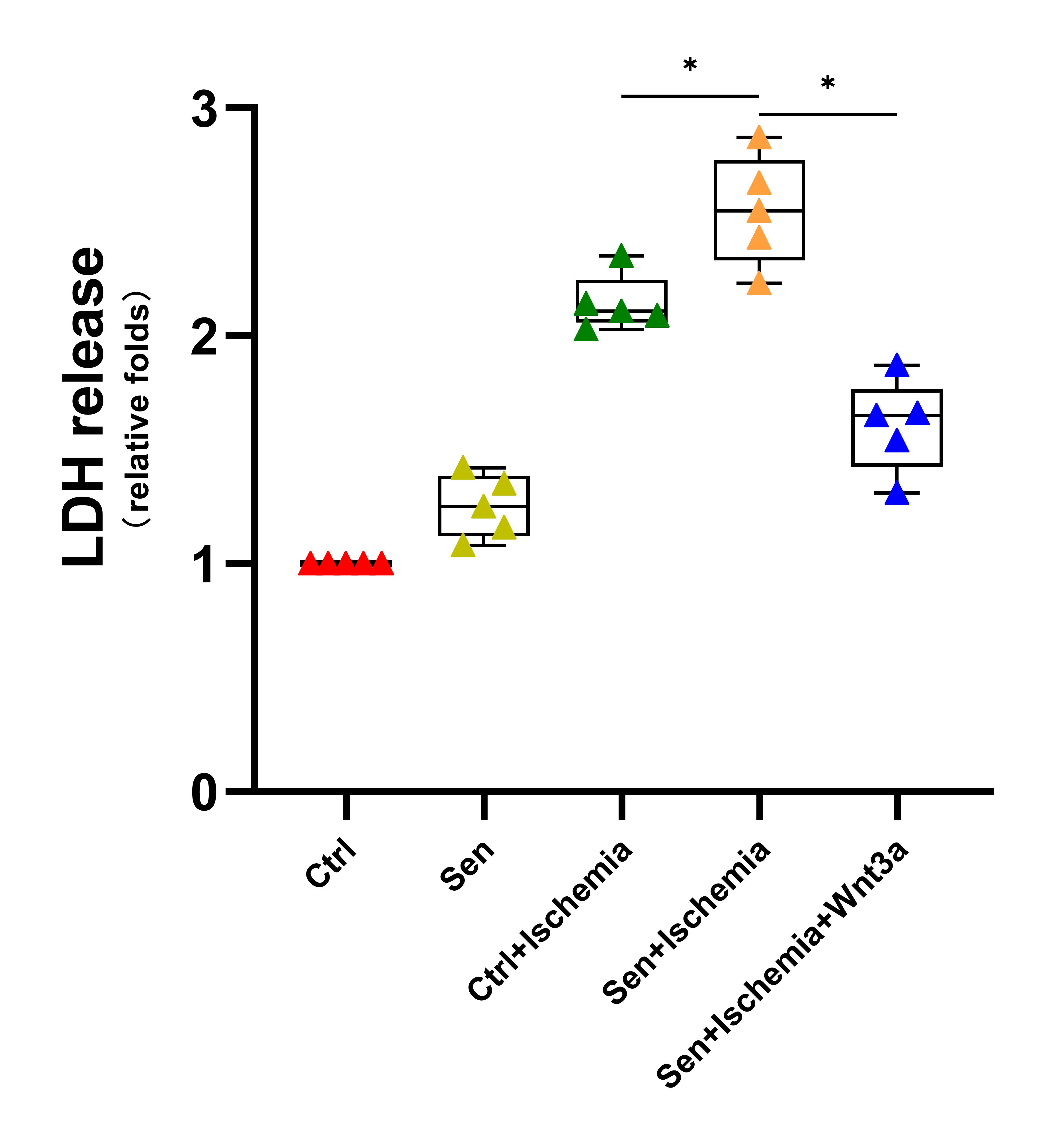

Supplement: Supplementary file 6 [file DataSheet5.ZIP › Prism data and figures-R1/FIGURE1.F.jpg]

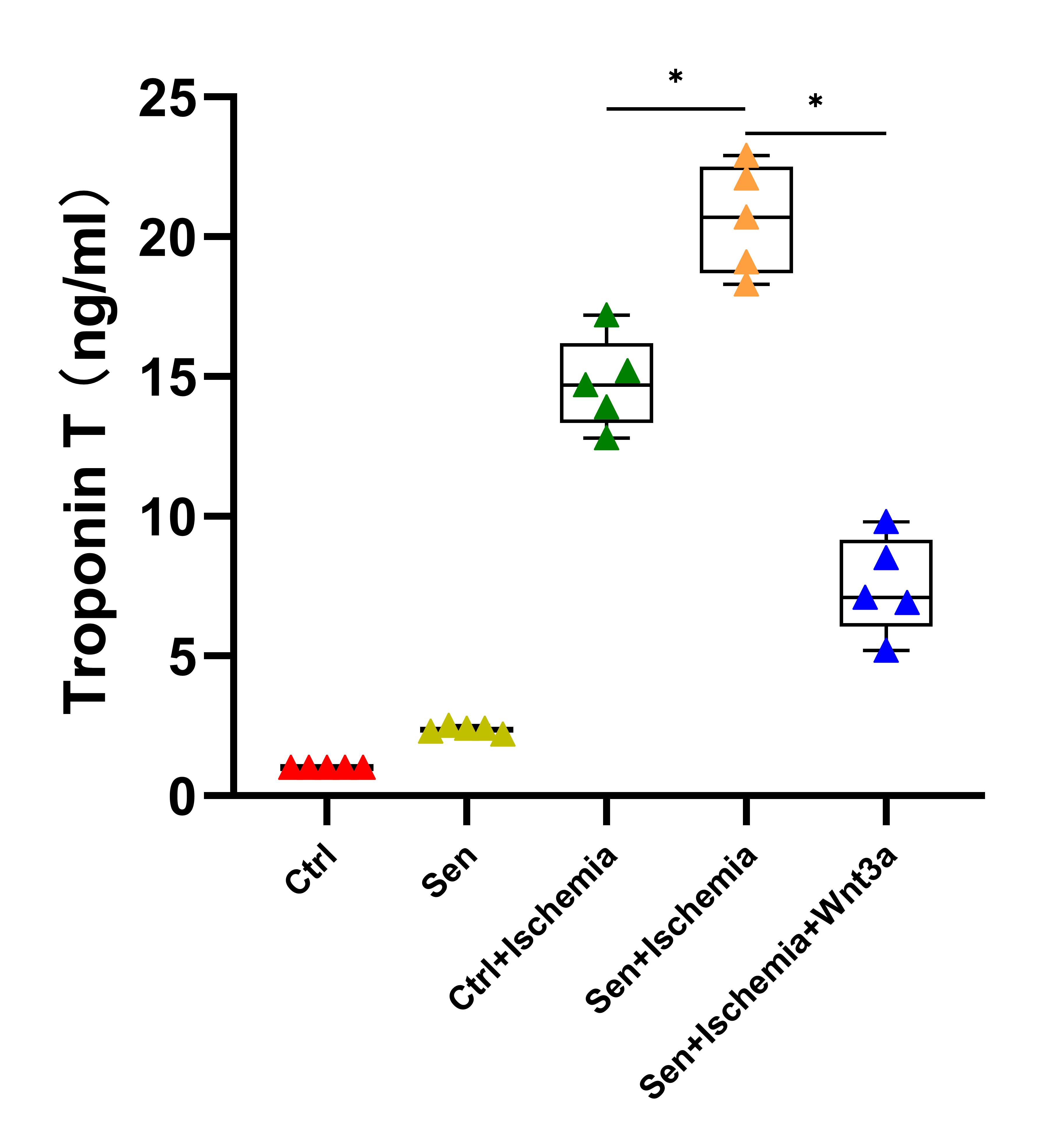

Supplement: Supplementary file 6 [file DataSheet5.ZIP › Prism data and figures-R1/FIGURE1.G.jpg]

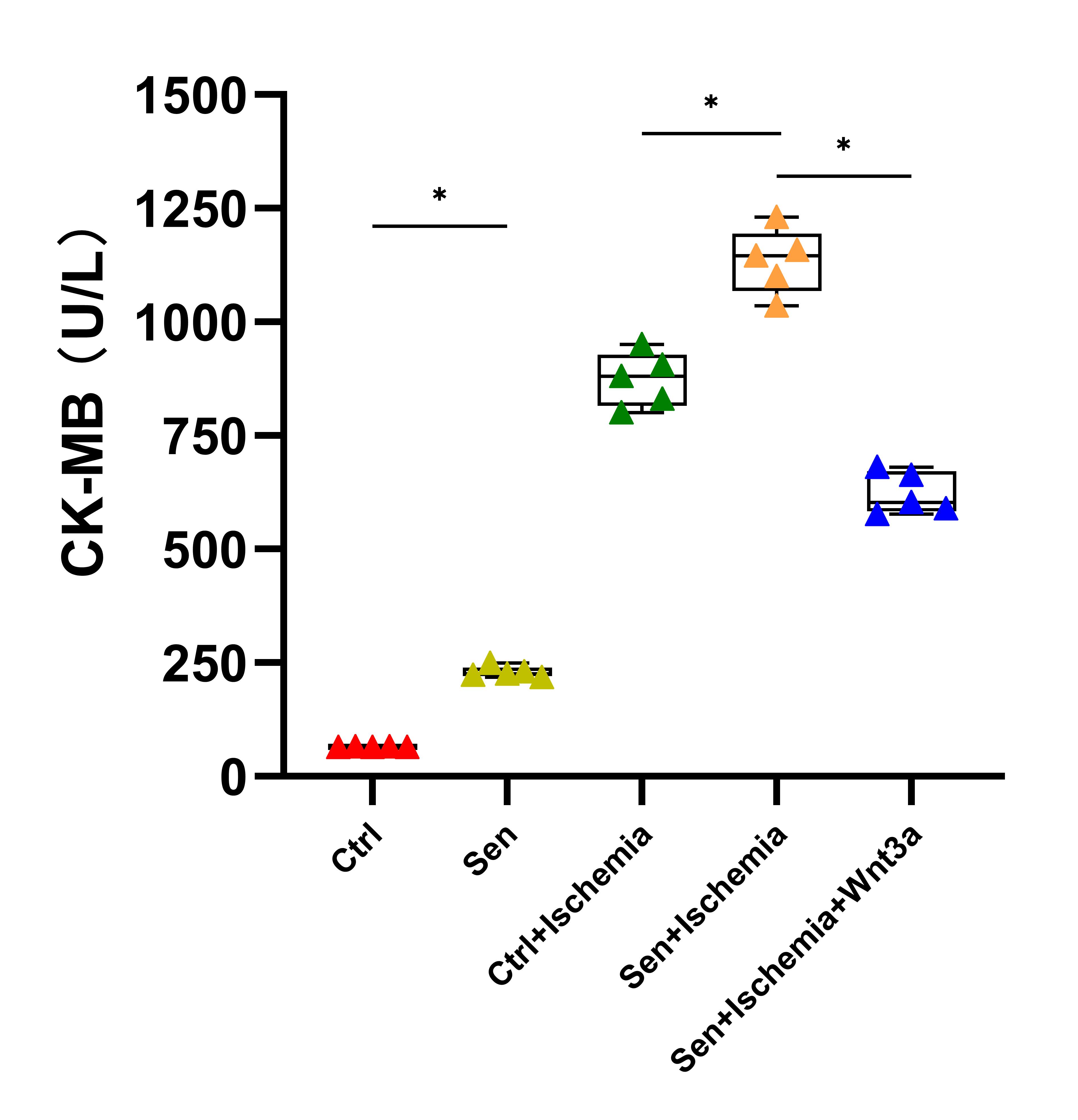

Supplement: Supplementary file 6 [file DataSheet5.ZIP › Prism data and figures-R1/FIGURE1.H.jpg]

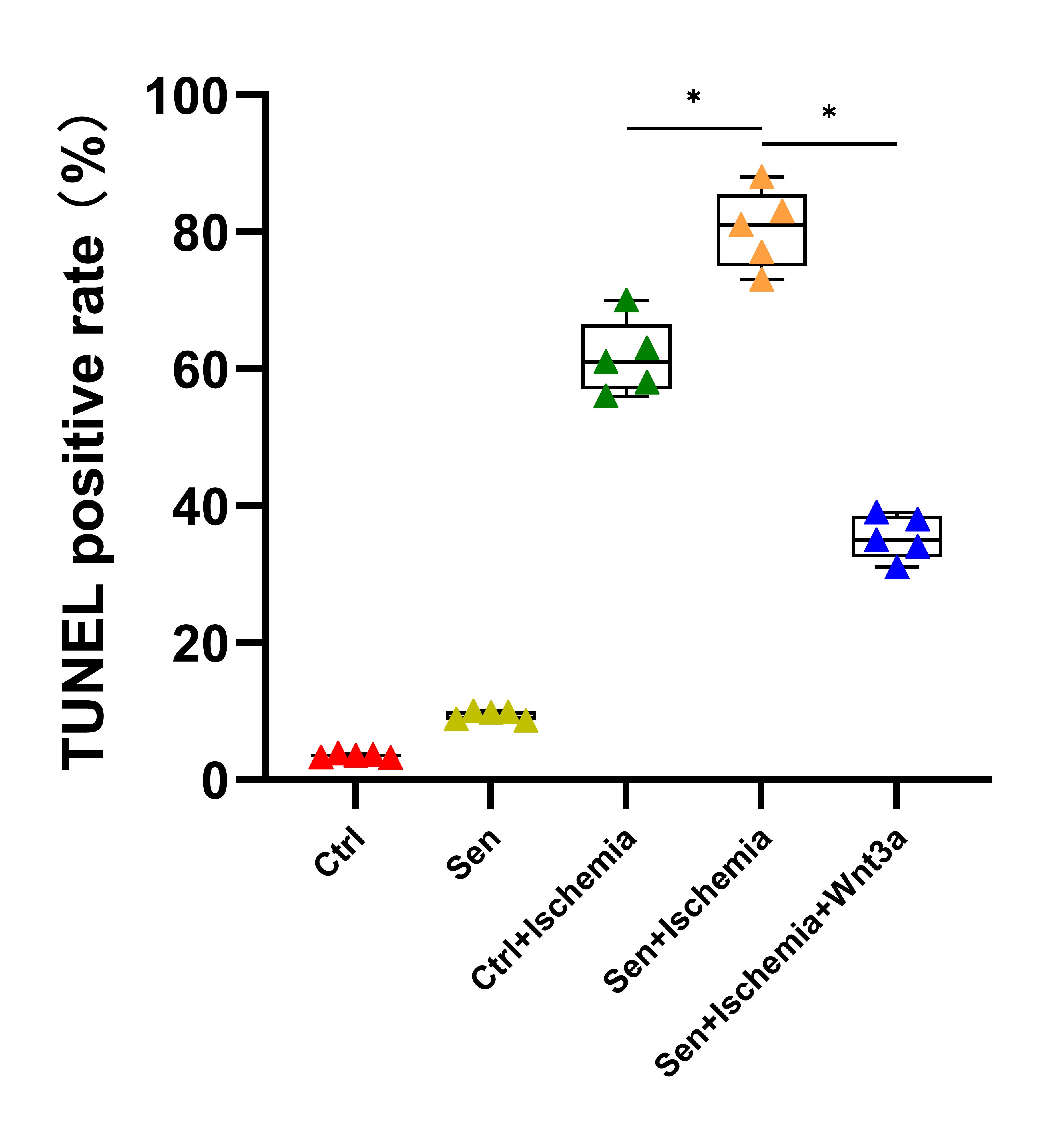

Supplement: Supplementary file 6 [file DataSheet5.ZIP › Prism data and figures-R1/FIGURE2.E.jpg]

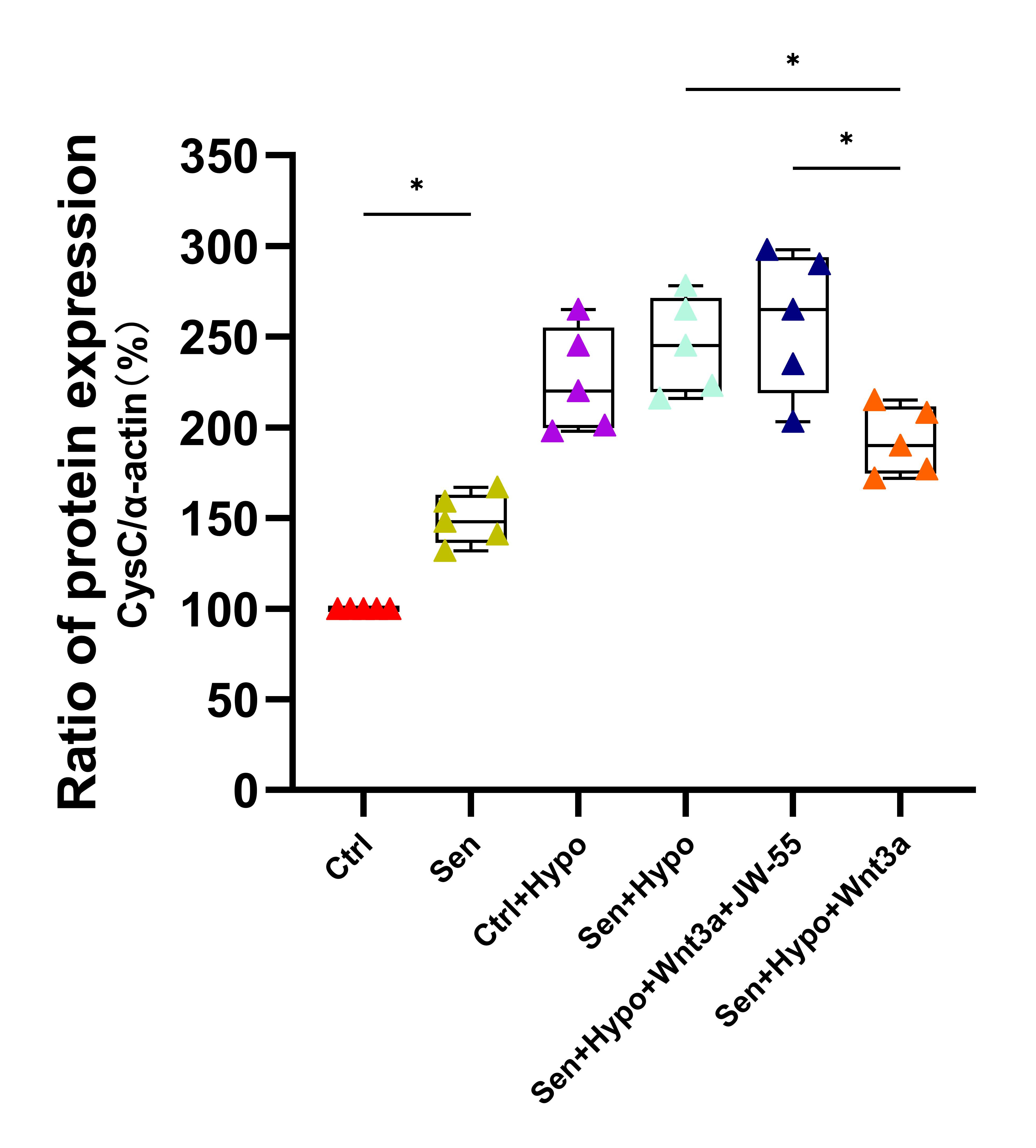

Supplement: Supplementary file 6 [file DataSheet5.ZIP › Prism data and figures-R1/FIGURE2.F.jpg]

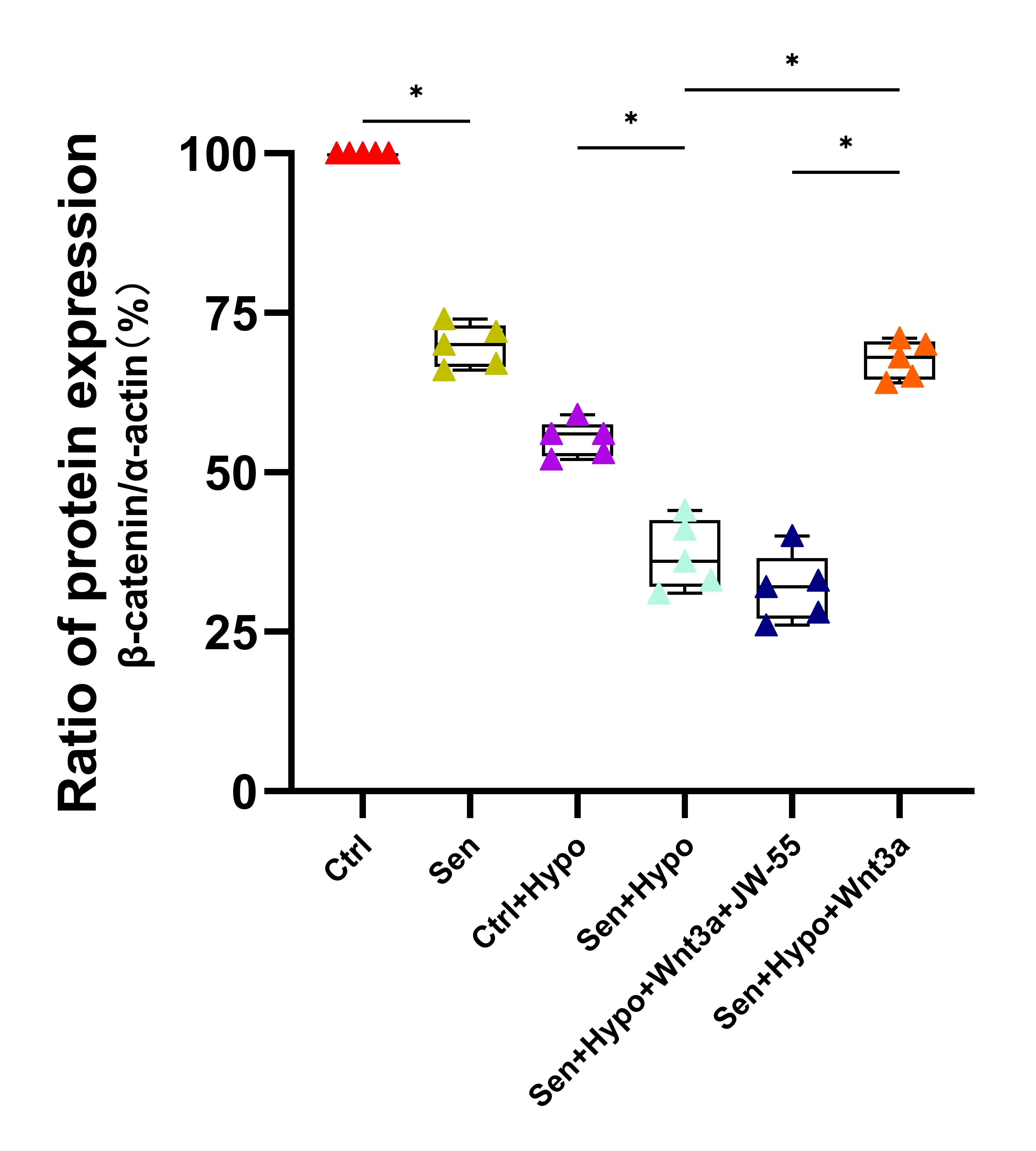

Supplement: Supplementary file 6 [file DataSheet5.ZIP › Prism data and figures-R1/FIGURE2.G.jpg]

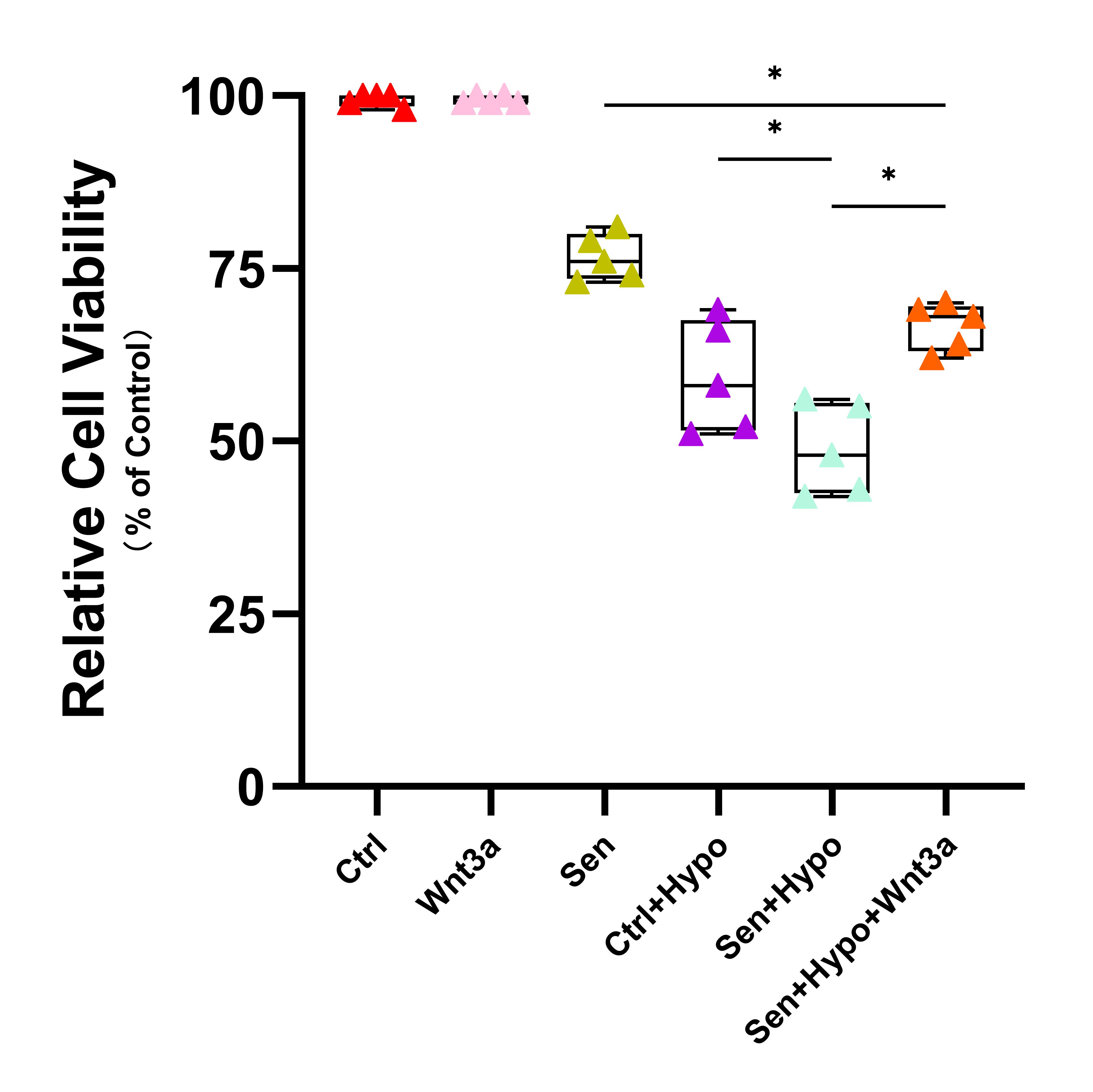

Supplement: Supplementary file 6 [file DataSheet5.ZIP › Prism data and figures-R1/FIGURE2.H.jpg]

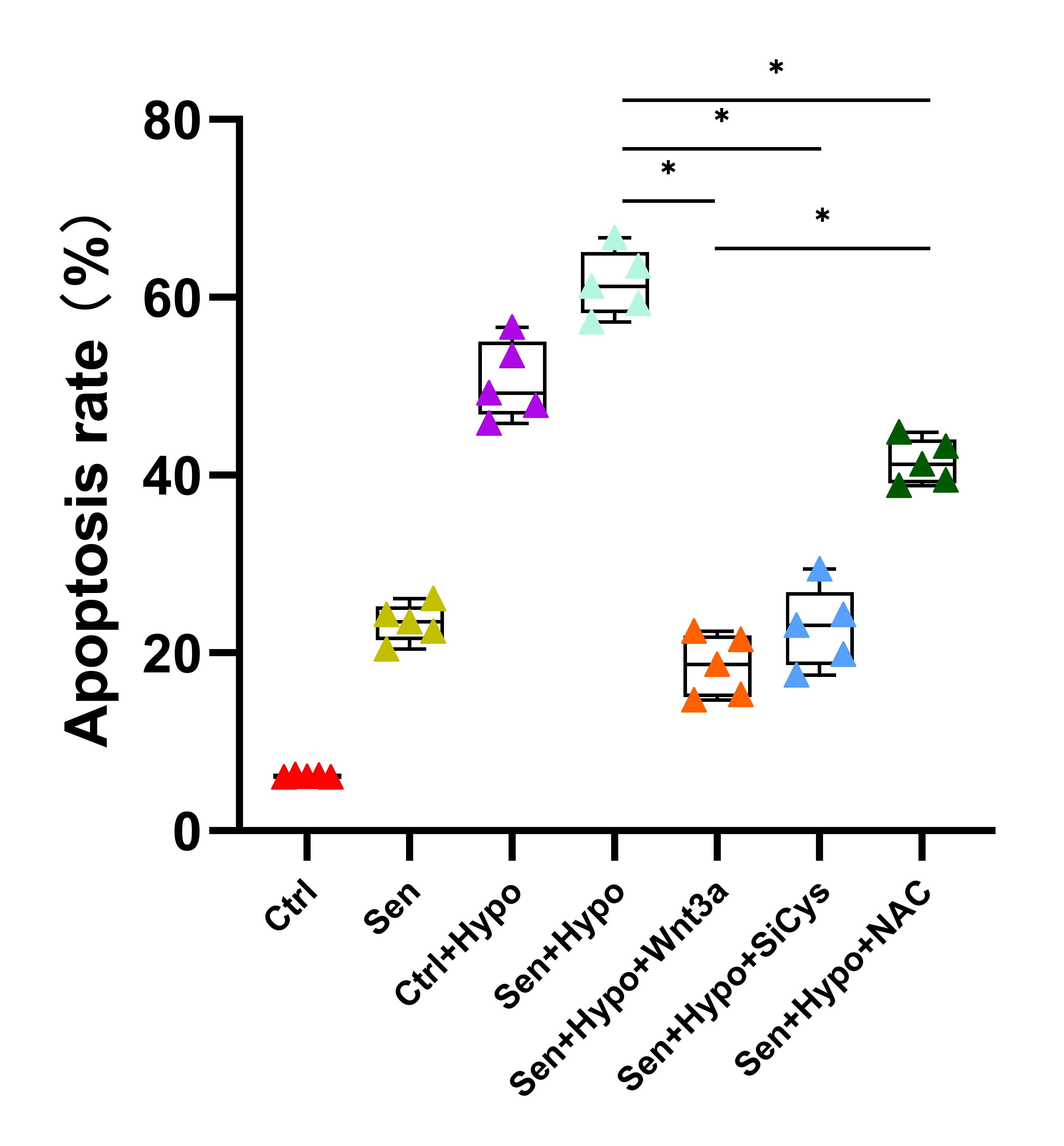

Supplement: Supplementary file 6 [file DataSheet5.ZIP › Prism data and figures-R1/FIGURE3.D.jpg]

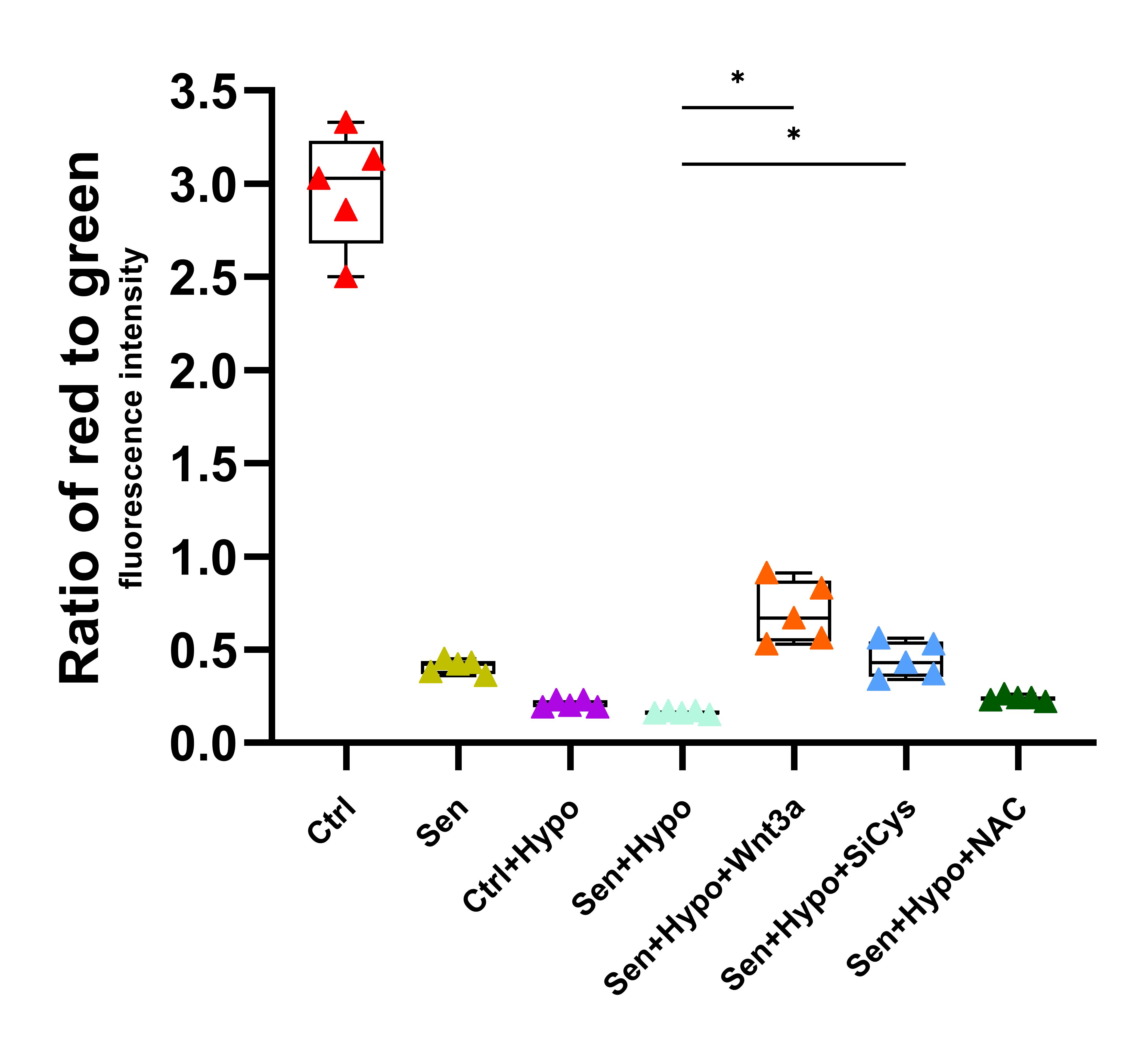

Supplement: Supplementary file 6 [file DataSheet5.ZIP › Prism data and figures-R1/FIGURE3.E.jpg]

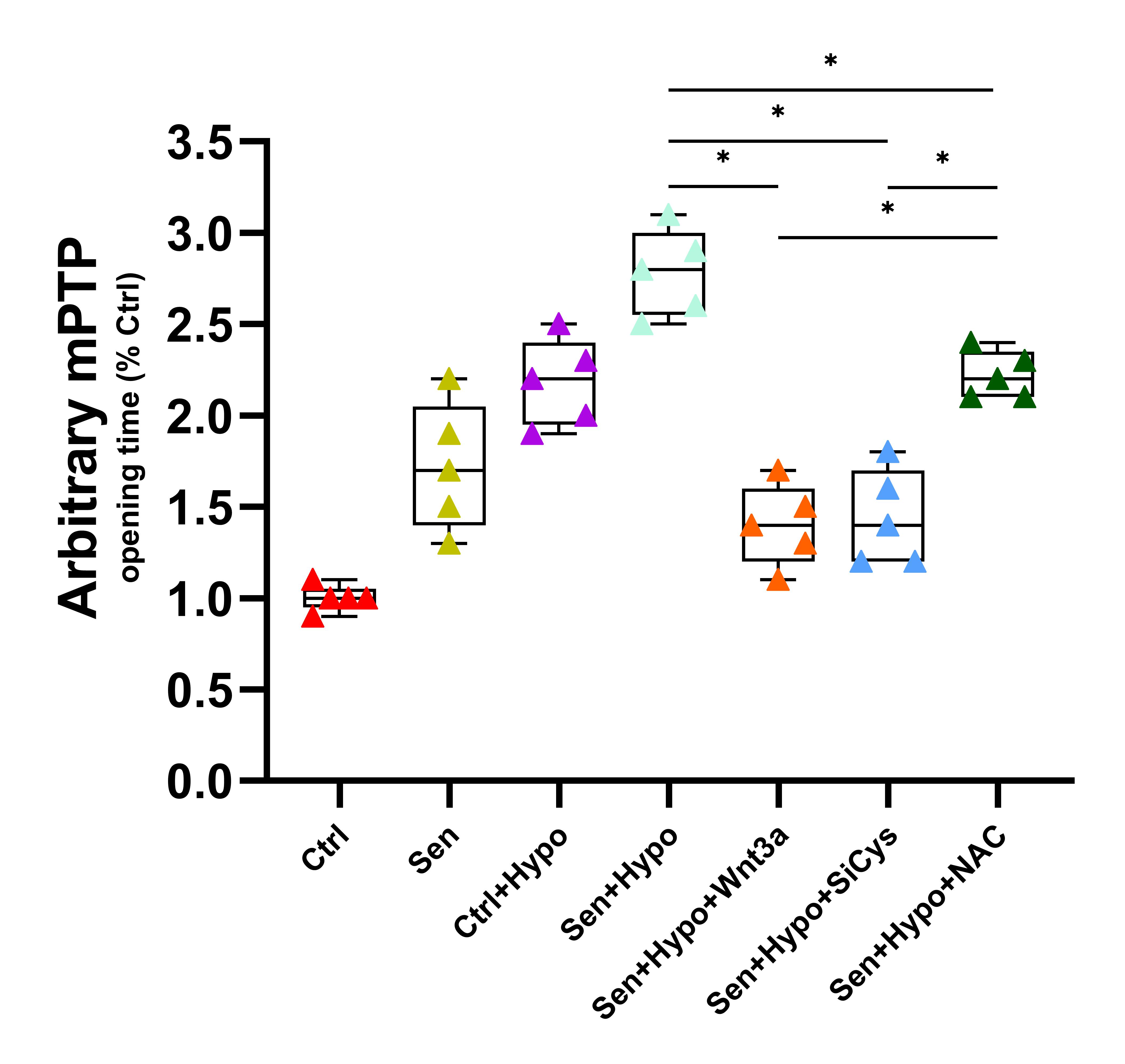

Supplement: Supplementary file 6 [file DataSheet5.ZIP › Prism data and figures-R1/FIGURE3.F.jpg]
